# Bleeding in haemorrhagic fever with renal syndrome: A systematic review characterising the loss of haemostasis in hantavirus infections

**DOI:** 10.1371/journal.pntd.0014524

**Published:** 2026-07-15

**Authors:** Matthew J. Riley, Aliza Hudda, Miša Korva, Tatjana Avšič-Županc, Beverley J. Hunt, Tom E. Fletcher

**Affiliations:** 1 Department of Clinical Sciences, Liverpool School of Tropical Medicine, Liverpool, United Kingdom; 2 Institute of Microbiology and Immunology, Faculty of Medicine, University of Ljubljana, Ljubljana, Slovenia; 3 Thrombosis and Haemophilia Centre, Guy’s and St. Thomas’ NHS Trust, London, United Kingdom; Wuhan Institute of Virology CAS: Chinese Academy of Sciences Wuhan Institute of Virology, CHINA

## Abstract

**Background:**

Haemorrhagic fever with renal syndrome (HFRS) is caused by hantaviruses and is associated with variable thrombocytopenia, coagulation abnormalities, and bleeding manifestations. While clinical features have been described in multiple studies, no previous systematic review has synthesised evidence on haemostatic dysfunction in HFRS. This review aimed to characterise the clinical and laboratory features of coagulation disturbances in HFRS to inform understanding of disease mechanisms and future research.

**Methodology/principal findings:**

We systematically searched MEDLINE, PubMed, CINAHL, Web of Science, and Scopus on 12 December 2024 for studies reporting clinical or laboratory evidence of haemostatic dysfunction in confirmed HFRS. The review protocol was prospectively registered on PROSPERO (CRD42024618760). Observational studies and case series meeting predefined inclusion criteria were eligible. Risk of bias was assessed using the Joanna Briggs Institute (JBI) critical appraisal tools and, for one before–after cohort study, the National Institutes of Health (NIH) quality assessment tool. Studies were graded from 1 (lowest) to 3 (highest) based on methodological quality. Due to heterogeneity in study design and outcome reporting, meta-analysis was not feasible. Weighted averages were calculated for laboratory parameters, and data were visualised using bubble and bar plots. Fifty-five studies comprising 7,950 patients were included. Thrombocytopenia was the most consistent abnormality, typically more pronounced in infections with Hantaan (HTNV), Dobrava (DOBV), and Seoul (SEOV) viruses compared with Puumala (PUUV), which was associated with milder disease. Mild prolongation of activated partial thromboplastin time and elevated D-dimer were variably reported. Limited paediatric data suggested milder disease and less pronounced coagulation abnormalities.

**Conclusions/significance:**

This review provides the first systematic synthesis of haemostatic dysfunction in HFRS. Findings highlight more severe thrombocytopenia and bleeding in HTNV, DOBV, and SEOV infections compared with PUUV. Variable study quality and incomplete reporting limit firm conclusions, underscoring the need for standardised, prospective studies of coagulation and bleeding outcomes.

## Author summary

In this study, we reviewed published research describing the blood clotting changes and bleeding problems that occur in people with haemorrhagic fever with renal syndrome (HFRS), an infection caused by hantaviruses. We analysed data from 55 studies including 7,950 patients. We found that low platelet counts were the most common abnormality. These changes were generally more severe in infections caused by certain hantaviruses compared to others. Laboratory findings such as changes in blood clotting times and markers of abnormal clot formation varied widely between studies. Reports of bleeding ranged from mild, such as nosebleeds, to severe, including bleeding into the gut or brain. Few studies included children, but the data we found suggested they tend to have less severe disease. Overall, we found that although many studies reported important clinical and laboratory information, differences in study quality and how results were measured and presented make it difficult to compare findings directly. Our work highlights the need for future studies that use standardised approaches to measuring and reporting blood clotting changes and bleeding in HFRS. These improvements could help researchers better understand why some people develop severe bleeding and guide improved patient care.

## Introduction

### Introduction to hantaviruses

Haemorrhagic fever with renal syndrome (HFRS) is a zoonotic disease caused by several rodent-borne viruses of the genus *Orthohantavirus* in Europe and Asia. Hantaviruses are members of the family *Hantaviridae*, order *Bunyavirales*, class *Ellioviricetes* [1]. The family *Hantaviridae* is divided into multiple subfamilies and genera that infect a wide range of hosts, including rodents, shrews, moles, bats, reptiles and fish, but all known human pathogens belong to the genus *Orthohantavirus* [2]. Members of this genus are associated with persistent infection in specific rodent reservoirs, from which they are transmitted to humans primarily through the inhalation of aerosolised excreta (urine, faeces, saliva), or less commonly by rodent bites [3,4]. Virions are enveloped, negative-sense, single-stranded RNA viruses with a tri-segmented genome [5]. The order *Bunyavirales* is large and includes several other genera of major public health importance beyond *Hantaviridae*, such as *Nairoviridae* (Crimean–Congo haemorrhagic fever virus), *Arenaviridae* (Lassa virus), and *Phenuiviridae* (Rift Valley fever virus, severe fever with thrombocytopenia syndrome virus) [6], all of which have epidemic potential and cause severe disease in humans.

In Europe and Asia, hantaviruses cause HFRS, whereas in the Americas the clinical syndrome is hantavirus cardiopulmonary syndrome (HCPS) [3]. Across Europe, reported HFRS notifications fluctuate substantially from year to year: between 2019 and 2023, annual rates ranged from 0.4 to 1.1 per 100,000 population, with 1,885 reported cases in 2023 and 4,046 cases in 2019, reflecting typical interannual variability driven by rodent population dynamics [7]. In 2023, Finland and Germany together accounted for 60.5% of European reports, and endemic activity is also well documented in Sweden, Belgium, Norway, France, Austria and Slovenia [7,8]. In Asia, China carries the heaviest burden globally, with national surveillance consistently recording tens of thousands of HFRS cases each year, estimated at roughly 20,000–50,000 annually in recent decades [9,10]. South Korea also reports sustained transmission, with several hundred cases reported annually and clear seasonal peaks [11]. By contrast, across North and South America combined, only a few hundred HCPS cases are typically reported each year [12]. It is likely that the true global incidence is higher, as asymptomatic or mild infections often go undetected [3].

Four *Orthohantavirus* species are recognised as the main causes of HFRS in Europe and Asia, each closely associated with a primary rodent reservoir (Table 1) [13]. In Europe, *Orthohantavirus puumalaense* (Puumala virus, PUUV) accounts for the majority of cases, typically causing the milder disease. *Orthohantavirus dobravaense* (Dobrava virus, DOBV) occurs mainly in the Balkans and parts of central and eastern Europe, where it is associated with more severe disease but fewer cases overall [14]. In Asia, *Orthohantavirus hantanense* (Hantaan virus, HTNV) is an important cause of severe disease, particularly in rural areas, whereas *Orthohantavirus seoulense* (Seoul virus, SEOV) is associated with moderate disease and has a wider distribution, maintained in commensal rats in both urban and peri-urban settings [15]. These four viruses represent the principal human pathogens, although other related viruses within the same species complexes have also been shown to infect humans and are summarised in Table 1. In addition, a fifth species, *Orthohantavirus tulaense* (Tula virus, TULV), has occasionally been reported in human infection, but cases are rare and typically mild, and its pathogenic significance remains uncertain [5].

**Table 1:** ICTV-recognised *Orthohantavirus* species causing HFRS in Europe and Asia.

ICTV-recognised species	Representative viruses	Geographical distribution	Primary rodent reservoir(s)
*Orthohantavirus dobravaense*	Dobrava virus; Sochi virus; Kurkino virus; Saaremaa virus	Balkans and southeast Europe; also central/eastern Europe	*Apodemus flavicollis* (yellow-necked mouse), *A. agrarius* (striped field mouse), *A. ponticus* (Black Sea field mouse)
*Orthohantavirus hantanense*	Hantaan virus; Amur virus; Soochong virus	China, South Korea, Far East Russia	*Apodemus agrarius* (striped field mouse)
*Orthohantavirus puumalaense*	Puumala virus; Muju virus; Hokkaido virus	Central and Northern Europe, Balkans, Western Russia	*Myodes glareolus* (bank vole)
*Orthohantavirus seoulense*	Seoul virus; Gou virus	Worldwide	*Rattus norvegicus* (brown rat), *R. rattus* (black rat)
*Orthohantavirus tulaense*	Tula virus	Europe and Russia	*Microtus arvalis* (common vole)

Representative viruses are named viral lineages within the ICTV-recognised species complexes. Geographical distribution and primary rodent reservoirs are also shown. (ICTV = International Committee on Taxonomy of Viruses; HFRS = haemorrhagic fever with renal syndrome) [1,3].

The emergence of HFRS in humans is closely tied to rodent populations, which fluctuate with climate and human activity [12]. An increase in the frequency and magnitude of HFRS epidemics in Europe has been partially attributed to the warming climate and increased land-use [16,17]. The epidemic potential of HFRS could increase further with a warming climate, possibly leading to more frequent outbreaks in endemic areas, and emergence of cases in new regions.

Eurasian hantaviruses infect microvascular endothelial cells and can disseminate to infect endothelial cells in most major organs. There is, however, prominent involvement of the renal microvasculature, particularly glomerular and tubular endothelial cells, contributing to the characteristic renal manifestations of disease [18]. Infected endothelial cells exhibit features of endothelial cell activation, with increased microvascular permeability and leakage of intravascular fluid into the extracellular compartment. Hantaviruses also trigger a vigorous immune response that contributes to pathological degrees of intravascular fluid leakage [19,20]. Varying degrees of acute renal failure occur secondary to this pathological process.

Acute thrombocytopenia is another central pathogenic feature in HFRS, with evidence of increased platelet turnover, hypothesised to be secondary to widespread adherence of circulating platelets to infected endothelium [21]. In keeping with this, bone marrow shows evidence of increased production with elevated thrombopoietin levels found in some studies [22,23]. Infection of the endothelium causing endothelial cell activation will result in a localised prothrombotic state. This, however, does not explain the bleeding tendency seen [24]. Some studies have demonstrated evidence of platelet dysfunction, but whether this is important in the development of haemorrhagic manifestations is poorly understood [21,23,25].

After an incubation period of ten days to four weeks, there is an abrupt onset of fever, typically associated with headache, myalgia, back pain and vomiting [3]. HFRS is classically described as occurring in five phases: febrile, hypotensive, oliguric, polyuric, and convalescent. Haemorrhagic manifestations typically occur during the hypotensive and oliguric phases of infection. These range from minor bleeding from small blood vessels in the skin, eyes and mouth, to major haemorrhage in the gastrointestinal tract, urinary tract and the brain [26]. HFRS can range from subclinical seroconversion to rapidly progressive and fatal. PUUV infection, known as nephropathia epidemica (NE) in Europe, is generally mild with a low case fatality rate (CFR), typically <0.1%, although estimates from Sweden suggest rates up to 0.4% [12,27]. Mild haemorrhagic symptoms are observed in around one third of NE cases [3]. In contrast, DOBV typically leads to a severe disease course in Europe with more significant bleeding, haemodynamic instability and renal failure, with case fatality rates estimated to be around 10% [28]. HTNV results in similarly severe disease in Asia with CFR estimates between 5 – 15% [3]. SEOV causes moderate disease severity in Asia and a CFR of <1 – 2% [28]. Nevertheless, cases of PUUV infection can be fulminant and fatal and DOBV or HTNV can result in mild disease.

Treatment of HFRS is entirely supportive. Haemodialysis use in severe renal failure, and transfusion of blood products in patients with severe thrombocytopenia or bleeding may be required. No licensed therapeutic agents currently exist for use in HFRS [3]. Diagnosis is dependent on a combination of clinico-epidemiological features and laboratory tests. Enzyme-linked immunosorbent assay (ELISA), indirect immunofluorescence assay (IFA) and reverse transcription polymerase chain reaction (RT-PCR) are widely used methods [3]. {Vial, 2023 #138}{Vial, 2023 #138}{Vial, 2023 #138}{Vial, 2023 #138}{Avsic-Zupanc, 2019 #103}{Avsic-Zupanc, 2019 #103}

### Haemostasis in HFRS

Microvascular endothelial dysfunction and/or activation induced by hantavirus infection is a cardinal pathological feature in HFRS and is likely a critical component in the prothrombotic change [24]. Separately, infected microvasculature may be predisposed to rupture giving rise to minor skin and mucosal petechiae, which would be exacerbated by thrombocytopenia [24]. Endothelial activation and injury in the early phases of hantavirus infection is a possible trigger for additional pathological coagulation disturbances as upregulation of tissue factor and activation of Factor XII have been described [29,30].

Indeed, it is well recognised that endothelial cell activation and injury expose subendothelial tissue factor, triggering the extrinsic coagulation pathway [24]. Hantavirus infection has also been shown to activate the intrinsic clotting pathway [30]. Both pathways converge at the activation of Factor X, which then converts prothrombin to thrombin, the key enzyme that transforms fibrinogen into insoluble fibrin strands forming the structural basis of the clot [31]. Widespread activation of the clotting cascade in hantavirus infection could cause a consumptive coagulopathy, indeed a disseminated intravascular coagulation (DIC), resulting in depletion of coagulation factors and diminished thrombin generation capacity. However, severe prolongation of PT and APTT reflecting significant loss of coagulation factors is rarely observed in HFRS, suggesting that this mechanism is unlikely to represent the primary pathway of haemostatic dysfunction [24]. PUUV-infected patients have also previously been shown to demonstrate enhanced fibrinolytic potential due to increased levels of tissue-type plasminogen activator (t-PA), which is sometimes a feature of endothelial cell activation [32]. It has been hypothesised that a combination of thrombocytopenia, diminished thrombin generation capacity, and hyperfibrinolysis shift the haemostatic balance towards hypocoagulability and haemorrhagic tendency [24].

Fully understanding the mechanisms through which hypocoagulability occurs in HFRS would provide important targets for novel therapeutic agents and alter clinical management strategies. Unfortunately, routine laboratory clotting parameters provide little insight into the causes of an individual patient’s haemostatic balance.

This systematic review aimed to characterise the laboratory clotting abnormalities and haemorrhagic manifestations observed in HFRS and explore differences between hantavirus species. These aims were achieved through a comprehensive synthesis of over 7,000 published cases, enabling detailed comparison between PUUV, DOBV, HTNV and SEOV. Notably, this is the first review to systematically quantify the frequency of specific haemorrhagic manifestations and compare them across different hantaviruses, highlighting under-recognised patterns of bleeding and key gaps in current reporting practices.

## Methods

### Database searches

This systematic review was conducted in accordance with Preferred Reporting Items for Systematic reviews and Meta-Analyses (PRISMA) guidelines [33]. Database searches were conducted on 12 December 2024 using MEDLINE, PubMed, CINAHL, Web of Science and Scopus. The search terms used are outlined in Table 2. No date restrictions were applied to the database searches. This review was registered on PROSPERO on 25^th^ November 2024 (CRD42024618760), and the protocol is publicly available at https://www.crd.york.ac.uk/PROSPERO/view/CRD42024618760.

**Table 2:** Systematic review database search terms.

Search Theme	Search Terms
Hantavirus	“hantavirus” **OR** “haemorrhagic fever with renal syndrome” **OR** “HFRS” **OR** “nephropathia epidemica” **OR** “Korean haemorrhagic fever”
AND	
Haemorrhagic manifestations	“bleeding” **OR** “coagulopathy” **OR** “coagulation” **OR** “clotting” **OR** “haematology” **OR** “haemostasis” **OR** “symptoms” **OR** “clinical” **OR** “platelet” **OR** “prothrombin time” **OR** “activated partial thromboplastin time” **OR** “international normalized ratio” **OR** “fibrinogen” **OR** “d dimer” **OR** “thromboelastography”
AND	
Study type	“case series” **OR** “observational study” **OR** “cohort study” **OR** “case-control study” **OR** “cross-sectional study”
AND	
Search Filters	Humans; English language

Three individual searches were initially conducted for each theme using the ‘OR’ Boolean command to separate the terms. Each individual search was also conducted using the listed filters. The results of these searches were then combined using the ‘AND’ Boolean command to produce the final database search results.

### Study selection

To be included in this review, studies had to meet all the following criteria: (1) observational study; (2) any human infection with hantaviruses causing HFRS; (3) laboratory confirmed infection using serological methods for specific IgM and/or IgG detection OR viral RNA detection using RT-PCR; (4) data reported on laboratory clotting parameters and/or haemorrhagic manifestations. Studies with any of the following were excluded: (1) suspected or confirmed infection with other pathogens; (2) reporting only severe or fatal cases of HFRS; (3) sample size <10 patients; (4) any study conducted at the same hospital site with an overlapping recruitment period to another included study; (5) studies with an interventional component without any baseline clinical data reported prior to intervention.

All references retrieved from the literature search were imported into Rayyan, a web-based tool designed to facilitate systematic reviews. Rayyan’s duplicate detection feature was employed to identify and remove duplicate records prior to screening. Two reviewers (MR and AH) independently screened titles and abstracts against the predefined inclusion criteria within Rayyan. For studies deemed potentially relevant, full-text articles were assessed independently by the same reviewers. Rayyan’s conflict detection functionality highlighted any discrepancies between reviewers’ decisions. These conflicts were initially resolved through discussion between the two reviewers; if consensus could not be reached, a third reviewer (TF) adjudicated the decisions.

### Data extraction

**Fig 1:**
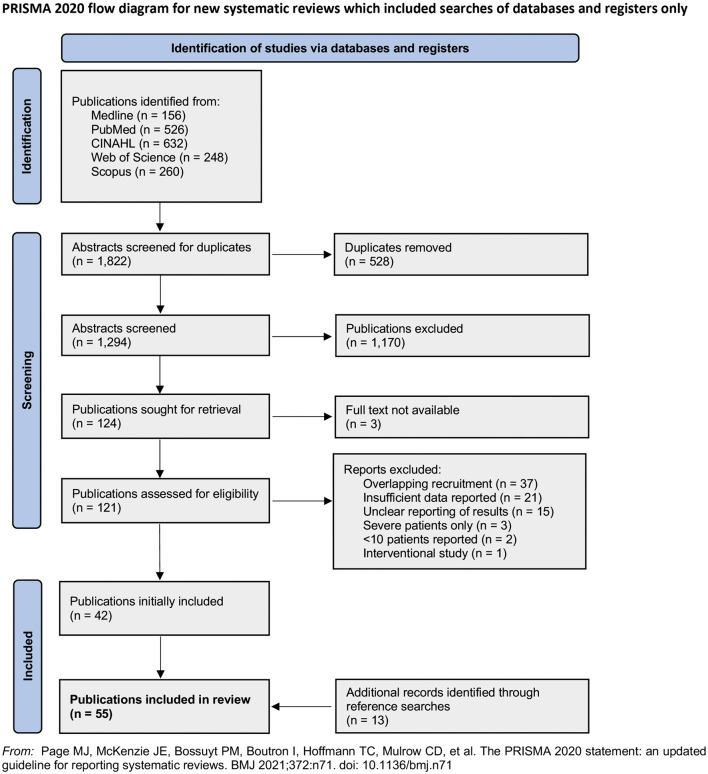
PRISMA flow diagram. Flowchart outlines the process from database searches through to final study inclusion. “n=” denotes the number of publications retrieved, excluded or included at each stage.

The process through which publications were identified, screened and included is depicted in Fig 1. A total of 55 studies were finally included in this review. Each publication was read in-depth. Demographic, laboratory and clinical data were extracted and collated in an excel spreadsheet. The laboratory parameters of interest were: platelet count, prothrombin time (PT), activated partial thromboplastin time (APTT), aspartate aminotransferase (AST), alanine aminotransferase (ALT), fibrinogen, haemoglobin, haematocrit, and d-dimer.

Data extraction was primarily conducted by MR using a standardised Excel spreadsheet and AH independently verified the accuracy of the extracted data. Any discrepancies identified were first discussed between the two reviewers to determine if they resulted from omissions or transcription errors and were corrected accordingly. If disagreements persisted, TF acted as a third reviewer to make a final decision.

The following data were extracted from each included study:

- Study details: study design, sample size, recruitment period, and location (country, city, hospital(s))
- Demographic details: age and gender of participants
- Causative hantavirus: specific virus identified as the cause
- Laboratory values: as specified above
- Haemorrhagic manifestations: number observed, site, and severity
- Clinical outcome: survival versus death

Haemorrhagic manifestations refer to clinically reported bleeding events described in the included studies (e.g., petechiae, mucosal bleeding, haematuria, gastrointestinal bleeding, pulmonary haemorrhage, intracranial haemorrhage). Microscopic haematuria refers to blood in the urine detectable only on laboratory testing (e.g., urine microscopy or dipstick), whereas macroscopic haematuria refers to visibly blood-stained urine. Patients with laboratory abnormalities alone were not recorded as having haemorrhagic manifestations unless clinically evident or radiologically confirmed bleeding was documented.

Instances of missing data were recorded as such during the data extraction process. These cases were initially discussed between MR and AH to assess their potential impact on the outcomes of the review. As the review progressed, it became evident that a meta-analysis would not be feasible due to the heterogeneity of reporting and the frequent absence of key data across many studies. Following consultation with the third reviewer (TF), it was agreed that rather than attempting to impute or exclude studies with missing data, all available data would be presented as reported, and instances of missing data would be transparently noted in the results.

### Quality assessment

The methodological quality and risk of bias (RoB) of each included study were systematically evaluated to ensure a robust assessment of internal validity, adhering to PRISMA guidelines for systematic reviews. Study designs were classified through detailed examination of the reported methodology and statistical approaches, following standardised definitions of study designs as outlined by Grimes and Schulz [34]. Studies were categorised as cohort studies only if they employed analytical statistics (e.g., relative risks, hazard ratios, or regression analyses) to compare outcomes between groups, indicating an analytical hypothesis. Studies with two groups that relied solely on descriptive statistics (e.g., means, percentages) to report clinical or laboratory features of HFRS were classified as comparative case series, reflecting their lack of analytical comparison [35].

The aim of this review was to collate clinical and laboratory data on HFRS patients, with all included studies deemed to have sufficient data for this purpose during the screening process. During data extraction, it was determined that calculating measures of effect or establishing causal links was not feasible. Consequently, the review focused on describing clinical and laboratory data as a whole, and the quality of studies did not preclude the extraction of available data from any study.

Quality assessment was conducted using the Joanna Briggs Institute (JBI) suite of critical appraisal tools, selected based on the identified study design [36–40]. For one interventional cohort study with a before-after design and no control group, the National Institutes of Health (NIH) Quality Assessment Tool for Before-After (Pre-Post) Studies With No Control Group was used [41]. Each tool consists of a checklist with questions addressing key bias domains, including selection, confounding, measurement, and statistical validity. Responses to each question were recorded as “Yes,” “No,” “Unclear,” or “Not Applicable" (NA).

To quantify methodological quality, a scoring system was used, tallying the number of “Yes” responses relative to the total applicable items in each checklist. Studies were assigned a quality grade based on this score: Grade 3 (highest quality, > 70% “Yes”), Grade 2 (50–70% “Yes”), and Grade 1 (lowest quality, < 50% “Yes”) [42,43]. It is important to note that the quality grade reflects the study’s adherence to the expected standards for its specific design. For example, a case series with a Grade 3 quality rating meets high standards for case series reporting but remains inherently more prone to bias than a well-conducted cohort study due to differences in study design hierarchy. Thus, a cohort study with a Grade 2 quality rating may still provide more robust evidence than a case series with a Grade 3 rating.

Two independent reviewers (MR and AH) conducted the quality assessments at the outcome level to minimise bias and ensure consistency. Discrepancies were resolved through discussion, with a third reviewer (TF) consulted when consensus could not be reached.

### Data synthesis

A formal meta-analysis of laboratory clotting parameters was not performed due to substantial clinical and methodological heterogeneity across the included studies. In addition to inconsistent reporting of central tendency measures and the absence of raw datasets, there were important differences in study populations, infecting hantavirus species, timing of laboratory measurements relative to disease phase, and definitions of haemorrhagic manifestations. Studies included patients infected with different hantavirus strains which are associated with varying clinical severity and haemostatic abnormalities. Furthermore, laboratory parameters were measured at different stages of illness across studies, limiting comparability between reported values. Definitions and reporting of haemorrhagic manifestations were also inconsistent, with some studies reporting minor mucocutaneous bleeding while others reported only clinically significant haemorrhage. These sources of heterogeneity, together with inconsistent reporting of laboratory parameters and lack of variance measures, precluded reliable quantitative pooling of data, including subgroup meta-analyses for commonly reported coagulation parameters.

Transformation of median data to mean data to calculate heterogeneity statistics was attempted but determined to introduce too much potential error into the sample. Instead, weighted averages were calculated using the following formula where *M* represents median or mean [44]:


$$\begin{array}{c} Weighted\;Average\;M=\;\frac{\Sigma (M\;\times \;Sample\;Size)}{\Sigma Sample\;Size} \end{array}$$

In this method, median or mean values from different studies are averaged, but weighted according to the sample size they are derived from. Due to a general lack of reporting on whether data were normally distributed or not, appropriate statistical tests could not be reliably selected to assess for differences in weighted averages. As such, p-values are not reported for these comparisons. Bubble plots were used to visualise the individual median and mean values reported for the various laboratory parameters. Haemorrhagic manifestations were reported as frequencies and percentages. Weighted average laboratory parameters and haemorrhagic manifestation frequencies are reported for all patients with available data as well as stratified by causative hantavirus. Several studies defined cohorts of severe cases in comparison to non-severe cases, including patients who died, those with severe renal failure, or according to severity scoring systems [45–47]. These cohorts of severe cases have been highlighted in the data reporting. Weighted average data for adults and children are considered separately but visualised together in bubble plots. Graphs were created using GraphPad Prism version 10.3.0 for Mac (GraphPad Software, San Diego, California, USA).

### Protocol amendments following registration

Following registration of this review on PROSPERO (CRD42024618760), several minor amendments were made to the protocol to improve the clarity and completeness of the final review. The review title was refined from “Characterising the loss of haemostasis in haemorrhagic fever with renal syndrome (HFRS): A systematic review” to “Bleeding in haemorrhagic fever with renal syndrome: A systematic review characterising the loss of haemostasis in hantavirus infections” to better reflect the review’s focus. The database search strategy was amended to replace the Cochrane Library with Scopus, as the review focused on observational, non-interventional study designs for which Cochrane was unlikely to yield relevant results. In addition, an extra search filter restricting results to human studies was applied alongside the English-language limitation to enhance relevance.

The use of blood products, listed as an outcome in the original protocol, was not reported due to insufficient data across included studies. During the screening process, the software Rayyan was used to facilitate abstract screening and manage inclusion/exclusion records, which was not specified in the original protocol but introduced to enhance transparency and reproducibility.

Regarding subgroup analyses, the planned analysis by study design was not undertaken due to limited comparative value between methodologies. Instead, a subgroup comparison of adult versus paediatric patients was added, as this was considered more clinically relevant. These amendments did not alter the overall objectives or analytical framework of the review but were made to strengthen methodological rigour and the interpretability of results.

## Results

### Literature search and quality assessment

Database searches yielded a total of 1,822 publications. After exclusion of duplicates and studies that did not meet screening criteria, 121 remained for in-depth review. After applying inclusion and exclusion criteria, a final total of 55 publications were included, with a total of 7,950 laboratory-confirmed cases of HFRS. S1 Table summarises the details each of the included studies.

Of the 55 studies included in this systematic review, study designs were classified as follows: 35 cohort studies, 14 case series, 4 case-control studies, 1 diagnostic test accuracy study, and 1 interventional cohort study without a control group. Quality assessment revealed that 31 studies (56.4%) were classified as grade 3 (highest quality), 23 studies (41.8%) as grade 2, and 1 study as grade 1 (lowest quality). Detailed study characteristics, including design and quality grading, are summarised in S1 Table.

The overall methodological quality of the included studies varied across designs. Cohort studies generally demonstrated the highest quality, with most using clearly defined inclusion criteria, standardised diagnostic methods, and reliable measurement of exposures and outcomes. These studies frequently reported demographic and clinical data comprehensively and used appropriate statistical analyses. However, some were limited by incomplete follow-up and potential selection bias where recruitment was not clearly consecutive.

Case series, which formed a substantial proportion of the evidence base, typically provided detailed descriptions of clinical features and laboratory findings, often supported by reliable diagnostic confirmation. Nevertheless, their methodological rigour was reduced by small sample sizes, retrospective data collection, and limited information on recruitment processes, raising concerns about representativeness.

Case-control studies performed well in terms of diagnostic confirmation and clear reporting of clinical data, but most lacked sample size justification and did not always provide sufficient detail on matching procedures or strategies to minimise selection bias.

The single interventional study used validated measurement methods and reported outcomes consistently but was constrained by a small sample size and lacked a control group, limiting interpretation. Similarly, the diagnostic test accuracy study applied appropriate reference standards and reported results transparently, but potential bias arose from limited information on participant selection and blinding.

Across all study designs, common strengths included the widespread use of standardised diagnostic methods and detailed clinical reporting. However, frequent limitations involved small sample sizes, retrospective data collection, incomplete reporting of recruitment strategies, and restricted representativeness, all of which should be considered when interpreting findings.

To better understand how study quality contributed to the evidence base for each outcome, the proportion of patients contributing data from studies of different quality grades was examined. Across the overall cohort of 7,950 laboratory-confirmed HFRS cases, 66.2% of patients were derived from grade 3 studies, 33.3% from grade 2 studies, and 0.5% from a grade 1 study.

For most laboratory parameters, the majority of available data originated from higher-quality studies. Platelet count data were predominantly derived from grade 3 studies (75.4%), as were haemoglobin concentration measurements (78.6%). Similarly, prothrombin time, activated partial thromboplastin time, and fibrinogen measurements were largely contributed by grade 3 studies (80.4%, 76.0%, and 81.8%, respectively). D-dimer measurements showed the highest proportion of data originating from grade 3 studies (93.2%). Liver enzyme measurements were also largely derived from higher-quality studies, with 73.6% of aspartate aminotransferase (AST) data and 72.5% of alanine aminotransferase (ALT) data originating from grade 3 studies.

For clinical outcomes, survival data were predominantly derived from grade 3 studies (69.9%), with a smaller proportion from grade 2 studies (29.6%) and minimal contribution from grade 1 studies (0.5%). Reporting of haemorrhagic manifestations was more evenly distributed between grade 3 studies (47.8%) and grade 2 studies (51.3%), with a small contribution from grade 1 studies (0.9%). Data for paediatric patients were derived almost entirely from higher-quality studies, with 94.1% originating from grade 3 studies and the remainder from grade 2 studies.

### Demographic and epidemiological data

Cases were reported from a total of 16 countries, predominantly from China (Table 3). 4,046 cases were caused by HTNV, 2,545 by PUUV, 93 by DOBV and 81 by SEOV. In 1,185 cases the causative hantavirus was not reported despite tests confirming acute hantavirus infection. Amongst adult patients, the weighted average median age of 3,510 patients was 43.2 years, and the weighted average mean age of 4,037 patients was 40.5 years (Table 4). Weighted average median and mean ages were similar when stratifying by hantavirus species amongst adult patients. 5,801 adult patients were male (76.6%) and 1,775 were female (23.4%) (Table 5). The weighted average median age of 105 paediatric cases was 12.1 years, and this data was derived entirely from PUUV-induced HFRS (Table 4). 260 paediatric cases were male (69.5%) and 114 were female (30.5%) (Table 5).

**Table 3:** Number of publications and HFRS cases reported from each country.

Country	Number of publications	Number of patients	Hantavirus
China	16	4,141	HTNV, SEOV
Sweden	4	1,169	PUUV
Germany	5	588	PUUV
South Korea	8	445	HTNV, SEOV
France	1	387	PUUV
Finland	3	246	PUUV
Russia	1	228	PUUV
Denmark	1	184	PUUV
Slovenia	4	170	DOBV, HTNV, PUUV
Austria	3	124	PUUV
Turkiye	3	114	DOBV, PUUV
Bosnia & Herzegovina	1	40	PUUV
Albania	1	33	DOBV
Belgium	2	29	PUUV
Croatia	1	29	DOBV, PUUV
Bulgaria	1	23	DOBV, PUUV

Causative hantaviruses reported from each country are also shown. Table is ordered according to the number of patients**.** (DOBV = Dobrava virus; HTNV = Hantaan virus; PUUV = Puumala virus; SEOV = Seoul virus).

**Table 4:** Weighted average median and mean age of adult and paediatric patients.

	All Patients	PUUV	DOBV	HTNV	SEOV
**Age (years) – adults**					
Weighted average median	**43.2**	**44.8**	**NR**	**42.3**	**NR**
Sample size	3,510	1,084	NR	2,244	NR
Number of publications	20	10	NR	7	NR
Number of patient cohorts	25	11	NR	9	NR
Weighted average mean	**40.5**	**39.7**	**40.3**	**43.6**	**37.6**
Sample size	4,037	1,234	49	1,533	81
Number of publications	28	9	2	9	2
Number of patient cohorts	41	10	2	17	2
**Age (years) – children**					
Weighted average median	**12.1**	**12.1**	**NR**	**NR**	**NR**
Sample size	105	105	NR	NR	NR
Number of publications	4	4	NR	NR	NR
Number of patient cohorts	4	4	NR	NR	NR
Weighted average mean	**NR**	**NR**	**NR**	**NR**	**NR**
Sample size	NR	NR	NR	NR	NR
Number of publications	NR	NR	NR	NR	NR
Number of patient cohorts	NR	NR	NR	NR	NR

The sample sizes, number of publications and patient cohorts that the weighted averages are derived from are shown. Values are demonstrated for all included HFRS patients with available data, as well as stratified by causative hantavirus. ‘NR’ indicates patient groups where no data were reported. (DOBV = Dobrava virus; HTNV = Hantaan virus; NR = not reported; PUUV = Puumala virus; SEOV = Seoul virus).

**Table 5:** Gender distribution of adult and paediatric patients.

	All Patients	PUUV	DOBV	HTNV	SEOV
**Gender – adults**					
Male	**5,801 (76.6%)**	**1,654 (70.7%)**	**53 (93.0%)**	**2,999 (79.4%)**	**56 (69.1%)**
Female	**1,775 (23.4%)**	**685 (29.3%)**	**4 (7.0%)**	**778 (20.6%)**	**25 (30.9%)**
Sample size	7,576	2,339	57	3,777	81
Number of publications	49	20	3	16	2
Number of patient cohorts	72	22	3	26	2
**Gender – children**					
Male	**260 (69.5%)**	**68 (64.8%)**	**NR**	**192 (71.4%)**	**NR**
Female	**114 (30.5%)**	**37 (35.2%)**	NR	**77 (28.6%)**	NR
Sample size	374	105	NR	269	NR
Number of publications	6	4	NR	2	NR
Number of patient cohorts	6	4	NR	2	NR

Data are reported as frequency (percentage). The sample sizes, number of publications and patient cohorts that the data are derived from are shown. Values are demonstrated for all included HFRS patients with available data, as well as stratified by causative hantavirus. (DOBV = Dobrava virus; HTNV = Hantaan virus; NR = not reported; PUUV = Puumala virus; SEOV = Seoul virus).

The majority of patients included in the reviewed studies were hospitalised. Among HTNV cohorts, 3,896 of 4,046 patients (96.3%) were explicitly reported as hospital inpatients, with the remaining cases derived from studies where hospitalisation could reasonably be inferred from the study design and recruitment process. Similarly, all SEOV patients were derived from hospital-based cohorts, and 77 of 93 DOBV patients (82.8%) were explicitly reported as hospital inpatients, with the remaining cases derived from studies where hospitalisation could reasonably be inferred. Among PUUV patients, 2,101 of 2,545 (82.6%) were explicitly reported as hospitalised inpatients, while 158 patients (6.2%) were reported as outpatients in studies that included both inpatient and outpatient populations. The remaining PUUV cases were derived from studies where hospitalisation was likely but not explicitly stated or where admission status could not be determined.

### Laboratory clotting parameters

#### Adult patients.

Average (median or mean) platelet counts were reported from 46 publications, from a total sample size of 6,562 patients. Average PT data was available for 3,026 patients across 10 studies, APTT for 3,180 patients across 12 studies, and fibrinogen for 2,884 patients across 6 studies. Average D-dimer values were reported from only 5 studies, from 2,153 patients. Data on PT, APTT, fibrinogen, and D-dimer were mainly from HTNV cases. AST data was available for 5,095 patients from 31 studies, and ALT for 5,017 patients from 29 studies.

**Fig 2:**
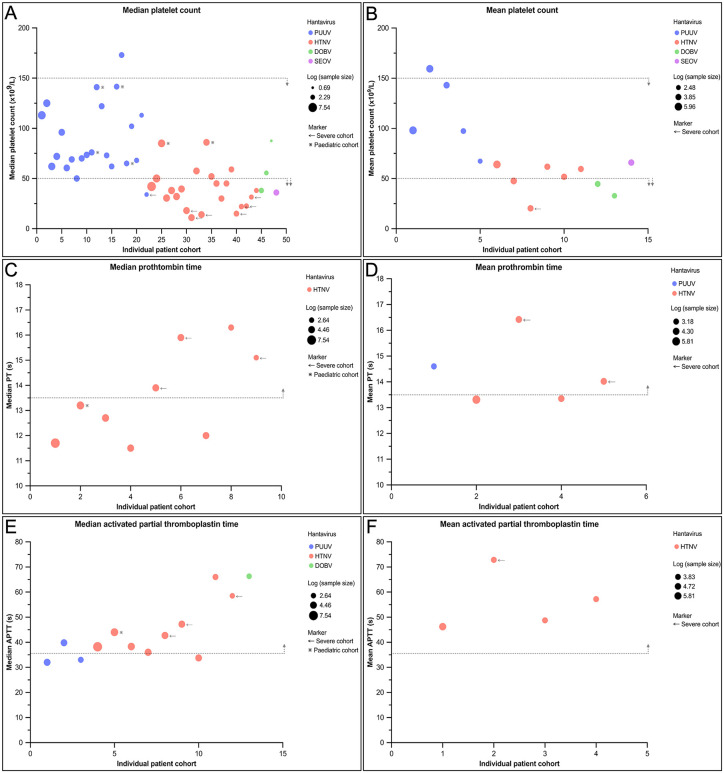
Bubble plots of platelet count and coagulation times by individual study cohort. Panel A shows median platelet counts and panel B shows mean platelet counts. Panel C shows median prothrombin times (PT) and panel D shows mean prothrombin times. Panel E shows median activated partial thromboplastin times (APTT) and panel F shows mean activated partial thromboplastin times. Each point represents a median or mean value from an individual study cohort. The size of each point represents the sample size on a natural logarithmic scale. Log(sample size) of 7.54 corresponds to a raw sample size of 1,873, and Log(sample size) of 0.69 corresponds to a raw sample size of 2. Bubble colour denotes causative hantavirus. Points denoted with a ‘←’ symbol represent severe cohorts, and points denoted with a ‘⋇’ symbol represent paediatric cohorts. Grey dotted lines illustrate reference ranges and thresholds of clinical importance, with arrows indicating the direction of abnormality. (APTT = activated partial thromboplastin time; DOBV = Dobrava virus; HTNV = Hantaan virus; PT = prothrombin time; PUUV = Puumala virus; SEOV = Seoul virus).

**Fig 3:**
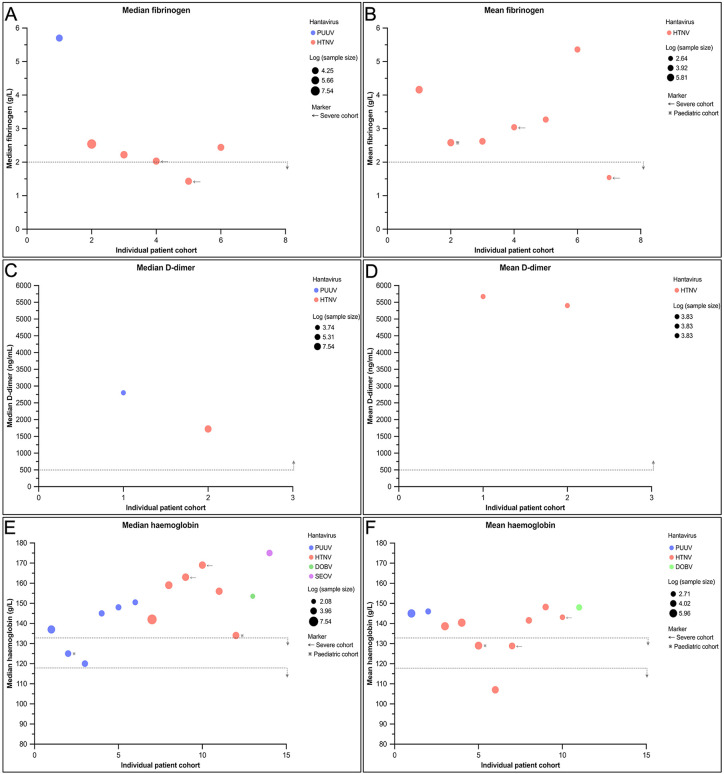
Bubble plots of fibrinogen, D-dimer, and haemoglobin concentrations by individual study cohort. Panel A shows median fibrinogen concentrations and panel B shows mean fibrinogen concentrations. Panel C shows median D-dimer concentrations and panel D shows mean D-dimer concentrations. Panel E shows median haemoglobin concentrations and panel F shows mean haemoglobin concentrations. Each point represents a median or mean value from an individual study cohort. The size of each point represents the sample size on a natural logarithmic scale. Log(sample size) of 7.54 corresponds to a raw sample size of 1,873, and Log(sample size) of 0.69 corresponds to a raw sample size of 2. Bubble colour denotes causative hantavirus. Points denoted with a ‘←’ symbol represent severe cohorts, and points denoted with a ‘⋇’ symbol represent paediatric cohorts. Grey dotted lines illustrate reference ranges and thresholds of clinical importance, with arrows indicating the direction of abnormality. (DOBV = Dobrava virus; HTNV = Hantaan virus; PUUV = Puumala virus; SEOV = Seoul virus).

**Fig 4:**
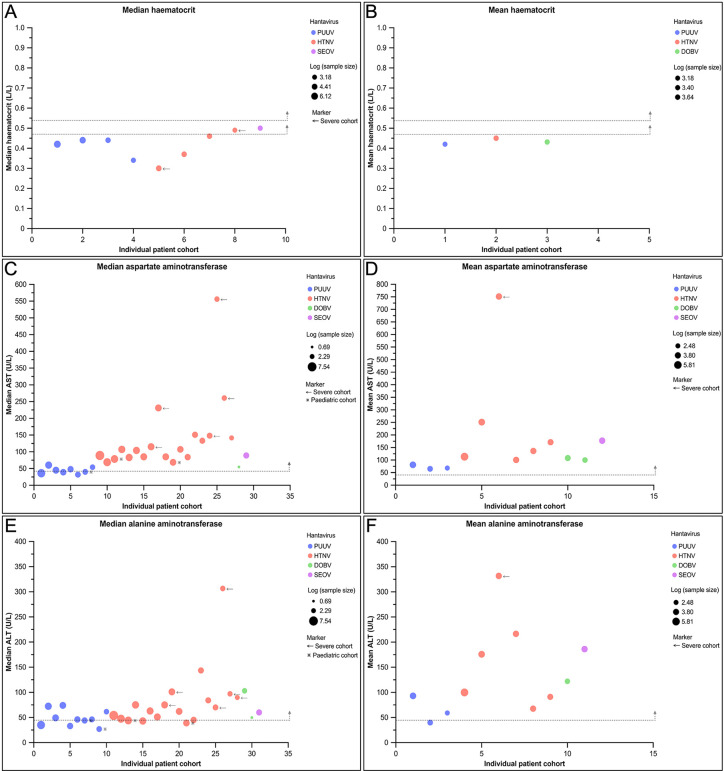
Bubble plots of haematocrit and liver enzyme concentrations by individual study cohort. Panel A shows median haematocrit levels and panel B shows mean haematocrit levels. Panel C shows median aspartate aminotransferase (AST) concentrations and panel D shows mean aspartate aminotransferase concentrations. Panel E shows median alanine aminotransferase (ALT) concentrations and panel F shows mean alanine aminotransferase concentrations. Each point represents a median or mean value from an individual study cohort. The size of each point represents the sample size on a natural logarithmic scale. Log(sample size) of 7.54 corresponds to a raw sample size of 1,873, and Log(sample size) of 0.69 corresponds to a raw sample size of 2. Bubble colour denotes causative hantavirus. Points denoted with a ‘←’ symbol represent severe cohorts, and points denoted with a ‘⋇’ symbol represent paediatric cohorts. Grey dotted lines illustrate reference ranges and thresholds of clinical importance, with arrows indicating the direction of abnormality. (ALT = alanine aminotransferase; AST = aspartate aminotransferase; DOBV = Dobrava virus; HTNV = Hantaan virus; PUUV = Puumala virus; SEOV = Seoul virus).

Detailed data on the weighted average median and mean lab parameters of adult patients are contained in S2 Table, and the values discussed here are for adult patients only. Bubble plots in Figs 2–4 depict the distribution of observed median or mean values from various patient cohorts, including paediatric cohorts. Thrombocytopenia was common, with a weighted average median platelet count of 56.2 x 10⁹/L overall. Thrombocytopenia was more severe in HTNV cases compared to PUUV, with a weighted average median platelet count of 39.9 x 10⁹/L vs 94.5 x 10⁹/L, respectively. Severe cases showed more marked thrombocytopenia compared to non-severe cases (16.3 x 10⁹/L vs 58.7 x 10⁹/L) (Fig 2). The weighted average median PT across all cohorts was 12.1 s. PT prolongation was noted in severe cases (14.9 s) compared to non-severe (11.8 s) (Fig 2). APTT was overall slightly prolonged (weighted average median 38.8 s), with more marked prolongation in severe cases compared to non-severe (weighted average median 45.9 s vs 38.3 s) (Fig 2).

Fibrinogen levels were generally normal (weighted average median 2.57 g/L), though mild hypofibrinogenemia was noted in severe HTNV cases (weighted average median 1.75 g/L) (Fig 3). Average D-dimer values were consistently elevated in the few studies that reported it, with a weighted average median of 1,810.3 ng/mL (Fig 3).

Average haemoglobin and haematocrit levels were generally within normal limits (Figs 3 and 4 respectively). Liver function showed moderate derangements, with a weighted average median AST of 85.7 U/L and ALT of 56.1 U/L across all patients. Weighted average median AST values were higher in HTNV patients (97.0 U/L) compared to PUUV (41.3 U/L) (Fig 4), while differences in weighted average median ALT were less pronounced (58.7 U/L for HTNV vs 45.4 U/L for PUUV) (Fig 4). Both AST and ALT were higher in cohorts of severe HFRS caused by HTNV (Fig 4).

#### Paediatric patients.

The paediatric cohort of patients was significantly smaller than the adult cohort, but there was still a general paucity of reported data on laboratory clotting parameters from these patients. There was no laboratory clotting data related to DOBV or SEOV as there were no paediatric cases of HFRS caused by these hantaviruses included in this review. Full details of weighted average median and mean values are contained in S3 Table. Paediatric cohorts are also denoted on the bubble plots in Figs 2–4 alongside adult cohorts.

Median platelet counts were reported for 105 PUUV cases from four publications, and for 269 HTNV cases from two publications (Fig 2). The weighted average median platelet count was lower for HTNV compared with PUUV (85.2 x 10⁹/L vs 107.5 x 10⁹/L). A median PT of 13.2 s and a median APTT of 44.0 s were reported from one cohort of 206 HTNV cases (Fig 2). The same publication reported a mean fibrinogen concentration of 2.58 g/L (Fig 3). Median haemoglobin values were reported from one cohort of 32 PUUV cases (125.0 g/L) and one cohort of 63 HTNV cases (134.0 g/L) (Fig 3). A mean haemoglobin concentration of 129.0 g/L was also reported from one cohort of 206 HTNV cases (Fig 3). The weighted average median AST was 76.2 U/L for 269 HTNV cases, derived from two publications (Fig 4). One cohort of 22 paediatric PUUV cases reported a median AST of 40.0 U/L (Fig 4). With regards to ALT, a weighted average median value of 42.8 U/L was derived from 269 cases of HTNV, compared to a weighted average median ALT of 37.1 U/L from 54 PUUV cases (Fig 4). No data on D-dimer or haematocrit were reported for paediatric patients.

### Haemorrhagic manifestations

#### Adult patients.

Table 6 contains the frequency and percentages of various haemorrhagic manifestations stratified by causative hantavirus amongst adult patients. It is important to note that the sample sizes this data were derived from were significantly larger for PUUV and HTNV cases compared to SEOV and DOBV. Microscopic haematuria was a relatively common observation across all hantaviruses, present in 28.4% of SEOV cases, 26.7% of DOBV cases, 24.3% of PUUV cases, and 15.8% of HTNV cases. Macroscopic haematuria was reported more frequently in SEOV cases (19.8%), compared to HTNV (2.3%), PUUV (1.5%), and DOBV (0.0%).

**Table 6:** Haemorrhagic manifestations stratified by causative hantavirus in adult patients.

Bleeding site	PUUV n = 1,597		HTNV n = 1,265		DOBV n = 60		SEOV n = 81	
	Freq	%	Freq	%	Freq	%	Freq	%
Microscopic haematuria	388	24.3	200	15.8	16	26.7	23	28.4
Macroscopic haematuria	24	1.5	29	2.3	0	0.0	16	19.8
Petechiae of skin	136	8.5	90	7.1	0	0.0	20	24.7
Ecchymoses of skin	3	0.2	211	16.7	8	13.3	0	0.0
Bleeding from puncture sites	0	0.0	39	3.1	0	0.0	9	11.1
Epistaxis	24	1.5	26	2.1	2	3.3	8	9.9
Bleeding of oropharynx	6	0.4	156	12.3	3	5.0	10	12.3
Ocular bleeding	43	2.7	130	10.3	0	0.0	13	16.0
Gastrointestinal bleeding	39	2.4	93	7.4	4	6.7	18	22.2
Pulmonary haemorrhage	1	0.1	6	0.5	0	0.0	2	2.5
Intracranial haemorrhage	0	0.0	2	0.2	0	0.0	0	0.0
Other	25	1.6	52	4.1	10	16.7	14	17.3

The sample size number (n=) denotes the number of patients with available data on haemorrhagic manifestations included in this analysis. Frequencies of each manifestation are shown along with the associated percentage of the sample. (DOBV = Dobrava virus; HTNV = Hantaan virus; PUUV = Puumala virus; SEOV = Seoul virus).

Petechiae of the skin were observed in 24.7% of SEOV cases, compared to 8.5% of PUUV cases and 7.1% of HTNV cases. No petechiae were recorded for DOBV cases. Ecchymoses of the skin were most frequently observed amongst the HTNV (16.7%) and DOBV (13.3%) cohorts and were infrequent in the PUUV cohort (0.2%) and absent in the SEOV cohort. Epistaxis was recorded in all cohorts, most frequently in SEOV cases (9.9%) and infrequently across the other three hantaviruses. Bleeding of the oropharynx was recorded in 12.3% of both the HTNV and SEOV cohorts, which was more common than the DOBV (5.0%) and PUUV (0.4%) cohorts. Ocular bleeding (typically subconjunctival and conjunctival haemorrhages) was recorded in 16.0% of SEOV cases, 10.3% of HTNV cases, 2.7% of PUUV cases, and no DOBV cases.

With regards to more significant haemorrhagic manifestations, gastrointestinal bleeding was the most common site of bleeding. 22.2% of SEOV cases had evidence of gastrointestinal bleeding, compared to 7.4% of HTNV cases, 6.7% of DOBV cases and 2.4% of PUUV cases. Pulmonary and intracranial haemorrhages were rarely recorded across all hantaviruses (Table 6). Additional sites of both minor and major bleeding were recorded either in small numbers or could not be categorised due to ambiguity of reporting. These were recorded as ‘other’ haemorrhagic manifestations, and the specific breakdown of these is shown in S4 Table.

#### Paediatric patients.

No data on haemorrhagic manifestations were reported for any paediatric cases of HFRS caused by DOBV or SEOV. Data were available for small sample sizes of PUUV and HTNV cases and this is shown in Table 7. Microscopic haematuria was a very common observation, present in 71.4% of PUUV cases 66.7% of HTNV cases. Macroscopic haematuria was also observed in 60.3% of HTNV cases, but none of the PUUV cases. No paediatric cases of PUUV or HTNV had any recorded instances of skin petechiae, ecchymoses or bleeding from puncture sites. Epistaxis and oropharyngeal bleeding were observed amongst 5.7% and 1.9% of PUUV cases, respectively, but no HTNV cases. Ocular bleeding was more frequently observed amongst the HTNV cohort (55.6%) compared to the PUUV cohort (1.0%).

**Table 7:** Haemorrhagic manifestations stratified by causative hantavirus in paediatric patients.

Bleeding site	PUUV n = 105		HTNV n = 63		DOBV n = 0		SEOV n = 0	
	Freq	%	Freq	%	Freq	%	Freq	%
Microscopic haematuria	75	71.4	42	66.7	NR	NR	NR	NR
Macroscopic haematuria	0	0.0	38	60.3	NR	NR	NR	NR
Petechiae of skin	0	0.0	0	0.0	NR	NR	NR	NR
Ecchymoses of skin	0	0.0	0	0.0	NR	NR	NR	NR
Bleeding from puncture sites	0	0.0	0	0.0	NR	NR	NR	NR
Epistaxis	6	5.7	0	0.0	NR	NR	NR	NR
Bleeding of oropharynx	2	1.9	0	0.0	NR	NR	NR	NR
Ocular bleeding	1	1.0	35	55.6	NR	NR	NR	NR
Gastrointestinal bleeding	3	2.9	6	9.5	NR	NR	NR	NR
Pulmonary haemorrhage	0	0.0	5	7.9	NR	NR	NR	NR
Intracranial haemorrhage	0	0.0	0	0.0	NR	NR	NR	NR
Other	4	3.8	0	0.0	NR	NR	NR	NR

The sample size number (n=) denotes the number of patients with available data on haemorrhagic manifestations included in this analysis. Frequencies of each manifestation are shown along with the associated percentage of the sample. ‘NR’ indicates patient groups where no data were reported. (DOBV = Dobrava virus; HTNV = Hantaan virus; PUUV = Puumala virus; SEOV = Seoul virus).

More significant haemorrhagic manifestations were uncommon in paediatric cases of PUUV infection. 2.9% of these cases had gastrointestinal bleeding recorded, and no instances of pulmonary haemorrhage. In contrast, amongst the HTNV cohort, 9.5% had evidence of gastrointestinal bleeding and 7.9% pulmonary bleeding. No paediatric cases of PUUV or HTNV had intracranial bleeding. Four patients within the PUUV cohort were recorded as having ‘other’ haemorrhagic manifestations. These related to two cases recorded as having ‘visible bleeding’ without any site specified, and two cases of abnormal vaginal bleeding.

### Clinical outcome

Studies that reported on survival and death were used to calculate case fatality rates (CFR) for each hantavirus (Table 8). The case fatality rate for adult cases of HFRS caused by PUUV, DOBV and HTNV were 0.1%, 10.6% and 6.0% respectively. No deaths were recorded amongst SEOV patients. For paediatric cases, the case fatality rate of HTNV cases was 2.6%. No paediatric cases of PUUV died. No data were reported for paediatric cases of DOBV or SEOV.

**Table 8:** Case fatality rate of different hantaviruses amongst adult and paediatric patients.

	PUUV	DOBV	HTNV	SEOV
Adults				
Survived	2337	76	3335	81
Died	3	9	213	0
Unknown	205	8	498	0
CFR	0.10%	10.60%	6.00%	0.00%
Children				
Survived	105	NR	262	NR
Died	0	NR	7	NR
Unknown	0	NR	0	NR
CFR	0.00%	NR	2.60%	NR

Unknown outcome represents patient cohorts where survival or death were not reported. Unknown outcomes were excluded from case fatality rate (CFR) calculations. ‘NR’ indicates patient groups where no data were reported. (DOBV = Dobrava virus; HTNV = Hantaan virus; NR = not reported; PUUV = Puumala virus; SEOV = Seoul virus).

## Discussion

This systematic review offers valuable insights into the coagulation disturbances and haemorrhagic manifestations associated with HFRS. A structured summary of the principal findings, data availability, study quality contribution, and key interpretative limitations is provided in S5 Table. The standard of reporting for laboratory values varied greatly and missing data were frequently encountered. Despite this, laboratory clotting data were still available for several thousand adult HFRS patients. Thrombocytopenia was a consistent finding across all study cohorts. The degree of thrombocytopenia was less severe in PUUV infections compared to other hantaviruses but varied significantly between groups of patients with the same causative hantavirus. This may be a result of confounding factors such as timing of blood samples or individual patient characteristics that are not possible to account for in this review. It does, however, add considerable weight to the suggestion that thrombocytopenia alone may not be the sole underlying cause for the development of haemorrhagic manifestations. The mechanisms underlying thrombocytopenia in HFRS are likely multifactorial. Experimental and clinical studies suggest that hantavirus infection is associated with activation of the vascular endothelium, which promotes platelet adhesion and aggregation within the microvasculature [18,25]. In addition, immune-mediated platelet destruction and increased platelet consumption have been proposed as contributing factors [18]. Platelet activation in response to endothelial dysfunction may therefore reduce circulating platelet counts while simultaneously altering platelet function, potentially contributing to bleeding manifestations even when thrombocytopenia is only moderate in severity [23].

Prolongation of PT was observed in a subset of HTNV cohorts with severe disease. APTT prolongation was frequently observed in both non-severe and severe cohorts of HTNV patients, and more pronounced in severe cases. Individual cohorts of PUUV and DOBV cohorts also demonstrated APTT prolongation. This potentially indicates an intrinsic clotting pathway disturbance, but as there was some degree of extrinsic pathway disturbance in more severe cases, there could be a common pathway defect or interference in fibrin formation due to high levels of D-dimers. Despite elevated D-dimer levels, hypofibrinogenemia was not frequently observed apart from two cohorts of severe HTNV patients, suggesting that as raised D-dimers are markers of inflammation as well as fibrinolytic activity, severe fibrinolytic activity may only have been present in those with severe HTNV. Koskela et al. have previously hypothesised that a mild consumptive coagulopathy may develop in PUUV-infected patients [24]. Taken together, this pattern of mild prolongation of coagulation times, elevated D-dimer levels, and generally preserved fibrinogen concentrations is more consistent with a limited or localised consumptive coagulopathy rather than severe systemic factor depletion [24]. Elevated D-dimer levels may reflect both activation of fibrinolysis and inflammatory processes accompanying endothelial injury. The preservation of fibrinogen levels in most cohorts suggests that widespread depletion of coagulation factors is uncommon, supporting the hypothesis that haemostatic disturbances in HFRS may arise from a combination of modest coagulation activation and endothelial-driven alterations in haemostasis rather than severe systemic consumption [24,48]. Significant hepatic impairment was not commonly observed, which contrasts with the liver injury seen with other bunyaviruses like CCHF [49]. Significant hepatic impairment, by limiting coagulation factor and thrombopoietin production, may contribute to clotting disturbance in severe cases but does not appear to play a major role in general.

A central feature of hantavirus pathogenesis is infection of vascular endothelial cells, which is associated with dysregulation of endothelial barrier function and increased vascular permeability [50,51]. Clinical studies in HFRS patients have demonstrated evidence of endothelial activation, including degradation of the endothelial glycocalyx and increased circulating adhesion molecules such as soluble ICAM-1, VCAM-1, and E-selectin during the acute phase of infection [52]. These processes may contribute to both thrombocytopenia and microvascular leakage, key hallmarks of HFRS. Endothelial dysfunction may therefore represent a plausible mechanism linking the observed laboratory abnormalities with the clinical manifestations of haemorrhage and capillary leakage observed in affected patients.

When considered alongside other viral haemorrhagic fevers, the haemostatic disturbances observed in HFRS appear to follow a somewhat distinct pattern. In infections such as Ebola virus disease and Crimean–Congo haemorrhagic fever, haemorrhagic cases are often associated with more pronounced derangements of coagulation pathways, including marked prolongation of clotting times and evidence of significant clotting factor depletion in the most severe cases, sometimes accompanied by hypofibrinogenaemia [53–56]. In contrast, the findings synthesised in this review suggest that HFRS is more commonly characterised by moderate thrombocytopenia and mild coagulation pathway disturbances rather than severe systemic depletion of coagulation factors.

It is important to consider that microscopic haematuria may be more of a reflection of glomerular dysfunction induced by hantavirus infection of the renal microvasculature as opposed to true haemorrhage. Still, microscopic haematuria was frequently observed across all four hantavirus groups, and very common in paediatric patients with PUUV and HTNV infection. Urine dipstick may therefore be a useful screening tool when considering a diagnosis of HFRS. There were notable differences in haemorrhagic manifestations between hantavirus types. Aside from minor petechiae of the skin, PUUV-infected patients had low rates of bleeding complications. In contrast, minor skin, mucosal, and gastrointestinal bleeding were more common in HTNV, DOBV, and SEOV cases. The most commonly reported forms of bleeding in this review were petechiae and ecchymoses of the skin, oropharyngeal bleeding, ocular bleeding and gastrointestinal bleeding. The data reported here suggest highest rates of bleeding in SEOV patients. Caution should be used when interpreting this, however, given the significantly smaller sample size of SEOV patients included in this review. The lack of individual patient data prevented subgroup analyses based on the degree of clotting parameter derangement. Similarly, the reporting of such data in the source publications typically did not report data according to haemorrhagic and non-haemorrhagic cohorts, limiting the ability to directly relate laboratory abnormalities to clinical bleeding outcomes. In addition, most studies did not present laboratory or clinical data stratified by sex, preventing assessment of potential sex-related differences in haemostatic abnormalities. These limitations highlight the need for future studies specifically designed to investigate haemostatic dysfunction in HFRS, with prospective collection of standardised laboratory parameters and systematic reporting of outcomes according to bleeding status and patient characteristics such as sex.

Only 374 cases of paediatric HFRS were captured by this review, equating to 4.7% of the entire sample size. This is in-keeping with epidemiological reports from Europe and Asia demonstrating that HFRS is significantly more common in adults than children [57,58]. Only PUUV and HTNV-related paediatric cases were represented, with no data available for DOBV or SEOV. Among the limited laboratory data, paediatric HTNV cases had lower weighted average median platelet counts than PUUV cases, and prolonged APTT was observed in one HTNV cohort, though PT and fibrinogen values remained within normal ranges. ALT and AST levels were generally mildly elevated, particularly in HTNV cases. Haemorrhagic manifestations were infrequently reported in children and were generally milder than in adults. Microscopic haematuria was common in both PUUV and HTNV cohorts, but more overt bleeding, such as gastrointestinal or pulmonary haemorrhage, was observed predominantly in HTNV cases. Notably, severe bleeding such as intracranial haemorrhage was absent across all paediatric cases. The paediatric case fatality rate was lower than that reported in adults for HTNV (2.6% vs 6.0%), and no deaths were recorded among paediatric PUUV cases. These findings support the observation that HFRS appears to be less severe in children than in adults [59–61].

The overall quality of the studies included in this review was mixed, with most studies rated as moderate to high quality, particularly the larger cohort studies by Hu et al., Settergren et al., and Latus et al., which provided robust methodology and comprehensive reporting of clinical and laboratory parameters [62–64]. Smaller, prospective studies of good methodological quality also contributed valuable insights, particularly where they included standardised diagnostic methods and detailed outcome reporting [65–68]. While these studies form a strong foundation, much of the remaining literature consists of retrospective observational studies and case series of variable quality, often constrained by small sample sizes, incomplete recruitment strategies, and heterogeneous reporting of outcomes. A further consideration is that methods of performing PT and APTT and measuring fibrinogen and D-dimer would vary between different sites.

An additional analysis examining the contribution of studies of different methodological quality to each outcome demonstrated that the majority of the synthesised data were derived from higher-quality studies. Across the overall cohort, two-thirds of patients originated from studies graded as the highest quality. For most laboratory parameters, including platelet count, coagulation markers, fibrinogen, and liver enzymes, between approximately 72% and 93% of contributing patient data were derived from grade 3 studies. Similarly, survival data were largely supported by higher-quality studies. Reporting of haemorrhagic manifestations relied more evenly on grade 2 and grade 3 studies, reflecting the broader variability in how bleeding outcomes were defined and reported across the literature. Taken together, these findings suggest that the principal laboratory abnormalities identified in this review are supported predominantly by moderate- to high-quality observational studies, although the inherent limitations of retrospective and non-analytical study designs should still be considered when interpreting the findings.

There are varying approaches when deciding whether to include or exclude lower quality studies [36]. The inclusion of such studies here was with the intention of increasing the data available for analysis given the variation in amount and type of data reported across the other studies. It is important to acknowledge that observational studies, particularly non-analytical designs such as case series, are inherently at greater risk of bias. Nevertheless, they remain critical in the context of HFRS, where outbreaks are sporadic and severe cases relatively rare. These studies provide indispensable detail on the clinical presentation and laboratory abnormalities observed in affected patients, forming the basis of our understanding of disease mechanisms, including potential pathways leading to haemostatic dysfunction. Their inclusion in this review was necessary to capture the breadth of available data and to synthesise clinical and laboratory features relevant to loss of haemostasis.

Despite this, the limitations of the available evidence became increasingly apparent as the review progressed. Data reporting across studies was highly variable, with inconsistent measurement and incomplete presentation of laboratory clotting parameters. Very few studies were primarily focused on the loss of haemostasis and development of haemorrhagic manifestations in HFRS as the primary research question. The data presented here were largely gathered opportunistically from studies reporting on general disease severity, renal failure, and risk stratification. As a result, it was not possible to perform a robust meta-analysis linking specific laboratory parameters to bleeding risk. This highlights a critical gap in the literature: while individual studies describe abnormalities in platelet counts and other clotting parameters, there is a missing step in integrating these findings into predictive models of clinical outcome.

An additional consideration is that most cohorts included in this review were hospital-based. Across all hantavirus types, the majority of patients were reported as hospital inpatients, particularly for HTNV, DOBV, and SEOV infections. PUUV cohorts contained a small proportion of outpatient cases, although most patients were also hospitalised. This reflects the design of many observational studies of HFRS, which typically recruit patients presenting to hospital with clinically apparent disease. Consequently, milder or subclinical infections occurring in the community are likely underrepresented. This may partly explain why the case fatality rate reported for HTNV-associated HFRS in this review is higher than that reported in population-based surveillance studies [15]. The findings of this review should therefore be interpreted as reflecting the haemostatic profiles of patients with clinically recognised HFRS, particularly those severe enough to present to healthcare services. In addition, whilst most cases were severe enough to require hospitalisation, the observational nature of the included studies also means that differences in disease severity between individual cohorts likely exist and should be considered when interpreting the findings. Nevertheless, characterising haemostatic abnormalities in hospitalised patients remains clinically important, as these individuals represent those most likely to develop clinically significant bleeding complications.

This review represents the first synthesis of observational data specifically focusing on haemostatic dysfunction in HFRS. By collating and appraising the existing evidence, we provide a foundation upon which future studies can build. Well-designed, prospective studies with standardised protocols for measuring and reporting haemostatic parameters are urgently needed. Although platelet count was the most consistently abnormal haemostatic parameter in this review, the available aggregate observational data do not support prioritising any single laboratory marker as an early or reliable predictor of progression to severe bleeding. Future studies should therefore collect serial platelet count, PT, APTT, fibrinogen, D-dimer, and liver enzyme measurements alongside standardised bleeding outcomes, enabling development of robust models to predict bleeding risk and identify high-risk patients. Additionally, investigating the role of platelet dysfunction, disturbances of the intrinsic and extrinsic clotting pathways, and fibrinolysis in HFRS could lead to targeted therapies that address these specific aspects of the disease. Other techniques for assessing real-time clot dynamics, such as thromboelastography, have been successfully used in CCHF and should be considered for use in haemostasis-related HFRS research [69]. Until such data are available, conclusions regarding the mechanisms and predictors of haemostatic dysfunction in HFRS must remain tentative, and the findings of this review should be interpreted in that context.

In conclusion, hantaviruses are an emerging zoonotic public health threat. If current epidemiological trends continue, we may expect to see more frequent and severe epidemics of HFRS in Europe and Asia, including areas not currently considered at risk. This systematic review provides a comprehensive overview of the coagulation abnormalities and haemorrhagic manifestations observed in HFRS. While thrombocytopenia and mild coagulation abnormalities are common, their exact contribution to haemorrhagic risk remains uncertain. Overall, the published literature does not present a coherent mechanistic explanation for the pathogenesis of bleeding in HFRS, with multiple processes likely contributing in parallel but without a single unifying pathway consistently supported across studies. Further research is essential to elucidate the pathophysiology of coagulation disturbances in HFRS and develop effective strategies for managing haemorrhagic complications in epidemic situations.

## Supporting information

S1 Table Summary of the 55 studies included in this review, listed alphabetically by country. [45–47,59,60,62–68,70–112]. For each study, authorship, country, year of publication, sample size, study design, and quality grading (score out of 3, with 3 = highest) are shown.
(PDF)

S2 Table Weighted average median and mean values for laboratory variables of adult patients. The combined sample size, number of publications and patient cohorts that the weighted averages are derived from are shown. Values are demonstrated for all included HFRS patients with available data, as well as stratified by causative hantavirus and reported disease severity. Values denoted with an asterisk (*) indicate median/mean values derived from one study only. ‘NR’ indicates patient groups where no data were reported. (ALT = alanine aminotransferase; APTT = Activated partial thromboplastin time; AST = aspartate aminotransferase; DOBV = Dobrava virus; HTNV = Hantaan virus; NR = not reported; PT = prothrombin time; PUUV = Puumala virus; SEOV = Seoul virus).
(PDF)

S3 Table Weighted average median and mean values for laboratory variables of paediatric patients. The combined sample size, number of publications and patient cohorts that the weighted averages are derived from are shown. Values are demonstrated for all included HFRS patients with available data, as well as stratified by causative hantavirus and reported disease severity. Values denoted with an asterisk (*) indicate median/mean values derived from one study only. ‘NR’ indicates patient groups where no data were reported. (ALT = alanine aminotransferase; APTT = Activated partial thromboplastin time; AST = aspartate aminotransferase; DOBV = Dobrava virus; HTNV = Hantaan virus; NR = not reported; PT = prothrombin time; PUUV = Puumala virus; SEOV = Seoul virus).
(PDF)

S4 Table Other haemorrhagic manifestations among adult patients with confirmed HFRS, stratified by causative hantavirus species. The sample size number (n=) denotes the number of patients with available data on haemorrhagic manifestations included in this analysis. Frequencies of each manifestation are shown. (DOBV = Dobrava virus; HTNV = Hantaan virus; PUUV = Puumala virus; SEOV = Seoul virus).
(PDF)

S5 Table Structured summary of principal findings and limitations of the evidence. Study quality grades refer to the review-specific grading system based on JBI/NIH critical appraisal tools, where Grade 3 represents the highest methodological quality and Grade 1 the lowest. These categories are not equivalent to GRADE certainty ratings and should be interpreted alongside the observational design, heterogeneity, and reporting limitations of the included studies. This table provides a structured summary of principal findings; detailed study characteristics, sample sizes, outcome-specific denominators, and study quality gradings are provided in the corresponding manuscript figures and tables and in the accompanying supplementary tables. (APTT = activated partial thromboplastin time; CFR = case fatality rate; DOBV = Dobrava virus; GRADE = Grading of Recommendations Assessment, Development and Evaluation; HFRS = haemorrhagic fever with renal syndrome; HTNV = Hantaan virus; JBI = Joanna Briggs Institute; NIH = National Institutes of Health; PT = prothrombin time; PUUV = Puumala virus; SEOV = Seoul virus.)
(PDF)

S1 Fig Preferred Reporting Items for Systematic Reviews and Meta-Analyses (PRISMA) 2020 checklist. Completed PRISMA 2020 checklist indicating where each reporting item is addressed in the manuscript, including page references and supporting excerpts where relevant. From: Page MJ, McKenzie JE, Bossuyt PM, Boutron I, Hoffmann TC, Mulrow CD, et al. The PRISMA 2020 statement: an updated guideline for reporting systematic reviews. BMJ 2021;372:n71. doi: 10.1136/bmj.n71.
(PDF)
