# Supplementary material for: Bleeding in haemorrhagic fever with renal syndrome: A systematic review characterising the loss of haemostasis in hantavirus infections
Source: PLoS Negl Trop Dis. 2026 Jul 15;20(7):e0014524. doi: 10.1371/journal.pntd.0014524 (PMC13387616; doi:10.1371/journal.pntd.0014524)
Supplement: S1 Table — [45–47,59,60,62–68,70–112]. For each study, authorship, country, year of publication, sample size, study design, and quality grading (score out of 3, with 3 = highest) are shown. (PDF) [file pntd.0014524.s001.pdf]

S1 Table

Study ID	Publication	Country	Sample size	Study design	Quality grading
1	Rista et al. 2017 [65]	Albania	33	Prospective cohort study	2
2	Hatzl et al. 2023 [66]	Austria	23	Prospective cohort study	2
3	Fabian et al. 2014 [70]	Austria	82	Retrospective cohort study	2
4	Acham-Roschitz et al. 2010 [71]	Austria	19	Retrospective case series	3
5	Bakelants et al. 2020 [72]	Belgium	16	Retrospective comparative case series	3
6	Courouble et al. 2001 [73]	Belgium	13	Retrospective case series	3
7	Noack et al. 2022 [74]	Bosnia and Herzegovina	40	Retrospective cohort study	2
8	Christova et al. 2017 [75]	Bulgaria	23	Retrospective comparative case series	2
9	Liu et al. 2024 [76]	China	53	Prospective case series	3
10	Wang et al. 2023 [77]	China	76	Retrospective cohort study	2
11	Min et al. 2023 [78]	China	66	Retrospective cohort study	3
12	He et al. 2023 [79]	China	317	Retrospective cohort study	3
13	Hu et al. 2023 [62]	China	1,873	Retrospective and prospective cohort study	3
14	Li et al. 2023 [80]	China	206	Retrospective cohort study	3
15	Zhang et al. 2022 [81]	China	92	Retrospective cohort study	3
16	Du et al. 2021 [67]	China	105	Prospective cohort study	2
17	Yang et al. 2021 [82]	China	109	Retrospective cohort study	2
18	Li et al. 2018 [45]	China	41	Prospective cohort study	2
19	Yu et al. 2017 [46]	China	384	Retrospective cohort study	2
20	Zhu et al. 2015 [83]	China	100	Case-control study	2
21	Du et al. 2014 [47]	China	356	Retrospective cohort study	3
22	Wang et al. 2013 [84]	China	112	Retrospective cohort study	3
23	Zhang et al. 2011 [85]	China	131	Prospective cohort study	3
24	Han et al. 2011 [86]	China	120	Retrospective cohort study	2
25	Markotić et al. 2002 [87]	Croatia	29	Retrospective cohort study	3

26	Skarphedinsson et al. 2015 [88]	Denmark	184	Retrospective cohort study	3
27	Tarvainen et al. 2021 [89]	Finland	42	Prospective cohort study	3
28	Koskela et al. 2015 [90]	Finland	172	Prospective cohort study	3
29	Mustonen et al. 1994 [60]	Finland	32	Retrospective case series	3
30	Bermejo et al. 2022 [91]	France	387	Retrospective cohort study	3
31	Echterdiek et al. 2019 [59]	Germany	22	Retrospective cohort study	2
32	Nusshag et al. 2017 [92]	Germany	31	Case-control	2
33	Latus et al. 2015 [64]	Germany	456	Retrospective case series	3
34	Sadeghi et al. 2011 [93]	Germany	64	Case-control	2
35	Rasche et al. 2004 [94]	Germany	15	Retrospective cohort study	3
36	Martynova et al. 2016 [95]	Russia	228	Case-control	3
37	Pal et al. 2018 [68]	Slovenia	81	Prospective cohort study	2
38	Pal et al. 2005 [96]	Slovenia	25	Retrospective comparative case series	3
39	Avšič-Županc et al. 1999 [97]	Slovenia	31	Retrospective cohort study	2
40	Bren et al. 1996 [98]	Slovenia	33	Retrospective case series	3
41	Park et al. 2011 [99]	South Korea	73	Retrospective case series	3
42	Rusnak et al. 2009 [100]	South Korea	38	Prospective interventional cohort study (open-label, non-randomized)	1
43	Kim et al. 2007 [101]	South Korea	61	Retrospective cohort study	3
44	Kim et al. 2003 [102]	South Korea	144	Retrospective cohort study	2
45	Kim et al. 1995 [103]	South Korea	30	Retrospective comparative case series	2
46	Yoo et al. 1994 [104]	South Korea	63	Retrospective case series	3
47	Pon et al. 1990 [105]	South Korea	10	Retrospective cohort study with a case-control component	2
48	Bruno et al. 1990 [106]	South Korea	26	Retrospective case series	3
49	Pettersson et al. 2014 [107]	Sweden	105	Prospective cohort study	3
50	Ahlm et al. 1994 [108]	Sweden	32	Prospective and retrospective case series	3
51	Settergren et al. 1991 [63]	Sweden	958	Retrospective cohort study	2
52	Settergren et al. 1989 [109]	Sweden	74	Prospective cohort study	2
53	Kaya et al. 2020 [110]	Turkey	70	Retrospective cohort study with a diagnostic test development component	2

54	İnce et al. 2021 [111]	Turkey	20	Retrospective cohort study	3
55	Çelebi et al. 2019 [112]	Turkey	24	Retrospective cohort study (nested case-control)	3
