# Supplementary material for: Bleeding in haemorrhagic fever with renal syndrome: A systematic review characterising the loss of haemostasis in hantavirus infections
Source: PLoS Negl Trop Dis. 2026 Jul 15;20(7):e0014524. doi: 10.1371/journal.pntd.0014524 (PMC13387616; doi:10.1371/journal.pntd.0014524)
Supplement: S2 Table — The combined sample size, number of publications and patient cohorts that the weighted averages are derived from are shown. Values are demonstrated for all included HFRS patients with available data, as well as stratified by causative hantavirus and reported disease severity. Values denoted with an asterisk (*) indicate median/mean values derived from one study only. ‘NR’ indicates patient groups where no data were reported. (ALT = alanine aminotransferase; APTT = Activated partial thromboplastin time; AST = aspartate aminotransferase; DOBV = Dobrava virus; HTNV = Hantaan virus; NR = not reported; PT = prothrombin time; PUUV = Puumala virus; SEOV = Seoul virus). (PDF) [file pntd.0014524.s002.pdf]

S2 Table

Platelet count (x10⁹/L)

	All Patients	PUUV	DOBV	HTNV	SEOV	Non-severe	Severe
Weighted average median	**56.2**	**94.5**	**47.6**	**39.9**	**36.0***	**58.7**	**16.3**
Sample size	4,863	1,363	25	3,183	30	4,567	296
Number of publications	33	16	3	12	1	33	7
Number of patient cohorts	46	18	3	20	1	38	8
Weighted average mean	**84.0**	**121.1**	**40.3**	**56.7**	**65.9***	**86.0**	**20.3***
Sample size	1,699	715	52	594	51	1,649	50
Number of publications	13	5	2	4	1	13	1
Number of patient cohorts	17	5	2	6	1	16	1

PT (seconds)

	All Patients	PUUV	DOBV	HTNV	SEOV	Non-severe	Severe
Weighted average median	**12.1**	**NA**	**NR**	**12.0**	**NR**	**11.8**	**14.9**
Sample size	2,436	NA	NR	2,360	NR	2,262	174
Number of publications	5	NA	NR	4	NR	5	2
Number of patient cohorts	9	NA	NR	8	NR	6	3
Weighted average mean	**13.7**	**14.6***	**NR**	**13.7**	**NR**	**13.4**	**16.4***
Sample size	590	24	NR	476	NR	540	50
Number of publications	5	1	NR	2	NR	5	1
Number of patient cohorts	7	1	NR	4	NR	6	1

APTT (seconds)

	All Patients	PUUV	DOBV	HTNV	SEOV	Non-severe	Severe
Weighted average median	**38.8**	**35.3**	**66.3***	**38.9**	**NR**	**38.3**	**45.9**
Sample size	2,614	163	15	2,360	NR	2,440	174
Number of publications	8	3	1	4	NR	8	2
Number of patient cohorts	13	3	1	8	NR	10	3
Weighted average mean	**48.3**	**NR**	**NR**	**50.3**	**NR**	**45.9**	**72.8***
Sample size	566	NR	NR	476	NR	516	50
Number of publications	4	NR	NR	2	NR	4	1
Number of patient cohorts	6	NR	NR	4	NR	5	1

Fibrinogen (g/L)

	All Patients	PUUV	DOBV	HTNV	SEOV	Non-severe	Severe
Weighted average median	**2.57**	**5.7***	**NR**	**2.46**	**NR**	**2.63**	**1.75**
Sample size	2,303	74	NR	2,229	NR	2,143	160

Number of publications	3	1	NR	2	NR	3	1
Number of patient cohorts	6	1	NR	5	NR	4	2
Weighted average mean	**3.78**	**NR**	**NR**	**3.78**	**NR**	**3.92**	**2.71**
Sample size	581	NR	NR	581	NR	517	64
Number of publications	3	NR	NR	3	NR	3	2
Number of patient cohorts	6	NR	NR	6	NR	4	2

D-dimer (ng/mL)

	All Patients	PUUV	DOBV	HTNV	SEOV	Non-severe	Severe
Weighted average median	**1810.3**	**2800.0***	**NR**	**1720.0***	**NR**	**1810.3**	**NR**
Sample size	1,991	42	NR	1,873	NR	1,991	NR
Number of publications	3	1	NR	1	NR	3	NR
Number of patient cohorts	3	1	NR	1	NR	3	NR
Weighted average mean	**6258.8**	**NR**	**NR**	**5535.0**	**NR**	**6258.8**	**NR**
Sample size	162	NR	NR	92	NR	162	NR
Number of publications	2	NR	NR	1	NR	2	NR
Number of patient cohorts	3	NR	NR	2	NR	3	NR

Haemoglobin (g/L)

	All Patients	PUUV	DOBV	HTNV	SEOV	Non-severe	Severe
Weighted average median	**143.1**	**137.4**	**153.5***	**145.1**	**175.0***	**141.6**	**165.8**
Sample size	2,642	275	8	2,229	30	2,482	160
Number of publications	9	5	1	2	1	9	1
Number of patient cohorts	13	5	1	5	1	11	2
Weighted average mean	**138.6**	**145.1**	**148.0***	**135.6**	**NR**	**138.8**	**132.1**
Sample size	1,722	411	33	902	NR	1,657	65
Number of publications	12	2	1	4	NR	12	2
Number of patient cohorts	15	2	1	7	NR	13	2

Haematocrit (L/L)

	All Patients	PUUV	DOBV	HTNV	SEOV	Non-severe	Severe
Weighted average median	**0.42**	**0.42**	**NR**	**0.38**	**0.50***	**0.42**	**0.35**
Sample size	904	701	NR	173	30	814	90
Number of publications	7	4	NR	2	1	7	2
Number of patient cohorts	9	4	NR	4	1	7	2
Weighted average mean	**0.44**	**0.42***	**0.43***	**0.45***	**NR**	**0.44**	**NR**
Sample size	259	24	33	38	NR	259	NR
Number of publications	5	1	1	1	NR	5	NR

Number of patient cohorts	5	1	1	1	NR	5	NR

AST (U/L)

	All Patients	PUUV	DOBV	HTNV	SEOV	Non-severe	Severe
Weighted average median	**85.7**	**41.3**	**54.6***	**97.0**	**89.0***	**79.1**	**197.5**
Sample size	4,011	756	2	3,061	30	3,788	223
Number of publications	20	6	1	10	1	20	5
Number of patient cohorts	29	7	1	17	1	23	6
Weighted average mean	**171.4**	**75.5**	**105.0**	**190.0**	**177.2***	**143.4**	**751.2***
Sample size	1,084	1003	52	594	51	1,034	50
Number of publications	11	3	2	4	1	11	1
Number of patient cohorts	15	3	2	6	1	14	1

ALT (U/L)

	All Patients	PUUV	DOBV	HTNV	SEOV	Non-severe	Severe
Weighted average median	**56.1**	**45.4**	**96.7**	**58.7**	**60.0***	**53.5**	**100.8**
Sample size	4,039	822	17	3,008	30	3,816	223
Number of publications	20	7	2	9	1	20	5
Number of patient cohorts	30	8	2	16	1	24	6
Weighted average mean	**123.5**	**76.2**	**122.0***	**135.5**	**186.0***	**112.3**	**331.7***
Sample size	978	100	19	594	51	928	50
Number of publications	9	3	1	4	1	9	1
Number of patient cohorts	13	3	1	6	1	12	1
