# Supplementary material for: Bleeding in haemorrhagic fever with renal syndrome: A systematic review characterising the loss of haemostasis in hantavirus infections
Source: PLoS Negl Trop Dis. 2026 Jul 15;20(7):e0014524. doi: 10.1371/journal.pntd.0014524 (PMC13387616; doi:10.1371/journal.pntd.0014524)
Supplement: S3 Table — The combined sample size, number of publications and patient cohorts that the weighted averages are derived from are shown. Values are demonstrated for all included HFRS patients with available data, as well as stratified by causative hantavirus and reported disease severity. Values denoted with an asterisk (*) indicate median/mean values derived from one study only. ‘NR’ indicates patient groups where no data were reported. (ALT = alanine aminotransferase; APTT = Activated partial thromboplastin time; AST = aspartate aminotransferase; DOBV = Dobrava virus; HTNV = Hantaan virus; NR = not reported; PT = prothrombin time; PUUV = Puumala virus; SEOV = Seoul virus). (PDF) [file pntd.0014524.s003.pdf]

S3 Table

Platelet count (x10^9/L)

	All Patients	PUUV	DOBV	HTNV	SEOV	Non-severe	Severe
Weighted average median	**91.5**	**107.5**	**NR**	**85.2**	**NR**	**91.5**	**NR**
Sample size	374	105	NR	269	NR	374	NR
Number of publications	6	4	NR	2	NR	6	NR
Number of patient cohorts	6	4	NR	2	NR	6	NR
Weighted average mean	**NR**	**NR**	**NR**	**NR**	**NR**	**NR**	**NR**
Sample size	NR	NR	NR	NR	NR	NR	NR
Number of publications	NR	NR	NR	NR	NR	NR	NR
Number of patient cohorts	NR	NR	NR	NR	NR	NR	NR

PT (seconds)

	All Patients	PUUV	DOBV	HTNV	SEOV	Non-severe	Severe
Weighted average median	**13.2***	**NR**	**NR**	**13.2***	**NR**	**13.2***	**NR**
Sample size	206	NR	NR	206	NR	206	NR
Number of publications	1	NR	NR	1	NR	1	NR
Number of patient cohorts	1	NR	NR	1	NR	1	NR
Weighted average mean	**NR**	**NR**	**NR**	**NR**	**NR**	**NR**	**NR**
Sample size	NR	NR	NR	NR	NR	NR	NR
Number of publications	NR	NR	NR	NR	NR	NR	NR
Number of patient cohorts	NR	NR	NR	NR	NR	NR	NR

APTT (seconds)

	All Patients	PUUV	DOBV	HTNV	SEOV	Non-severe	Severe
Weighted average median	**44.0***	**NR**	**NR**	**44.0***	**NR**	**44.0***	**NR**
Sample size	206	NR	NR	206	NR	206	NR
Number of publications	1	NR	NR	1	NR	1	NR
Number of patient cohorts	1	NR	NR	1	NR	1	NR
Weighted average mean	**NR**	**NR**	**NR**	**NR**	**NR**	**NR**	**NR**
Sample size	NR	NR	NR	NR	NR	NR	NR
Number of publications	NR	NR	NR	NR	NR	NR	NR
Number of patient cohorts	NR	NR	NR	NR	NR	NR	NR

Fibrinogen (g/L)

	All Patients	PUUV	DOBV	HTNV	SEOV	Non-severe	Severe
Weighted average median	**NR**	**NR**	**NR**	**NR**	**NR**	**NR**	**NR**
Sample size	NR	NR	NR	NR	NR	NR	NR
Number of publications	NR	NR	NR	NR	NR	NR	NR

Number of patient cohorts	NR	NR	NR	NR	NR	NR	NR
Weighted average mean	**2.58***	**NR**	**NR**	**2.58***	**NR**	**2.58***	**NR**
Sample size	206	NR	NR	206	NR	206	NR
Number of publications	1	NR	NR	1	NR	1	NR
Number of patient cohorts	1	NR	NR	1	NR	1	NR

D-dimer (ng/mL)

	All Patients	PUUV	DOBV	HTNV	SEOV	Non-severe	Severe
Weighted average median	**NR**	**NR**	**NR**	**NR**	**NR**	**NR**	**NR**
Sample size	NR	NR	NR	NR	NR	NR	NR
Number of publications	NR	NR	NR	NR	NR	NR	NR
Number of patient cohorts	NR	NR	NR	NR	NR	NR	NR
Weighted average mean	**NR**	**NR**	**NR**	**NR**	**NR**	**NR**	**NR**
Sample size	NR	NR	NR	NR	NR	NR	NR
Number of publications	NR	NR	NR	NR	NR	NR	NR
Number of patient cohorts	NR	NR	NR	NR	NR	NR	NR

Haemoglobin (g/L)

	All Patients	PUUV	DOBV	HTNV	SEOV	Non-severe	Severe
Weighted average median	**131.0**	**125.0***	**NR**	**134.0***	**NR**	**131.0**	**NR**
Sample size	95	32	NR	63	NR	95	NR
Number of publications	2	1	NR	1	NR	2	NR
Number of patient cohorts	2	1	NR	1	NR	2	NR
Weighted average mean	**129.0***	**NR**	**NR**	**129.0***	**NR**	**129.0***	**NR**
Sample size	206	NR	NR	206	NR	206	NR
Number of publications	1	NR	NR	1	NR	1	NR
Number of patient cohorts	1	NR	NR	1	NR	1	NR

Haematocrit (L/L)

	All Patients	PUUV	DOBV	HTNV	SEOV	Non-severe	Severe
Weighted average median	**NR**	**NR**	**NR**	**NR**	**NR**	**NR**	**NR**
Sample size	NR	NR	NR	NR	NR	NR	NR
Number of publications	NR	NR	NR	NR	NR	NR	NR
Number of patient cohorts	NR	NR	NR	NR	NR	NR	NR
Weighted average mean	**NR**	**NR**	**NR**	**NR**	**NR**	**NR**	**NR**
Sample size	NR	NR	NR	NR	NR	NR	NR
Number of publications	NR	NR	NR	NR	NR	NR	NR
Number of patient cohorts	NR	NR	NR	NR	NR	NR	NR

AST (U/L)

	All Patients	PUUV	DOBV	HTNV	SEOV	Non-severe	Severe
Weighted average median	**73.4**	**40.0***	**NR**	**76.2**	**NR**	**73.4**	**NR**
Sample size	291	22	NR	269	NR	291	NR
Number of publications	3	1	NR	2	NR	3	NR
Number of patient cohorts	3	1	NR	2	NR	3	NR
Weighted average mean	**NR**	**NR**	**NR**	**NR**	**NR**	**NR**	**NR**
Sample size	NR	NR	NR	NR	NR	NR	NR
Number of publications	NR	NR	NR	NR	NR	NR	NR
Number of patient cohorts	NR	NR	NR	NR	NR	NR	NR

ALT (U/L)

	All Patients	PUUV	DOBV	HTNV	SEOV	Non-severe	Severe
Weighted average median	**41.9**	**37.1**	**NR**	**42.8**	**NR**	**41.9**	**NR**
Sample size	323	54	NR	269	NR	323	NR
Number of publications	4	2	NR	2	NR	4	NR
Number of patient cohorts	4	2	NR	2	NR	4	NR
Weighted average mean	**NR**	**NR**	**NR**	**NR**	**NR**	**NR**	**NR**
Sample size	NR	NR	NR	NR	NR	NR	NR
Number of publications	NR	NR	NR	NR	NR	NR	NR
Number of patient cohorts	NR	NR	NR	NR	NR	NR	NR
