# Supplementary material for: Bleeding in haemorrhagic fever with renal syndrome: A systematic review characterising the loss of haemostasis in hantavirus infections
Source: PLoS Negl Trop Dis. 2026 Jul 15;20(7):e0014524. doi: 10.1371/journal.pntd.0014524 (PMC13387616; doi:10.1371/journal.pntd.0014524)
Supplement: S4 Table — The sample size number (n=) denotes the number of patients with available data on haemorrhagic manifestations included in this analysis. Frequencies of each manifestation are shown. (DOBV = Dobrava virus; HTNV = Hantaan virus; PUUV = Puumala virus; SEOV = Seoul virus). (PDF) [file pntd.0014524.s004.pdf]

S4 Table

Bleeding site	PUUV<br>n = 1,597	HTNV<br>n = 1,265	DOBV<br>n = 60	SEOV<br>n = 81
	Freq	Freq	Freq	Freq
Major bleeding	4	2	3	0
Minor bleeding	3	0	2	0
Haemorrhagic shock	2	0	0	0
Haemorrhage requiring transfusion	1	0	0	0
Disseminated intravascular coagulation	13	0	0	0
Visible bleeding (site not specified)	0	0	5	0
Skin or oral mucosal petechiae	0	45	0	14
Haematemesis or haemoptysis	0	4	0	0
Retroperitoneal haemorrhage	0	1	0	0
Abnormal vaginal bleeding	2	0	0	0
