# Supplementary material for: Bleeding in haemorrhagic fever with renal syndrome: A systematic review characterising the loss of haemostasis in hantavirus infections
Source: PLoS Negl Trop Dis. 2026 Jul 15;20(7):e0014524. doi: 10.1371/journal.pntd.0014524 (PMC13387616; doi:10.1371/journal.pntd.0014524)
Supplement: S5 Table — Study quality grades refer to the review-specific grading system based on JBI/NIH critical appraisal tools, where Grade 3 represents the highest methodological quality and Grade 1 the lowest. These categories are not equivalent to GRADE certainty ratings and should be interpreted alongside the observational design, heterogeneity, and reporting limitations of the included studies. This table provides a structured summary of principal findings; detailed study characteristics, sample sizes, outcome-specific denominators, and study quality gradings are provided in the corresponding manuscript figures and tables and in the accompanying supplementary tables. (APTT = activated partial thromboplastin time; CFR = case fatality rate; DOBV = Dobrava virus; GRADE = Grading of Recommendations Assessment, Development and Evaluation; HFRS = haemorrhagic fever with renal syndrome; HTNV = Hantaan virus; JBI = Joanna Briggs Institute; NIH = National Institutes of Health; PT = prothrombin time; PUUV = Puumala virus; SEOV = Seoul virus.) (PDF) [file pntd.0014524.s005.pdf]

S5 Table. Structured summary of principal findings and limitations of the evidence.

Outcome domain	Principal finding	Data available	Study quality	Key interpretative limitations
Platelet count / thrombocytopenia	Thrombocytopenia was the most consistent abnormality and was more pronounced in HTNV than PUUV; severe cohorts had lower platelet counts.	Platelet counts: 6,562 adult patients from 46 studies.	75.4% of platelet data derived from Grade 3 studies.	Sampling time-point and disease phase varied across studies, and individual-level links between platelet count and bleeding outcomes could not be assessed.
PT/APTT	APTT prolongation was variably reported and was most evident in HTNV cohorts, particularly severe cases. PT prolongation was less consistent and mainly observed in severe cohorts.	PT data: 3,026 adult patients from 10 studies; APTT: 3,180 adult patients from 12 studies.	PT: 80.4% Grade 3; APTT: 76.0% Grade 3.	Sampling time-point varied, and PT/APTT methods likely differed between sites and countries, limiting direct comparison between cohorts.
Fibrinogen/D-dimer	D-dimer was elevated where reported, but fibrinogen was generally preserved, with hypofibrinogenaemia mainly observed in severe HTNV cohorts.	Fibrinogen: 2,884 adult patients from 6 studies; D-dimer: 2,153 adult patients from 5 studies.	Fibrinogen: 81.8% Grade 3; D-dimer: 93.2% Grade 3.	D-dimer was less consistently reported than other laboratory parameters and paired individual-level data were unavailable to distinguish localised coagulation/fibrinolysis from systemic activation.
Liver enzymes / hepatic involvement	AST and ALT were moderately elevated overall and higher in severe HTNV cohorts. Significant hepatic impairment did not appear to be a dominant contributor to haemostatic dysfunction.	AST: 5,095 adult patients from 31 studies; ALT: 5,017 adult patients from 29 studies.	AST: 73.6% Grade 3; ALT: 72.5% Grade 3.	Timing of sampling varied, and AST/ALT elevations may reflect systemic inflammation or disease severity rather than impaired hepatic synthetic function.
Haemorrhagic manifestations	Microscopic haematuria was common. PUUV was associated with low rates of overt bleeding. Mucocutaneous and GI bleeding were more frequent in HTNV, DOBV, SEOV. Pulmonary and intracranial haemorrhage were rarely reported.	Haemorrhagic manifestations reported for 3,003 adult patients from 36 studies.	47.8% Grade 3; 51.3% Grade 2	Bleeding definitions and ascertainment varied, microscopic haematuria may reflect renal dysfunction rather than true haemorrhage, and DOBV/SEOV samples were small.
Survival / case fatality	Case fatality was highest in DOBV and HTNV cohorts, although estimates likely reflect hospital-based cohorts with clinically apparent disease rather than population-level fatality risk.	Survival reported for 7,063 patients.	69.9% Grade 3; 29.6% Grade 2	Most cohorts were hospital-based, so case fatality estimates likely reflect clinically apparent disease rather than population-level fatality risk.
Paediatric HFRS	Paediatric HFRS was infrequently reported but appeared milder than adult disease, with fewer severe haemorrhagic manifestations. Microscopic haematuria was common, while severe bleeding was rare.	Paediatric data for 374 patients from 6 studies.	94.1% Grade 3; 5.9% Grade 2	Paediatric data were limited to PUUV and HTNV cohorts, with sparse clotting data and limited comparability across hantavirus types.
