# Supplementary material for: Bleeding in haemorrhagic fever with renal syndrome: A systematic review characterising the loss of haemostasis in hantavirus infections
Source: PLoS Negl Trop Dis. 2026 Jul 15;20(7):e0014524. doi: 10.1371/journal.pntd.0014524 (PMC13387616; doi:10.1371/journal.pntd.0014524)
Supplement: S1 Fig — Completed PRISMA 2020 checklist indicating where each reporting item is addressed in the manuscript, including page references and supporting excerpts where relevant. From: Page MJ, McKenzie JE, Bossuyt PM, Boutron I, Hoffmann TC, Mulrow CD, et al. The PRISMA 2020 statement: an updated guideline for reporting systematic reviews. BMJ 2021;372:n71. doi: 10.1136/bmj.n71. (PDF) [file pntd.0014524.s006.pdf]

PRISMA 2020 Checklist

Section and Topic	Item #	Checklist item	Location where item is reported
**TITLE**			
Title	1	Identify the report as a systematic review.<br>Title: Bleeding in haemorrhagic fever with renal syndrome: A systematic review characterising the loss of haemostasis in hantavirus infections	Page 1
**ABSTRACT**			
Abstract	2	See the PRISMA 2020 for Abstracts checklist.<br>The abstract follows the PRISMA for Abstracts 2020 checklist and the PLOS NTDs structured format, including background, methodology/principal findings, and conclusions/significance	Pages 2 – 3
**INTRODUCTION**			
Rationale	3	Describe the rationale for the review in the context of existing knowledge.<br>"An increase in the frequency and magnitude of HFRS epidemics in Europe has been partially attributed to the warming climate and increased land-use [16, 17]. The epidemic potential of HFRS could increase further with a warming climate, possibly leading to more frequent outbreaks in endemic areas, and emergence of cases in new regions."<br>"Fully understanding the mechanisms through which hypocoagulability occurs in HFRS would provide important targets for novel therapeutic agents and alter clinical management strategies. Unfortunately, routine laboratory clotting parameters provide little insight into the causes of an individual patient's haemostatic balance."	Page 7<br>Page 10
Objectives	4	Provide an explicit statement of the objective(s) or question(s) the review addresses.<br>"This systematic review aimed to characterise the laboratory clotting abnormalities and haemorrhagic manifestations observed in HFRS and explore differences between hantavirus species."	Page 10
**METHODS**			
Eligibility criteria	5	Specify the inclusion and exclusion criteria for the review and how studies were grouped for the syntheses.<br>"To be included in this review, studies had to meet all the following criteria: (1) observational study; (2) any human infection with hantaviruses causing HFRS; (3) laboratory confirmed infection using serological methods for specific IgM and/or IgG detection OR viral RNA detection using RT-PCR; (4) data reported on laboratory clotting parameters and/or haemorrhagic manifestations. Studies with any of the following were excluded: (1) suspected or confirmed infection with other pathogens; (2) reporting only severe or fatal cases of HFRS; (3) sample size <10 patients; (4) any study conducted at the same hospital site with an overlapping recruitment period to another included study; (5) studies with an interventional component without any baseline clinical data reported prior to intervention."	Page 12
Information sources	6	Specify all databases, registers, websites, organisations, reference lists and other sources searched or consulted to identify studies. Specify the date when each source was last searched or consulted.<br>"Database searches were conducted on 12th December 2024 using Medline, PubMed, CINAHL, Web of Science and Scopus."	Page 11
Search strategy	7	Present the full search strategies for all databases, registers and websites, including any filters and limits used.<br>"Table 2. Systematic review database search terms."	Pages 11-12
Selection process	8	Specify the methods used to decide whether a study met the inclusion criteria of the review, including how many reviewers screened each record and each report retrieved, whether they worked independently, and if applicable, details of automation tools used in the process.<br>"All references retrieved from the literature search were imported into Rayyan, a web-based tool designed to facilitate systematic reviews. Rayyan's duplicate detection feature was employed to identify and remove duplicate records prior to screening. Two reviewers (MR and AH) independently screened titles and abstracts against the predefined inclusion criteria within Rayyan. For studies deemed potentially relevant, full-text articles were assessed independently by the same reviewers. Rayyan's conflict detection functionality highlighted any discrepancies between reviewers' decisions. These conflicts were initially resolved through discussion between the two reviewers; if consensus could not be	Pages 12 - 13

Section and Topic	Item #	Checklist item	Location where item is reported
		reached, a third reviewer (TF) adjudicated the decisions.”	
Data collection process	9	Specify the methods used to collect data from reports, including how many reviewers collected data from each report, whether they worked independently, any processes for obtaining or confirming data from study investigators, and if applicable, details of automation tools used in the process.<br>“Data extraction was primarily conducted by MR using a standardised Excel spreadsheet and AH independently verified the accuracy of the extracted data. Any discrepancies identified were first discussed between the two reviewers to determine if they resulted from omissions or transcription errors and were corrected accordingly. If disagreements persisted, TF acted as a third reviewer to make a final decision.”	Page 13
Data items	10a	List and define all outcomes for which data were sought. Specify whether all results that were compatible with each outcome domain in each study were sought (e.g. for all measures, time points, analyses), and if not, the methods used to decide which results to collect.<br>“The following data were extracted from each included study:<br>• Study details: study design, sample size, recruitment period, and location (country, city, hospital(s))<br>• Demographic details: age and gender of participants<br>• Causative hantavirus: specific virus identified as the cause<br>• Laboratory values: as specified above<br>• Haemorrhagic manifestations: number observed, site, and severity<br>• Clinical outcome: survival versus death”	Pages 13 - 14
	10b	List and define all other variables for which data were sought (e.g. participant and intervention characteristics, funding sources). Describe any assumptions made about any missing or unclear information.<br>“Instances of missing data were recorded as such during the data extraction process. These cases were initially discussed between MR and AH to assess their potential impact on the outcomes of the review. As the review progressed, it became evident that a meta-analysis would not be feasible due to the heterogeneity of reporting and the frequent absence of key data across many studies. Following consultation with the third reviewer (TF), it was agreed that rather than attempting to impute or exclude studies with missing data, all available data would be presented as reported, and instances of missing data would be transparently noted in the results.”	Page 14
Study risk of bias assessment	11	Specify the methods used to assess risk of bias in the included studies, including details of the tool(s) used, how many reviewers assessed each study and whether they worked independently, and if applicable, details of automation tools used in the process.<br>“Quality assessment was conducted using the Joanna Briggs Institute (JBI) suite of critical appraisal tools, selected based on the identified study design [36-40]. For one interventional cohort study with a before-after design and no control group, the National Institutes of Health (NIH) Quality Assessment Tool for Before-After (Pre-Post) Studies With No Control Group was used [41].”<br><br>“Two independent reviewers (MR and AH) conducted the quality assessments at the outcome level to minimise bias and ensure consistency. Discrepancies were resolved through discussion, with a third reviewer (TF) consulted when consensus could not be reached.”	Page 15<br><br>Page 16
Effect measures	12	Specify for each outcome the effect measure(s) (e.g. risk ratio, mean difference) used in the synthesis or presentation of results.<br>“The aim of this review was to collate clinical and laboratory data on HFRS patients, with all included studies deemed to have sufficient data for this purpose during the screening process. During data extraction, it was determined that calculating measures of effect or establishing causal links was not feasible. Consequently, the review focused on describing clinical and laboratory data as a whole, and the quality of studies did not preclude the extraction of available data from any study.”	Page 15
Synthesis methods	13a	Describe the processes used to decide which studies were eligible for each synthesis (e.g. tabulating the study intervention characteristics and comparing against the planned groups for each synthesis (item #5)).<br>“The aim of this review was to collate clinical and laboratory data on HFRS patients, with all included studies deemed to have sufficient data for	

Section and Topic	Item #	Checklist item	Location where item is reported
		this purpose during the screening process. During data extraction, it was determined that calculating measures of effect or establishing causal links was not feasible. Consequently, the review focused on describing clinical and laboratory data as a whole, and the quality of studies did not preclude the extraction of available data from any study.”	Page 15
	13b	Describe any methods required to prepare the data for presentation or synthesis, such as handling of missing summary statistics, or data conversions.<br>“Instances of missing data were recorded as such during the data extraction process. These cases were initially discussed between MR and AH to assess their potential impact on the outcomes of the review. As the review progressed, it became evident that a meta-analysis would not be feasible due to the heterogeneity of reporting and the frequent absence of key data across many studies. Following consultation with the third reviewer (TF), it was agreed that rather than attempting to impute or exclude studies with missing data, all available data would be presented as reported, and instances of missing data would be transparently noted in the results.”<br>“Transformation of median data to mean data to calculate heterogeneity statistics was attempted but determined to introduce too much potential error into the sample”	Page 14<br>Page 17
	13c	Describe any methods used to tabulate or visually display results of individual studies and syntheses.<br>“Bubble plots were used to visualise the individual median and mean values reported for the various laboratory parameters. Haemorrhagic manifestations were reported as frequencies and percentages.”	Page 17
	13d	Describe any methods used to synthesize results and provide a rationale for the choice(s). If meta-analysis was performed, describe the model(s), method(s) to identify the presence and extent of statistical heterogeneity, and software package(s) used.<br>“A formal meta-analysis of laboratory clotting parameters was not performed due to substantial clinical and methodological heterogeneity across the included studies. In addition to inconsistent reporting of central tendency measures and the absence of raw datasets, there were important differences in study populations, infecting hantavirus species, timing of laboratory measurements relative to disease phase, and definitions of haemorrhagic manifestations. Studies included patients infected with different hantavirus strains which are associated with varying clinical severity and haemostatic abnormalities. Furthermore, laboratory parameters were measured at different stages of illness across studies, limiting comparability between reported values. Definitions and reporting of haemorrhagic manifestations were also inconsistent, with some studies reporting minor mucocutaneous bleeding while others reported only clinically significant haemorrhage. These sources of heterogeneity, together with inconsistent reporting of laboratory parameters and lack of variance measures, precluded reliable quantitative pooling of data, including subgroup meta-analyses for commonly reported coagulation parameters.”	Pages 16-17
	13e	Describe any methods used to explore possible causes of heterogeneity among study results (e.g. subgroup analysis, meta-regression).<br>“A formal meta-analysis of laboratory clotting parameters was not performed due to substantial clinical and methodological heterogeneity across the included studies. In addition to inconsistent reporting of central tendency measures and the absence of raw datasets, there were important differences in study populations, infecting hantavirus species, timing of laboratory measurements relative to disease phase, and definitions of haemorrhagic manifestations. Studies included patients infected with different hantavirus strains which are associated with varying clinical severity and haemostatic abnormalities. Furthermore, laboratory parameters were measured at different stages of illness across studies, limiting comparability between reported values. Definitions and reporting of haemorrhagic manifestations were also inconsistent, with some studies reporting minor mucocutaneous bleeding while others reported only clinically significant haemorrhage. These sources of heterogeneity, together with inconsistent reporting of laboratory parameters and lack of variance measures, precluded reliable quantitative pooling of data, including subgroup meta-analyses for commonly reported coagulation parameters.<br>Transformation of median data to mean data to calculate heterogeneity statistics was attempted but determined to introduce too much potential error into the sample. Instead, weighted averages were calculated using the following formula where M represents median or mean [44]:”	Pages 16 - 17
	13f	Describe any sensitivity analyses conducted to assess robustness of the synthesized results.<br>N/A - No sensitivity analyses were conducted as formal meta-analysis was not feasible due to heterogeneity in reporting and lack of raw data	N/A

Section and Topic	Item #	Checklist item	Location where item is reported
		across studies.	
Reporting bias assessment	14	Describe any methods used to assess risk of bias due to missing results in a synthesis (arising from reporting biases).<br>“As the review progressed, it became evident that a meta-analysis would not be feasible due to the heterogeneity of reporting and the frequent absence of key data across many studies. Following consultation with the third reviewer (TF), it was agreed that rather than attempting to impute or exclude studies with missing data, all available data would be presented as reported, and instances of missing data would be transparently noted in the results.”<br><br>“During data extraction, it was determined that calculating measures of effect or establishing causal links was not feasible. Consequently, the review focused on describing clinical and laboratory data as a whole, and the quality of studies did not preclude the extraction of available data from any study.”	Page 14<br><br>Page 15
Certainty assessment	15	Describe any methods used to assess certainty (or confidence) in the body of evidence for an outcome.<br><br>Certainty in the body of evidence was assessed indirectly through structured quality appraisal rather than by a formal framework such as GRADE, which was not appropriate given the descriptive and observational nature of the included studies. The Joanna Briggs Institute (JBI) suite of critical appraisal tools was used to evaluate methodological quality across domains including selection bias, confounding, measurement validity, and statistical analysis, with the NIH Before–After tool applied to one interventional cohort study. Each study was assigned a quality grade (1–3) based on adherence to methodological standards, providing an indication of confidence in the reliability of its findings. The overall confidence in the body of evidence was therefore informed by the proportion of higher-grade studies and the consistency of findings across studies with differing designs and quality levels.<br><br>“Quality assessment was conducted using the Joanna Briggs Institute (JBI) suite of critical appraisal tools, selected based on the identified study design [36–40]. For one interventional cohort study with a before-after design and no control group, the National Institutes of Health (NIH) Quality Assessment Tool for Before-After (Pre-Post) Studies With No Control Group was used [41]. Each tool consists of a checklist with questions addressing key bias domains, including selection, confounding, measurement, and statistical validity.”<br><br>“To quantify methodological quality, a scoring system was used, tallying the number of ‘Yes’ responses relative to the total applicable items in each checklist. Studies were assigned a quality grade based on this score: Grade 3 (highest quality, >70% ‘Yes’), Grade 2 (50–70% ‘Yes’), and Grade 1 (lowest quality, <50% ‘Yes’) [42, 43].”<br><br>“It is important to note that the quality grade reflects the study's adherence to the expected standards for its specific design. For example, a case series with a Grade 3 quality rating meets high standards for case series reporting but remains inherently more prone to bias than a well-conducted cohort study due to differences in study design hierarchy. Thus, a cohort study with a Grade 2 quality rating may still provide more robust evidence than a case series with a Grade 3 rating.”	Page 15<br><br>Page 16<br><br>Page 16
**RESULTS**			
Study selection	16a	Describe the results of the search and selection process, from the number of records identified in the search to the number of studies included in the review, ideally using a flow diagram.<br><br>“Fig 3. PRISMA flow diagram. Flowchart outlines the process from database searches through to final study inclusion. “n=” denotes the number of publications retrieved, excluded or included at each stage.”	Page 13

Section and Topic	Item #	Checklist item	Location where item is reported
		"Database searches yielded a total of 1,822 publications. After exclusion of duplicates and studies that did not meet screening criteria, 121 remained for in-depth review. After applying inclusion and exclusion criteria, a final total of 55 publications were included, with a total of 7,950 laboratory-confirmed cases of HFRS."	Page 17
	16b	Cite studies that might appear to meet the inclusion criteria, but which were excluded, and explain why they were excluded.<br>"Studies with any of the following were excluded: (1) suspected or confirmed infection with other pathogens; (2) reporting only severe or fatal cases of HFRS; (3) sample size <10 patients; (4) any study conducted at the same hospital site with an overlapping recruitment period to another included study; (5) studies with an interventional component without any baseline clinical data reported prior to intervention."<br><br>Note: No individual excluded studies were cited, as none met all inclusion criteria but were excluded on discretionary grounds. Exclusion reasons were predefined and applied systematically, as shown in the PRISMA flow diagram (Fig. 3), which documents numbers excluded at each stage.	Page 19
Study characteristics	17	Cite each included study and present its characteristics.<br>"S1 Table summarises the details each of the included studies."<br><br>"S1 Table. Summary of the 55 studies included in this review, listed alphabetically by country [45-47, 59, 60, 62-68, 70-112].<br>For each study, authorship, country, year of publication, sample size, study design, and quality grading (score out of 3, with 3 = highest) are shown."	Page 19<br><br>Page 49
Risk of bias in studies	18	Present assessments of risk of bias for each included study.<br><br>"Quality assessment revealed that 31 studies (56.4%) were classified as Grade 3 (highest quality), 23 studies (41.8%) as Grade 2, and 1 study as Grade 1 (lowest quality). S1 Table outlines study designs and corresponding quality grading.<br>The overall methodological quality of the included studies varied across designs. Cohort studies generally demonstrated the highest quality, with most using clearly defined inclusion criteria, standardised diagnostic methods, and reliable measurement of exposures and outcomes. These studies frequently reported demographic and clinical data comprehensively and used appropriate statistical analyses. However, some were limited by incomplete follow-up and potential selection bias where recruitment was not clearly consecutive.<br>Case series, which formed a substantial proportion of the evidence base, typically provided detailed descriptions of clinical features and laboratory findings, often supported by reliable diagnostic confirmation. Nevertheless, their methodological rigour was reduced by small sample sizes, retrospective data collection, and limited information on recruitment processes, raising concerns about representativeness.<br>Case-control studies performed well in terms of diagnostic confirmation and clear reporting of clinical data, but most lacked sample size justification and did not always provide sufficient detail on matching procedures or strategies to minimise selection bias.<br>The single interventional study used validated measurement methods and reported outcomes consistently but was constrained by a small sample size and lacked a control group, limiting interpretation. Similarly, the diagnostic test accuracy study applied appropriate reference standards and reported results transparently, but potential bias arose from limited information on participant selection and blinding.<br>Across all study designs, common strengths included the widespread use of standardised diagnostic methods and detailed clinical reporting. However, frequent limitations involved small sample sizes, retrospective data collection, incomplete reporting of recruitment strategies, and restricted representativeness, all of which should be considered when interpreting findings."<br><br>"S1 Table. Summary of the 55 studies included in this review, listed alphabetically by country [43-45, 49, 50, 52-58, 60-102].<br>For each study, authorship, country, year of publication, sample size, study design, and quality grading (score out of 3, with 3 = highest) are	Pages 19-20<br><br>Page 49

Section and Topic	Item #	Checklist item	Location where item is reported
		shown."	
Results of individual studies	19	For all outcomes, present, for each study: (a) summary statistics for each group (where appropriate) and (b) an effect estimate and its precision (e.g. confidence/credible interval), ideally using structured tables or plots.<br><br>"Detailed data on the weighted average median and mean lab parameters of adult patients are contained in S2 Table, and the values discussed here are for adult patients only. Bubble plots in Figs 2–4 depict the distribution of observed median or mean values from various patient cohorts…"<br><br>"Table 6 contains the frequency and percentages of various haemorrhagic manifestations stratified by causative hantavirus amongst adult patients. It is important to note that the sample sizes this data were derived from were significantly larger for PUUV and HTNV cases compared to SEOV and DOBV."<br><br>"Studies that reported on survival and death were used to calculate case fatality rates (CFR) for each hantavirus (Table 8). The case fatality rate for adult cases of HFRS caused by PUUV, DOBV and HTNV were 0.1%, 10.6% and 6.0% respectively."<br><br>Note: For each outcome, descriptive summary statistics (medians, means, frequencies, percentages) were presented for individual studies or pooled cohorts in structured tables (Tables 3–8, S1–S4) and visualised in bubble and bar plots (Figs 2–4). No effect estimates or confidence intervals were calculated because data heterogeneity and limited raw data precluded meta-analysis. Weighted averages were reported where feasible to represent central tendencies across cohorts.	Page 25<br><br>Page 28<br><br>Page 32
Results of syntheses	20a	For each synthesis, briefly summarise the characteristics and risk of bias among contributing studies.<br><br>"Of the 55 studies included in this systematic review, study designs were classified as follows: 35 cohort studies, 14 case series, 4 case-control studies, 1 diagnostic test accuracy study, and 1 interventional cohort study without a control group."<br><br>"Cases were reported from a total of 16 countries, predominantly from China… 4,046 cases were caused by HTNV, 2,545 by PUUV, 93 by DOBV and 81 by SEOV."<br><br>"Quality assessment revealed that 31 studies (56.4%) were classified as Grade 3 (highest quality), 23 studies (41.8%) as Grade 2, and 1 study as Grade 1 (lowest quality). S1 Table outlines study designs and corresponding quality grading."<br><br>"Cohort studies generally demonstrated the highest quality, with most using clearly defined inclusion criteria, standardised diagnostic methods, and reliable measurement of exposures and outcomes. However, some were limited by incomplete follow-up and potential selection bias where recruitment was not clearly consecutive."<br><br>"Case series… were limited by small sample sizes, retrospective data collection, and limited information on recruitment processes, raising concerns about representativeness."<br><br>Note: All 55 studies contributing to each synthesis are listed in S1 Table, which details study design, country, virus type, and sample size. Study	Page 19<br><br>Page 21<br><br>Page 19<br><br>Page 19<br><br>Page 20

Section and Topic	Item #	Checklist item	Location where item is reported
		characteristics relevant to interpretation, including geographic region, hantavirus species, and patient population, are summarised in the Results section. Risk of bias patterns were summarised narratively, with over half of studies rated Grade 3 (highest quality) and smaller retrospective series contributing greater uncertainty. Individual bubble plots (Figs 2–4) visually represent the individual studies contributing to the calculation of weighted averages, and the supplementary data tables (S2–S4) specify the number of publications and total patients underpinning each parameter. No meta-analysis was performed due to heterogeneity, and all available data were included descriptively. Given the observed heterogeneity in study design and outcome reporting, weighted averages were used to summarise laboratory parameters proportionally to study size rather than pooled estimates.	
	20b	Present results of all statistical syntheses conducted. If meta-analysis was done, present for each the summary estimate and its precision (e.g. confidence/credible interval) and measures of statistical heterogeneity. If comparing groups, describe the direction of the effect.<br><br>Results of weighted average laboratory clotting parameters and summary statistics of haemorrhagic manifestations and clinical outcome are presented in the Results section from pages 25 – 33.	Pages 25 - 33
	20c	Present results of all investigations of possible causes of heterogeneity among study results.<br>Note: No formal statistical investigations of heterogeneity (e.g. I², meta-regression) were performed due to incomplete and inconsistent reporting across studies. However, heterogeneity was explored descriptively by stratifying results by causative virus species (HTNV, DOBV, SEOV, PUUV), disease severity (severe vs non-severe), and patient age (adult vs paediatric). These subgroup comparisons suggested that observed variation reflected biological and clinical differences rather than random variation	N/A
	20d	Present results of all sensitivity analyses conducted to assess the robustness of the synthesized results.<br>Note: No formal sensitivity analyses were conducted because a quantitative meta-analysis was not feasible due to heterogeneity and incomplete reporting across studies. All eligible studies were retained in the synthesis regardless of quality to maximise transparency. Robustness was maintained through independent data extraction and verification by two reviewers, and by transparently reporting missing data and study quality in the Results and Supplementary Tables.	N/A
Reporting biases	21	Present assessments of risk of bias due to missing results (arising from reporting biases) for each synthesis assessed.<br>Note: No formal assessment of reporting bias was conducted because a quantitative meta-analysis was not feasible due to substantial heterogeneity and incomplete reporting across studies. However, potential bias due to missing results was minimised by including all eligible studies regardless of completeness of data and transparently documenting missing data in the extraction tables and Results section. This approach ensured that selective outcome reporting did not systematically exclude any studies from the synthesis.	N/A
Certainty of evidence	22	Present assessments of certainty (or confidence) in the body of evidence for each outcome assessed.<br>“Quality assessment was conducted using the Joanna Briggs Institute (JBI) suite of critical appraisal tools, selected based on the identified study design [36-40]. For one interventional cohort study with a before-after design and no control group, the National Institutes of Health (NIH) Quality Assessment Tool for Before-After (Pre-Post) Studies With No Control Group was used [41]. Each tool consists of a checklist with questions addressing key bias domains, including selection, confounding, measurement, and statistical validity. Responses to each question were recorded as "Yes," "No," "Unclear," or "Not Applicable" (NA).”<br><br>“To quantify methodological quality, a scoring system was used, tallying the number of "Yes" responses relative to the total applicable items in each checklist. Studies were assigned a quality grade based on this score: Grade 3 (highest quality, >70% "Yes"), Grade 2 (50–70% "Yes"), and Grade 1 (lowest quality, <50% "Yes") [40, 41]. It is important to note that the quality grade reflects the study's adherence to the expected standards for its specific design. For example, a case series with a Grade 3 quality rating meets high standards for case series reporting but remains inherently more prone to bias than a well-conducted cohort study due to differences in study design hierarchy. Thus, a cohort study with a Grade 2 quality rating may still provide more robust evidence than a case series with a Grade 3 rating.”	Page 15<br><br>Page 16

Section and Topic	Item #	Checklist item	Location where item is reported
		Note: No formal certainty assessment (e.g. GRADE) was conducted, as a quantitative meta-analysis was not feasible and the review focused on descriptive synthesis of clinical and laboratory data. However, the certainty of the evidence base was indirectly evaluated through methodological quality grading using the Joanna Briggs Institute (JBI) critical appraisal tools and the NIH quality tool for one study. Studies were graded from 1 (lowest) to 3 (highest) based on adherence to methodological standards, and the variability in quality and completeness of reporting was discussed in the Results section to reflect the overall confidence in the findings.	
**DISCUSSION**			
Discussion	23a	Provide a general interpretation of the results in the context of other evidence.<br><br>"Thrombocytopenia was a consistent finding across all study cohorts. The degree of thrombocytopenia was less severe in PUUV infections compared to other hantaviruses but varied significantly between groups of patients with the same causative hantavirus. This may be a result of confounding factors such as timing of blood samples or individual patient characteristics that are not possible to account for in this review. It does, however, add considerable weight to the suggestion that thrombocytopenia alone may not be the sole underlying cause for the development of haemorrhagic manifestations."<br><br>"APTT prolongation was frequently observed in both non-severe and severe cohorts of HTNV patients, and more pronounced in severe cases. Individual cohorts of PUUV and DOBV cohorts also demonstrated APTT prolongation. This potentially indicates an intrinsic clotting pathway disturbance, but as there was some degree of extrinsic pathway disturbance in more severe cases, there could be a common pathway defect or interference in fibrin formation due to high levels of D-dimers."<br><br>"Koskela et al. have previously hypothesised that a mild consumptive coagulopathy may develop in PUUV-infected patients [24]. The mild PT and APTT disturbances, elevated d-dimer levels, and generally preserved fibrinogen levels observed in this review suggest that some degree of coagulation factor consumption may occur, but not to the extent of a clinically significant DIC, indicating that additional mechanisms beyond consumption likely contribute to haemostatic dysfunction in HFRS."<br><br>"Significant hepatic impairment was not commonly observed, which contrasts with the liver injury seen with other bunyaviruses like CCHF [46]. Significant hepatic impairment, by limiting coagulation factor and thrombopoietin production, may contribute to clotting disturbance in severe cases but does not appear to play a major role in general."<br><br>"Only 374 cases of paediatric HFRS were captured by this review, equating to 4.7% of the entire sample size. This is in-keeping with epidemiological reports from Europe and Asia demonstrating that HFRS is significantly more common in adults than children [47, 48]"<br><br>"The paediatric case fatality rate was lower than that reported in adults for HTNV (2.6% vs 6.0%), and no deaths were recorded among paediatric PUUV cases. These findings support the observation that HFRS appears to be less severe in children than in adults [49-51]."	Pages 33<br><br>Page 34<br><br>Page 34<br><br>Page 34<br><br>Page 36<br><br>Page 37
	23b	Discuss any limitations of the evidence included in the review.<br><br>"The overall quality of the studies included in this review was mixed, with most studies rated as moderate to high quality, particularly the larger cohort studies by Hu et al., Settergren et al., and Latus et al., which provided robust methodology and comprehensive reporting of clinical and laboratory parameters [62-64]. Smaller, prospective studies of good methodological quality also contributed valuable insights, particularly where they included standardised diagnostic methods and detailed outcome reporting [65-68]. While these studies form a strong foundation, much of	Page 37

Section and Topic	Item #	Checklist item	Location where item is reported
		the remaining literature consists of retrospective observational studies and case series of variable quality, often constrained by small sample sizes, incomplete recruitment strategies, and heterogeneous reporting of outcomes. A further consideration is that methods of performing PT and APTT and measuring fibrinogen and D-dimer would vary between different sites.”	
	23c	Discuss any limitations of the review processes used.<br><br>“There are varying approaches when deciding whether to include or exclude lower quality studies [36]. The inclusion of such studies here was with the intention of increasing the data available for analysis given the variation in amount and type of data reported across the other studies. It is important to acknowledge that observational studies, particularly non-analytical designs such as case series, are inherently at greater risk of bias”<br><br>“Despite this, the limitations of the available evidence became increasingly apparent as the review progressed. Data reporting across studies was highly variable, with inconsistent measurement and incomplete presentation of laboratory clotting parameters.”<br><br>“As a result, it was not possible to perform a robust meta-analysis linking specific laboratory parameters to bleeding risk.”	Page 38<br><br>Page 39<br><br>Page 39
	23d	Discuss implications of the results for practice, policy, and future research.<br><br>“By collating and appraising the existing evidence, we provide a foundation upon which future studies can build. Well-designed, prospective studies with standardised protocols for measuring and reporting haemostatic parameters are urgently needed. These studies should aim to capture both laboratory profiles and clinical outcomes systematically, enabling the development of robust models to predict bleeding risk and improve clinical management. Such studies could clarify the prevalence and severity of coagulation abnormalities and identify high-risk patients. Additionally, investigating the role of platelet dysfunction, disturbances of the intrinsic and extrinsic clotting pathways, and fibrinolysis in HFRS could lead to targeted therapies that address these specific aspects of the disease. Other techniques for assessing real-time clot dynamics, such as thromboelastography, have been successfully used in CCHF and should be considered for use in haemostasis-related HFRS research [59]. Until such data are available, conclusions regarding the mechanisms and predictors of haemostatic dysfunction in HFRS must remain tentative, and the findings of this review should be interpreted in that context.”	Page 40
**OTHER INFORMATION**			
Registration and protocol	24a	Provide registration information for the review, including register name and registration number, or state that the review was not registered.<br><br>“This review was registered on PROSPERO on 25th November 2024 (CRD42024618760).”	Page 11
	24b	Indicate where the review protocol can be accessed, or state that a protocol was not prepared.<br><br>“the protocol is publicly available at https://www.crd.york.ac.uk/PROSPERO/view/CRD42024618760.”	Page 11
	24c	Describe and explain any amendments to information provided at registration or in the protocol.<br><br>See section “Protocol amendments following registration”	Pages 18-19
Support	25	Describe sources of financial or non-financial support for the review, and the role of the funders or sponsors in the review.	

PRISMA 2020 Checklist

Section and Topic	Item #	Checklist item	Location where item is reported
		Note: This review did not receive any specific funding. A formal funding statement ('The authors received no specific funding for this work') has been provided in the PLOS submission system in accordance with the journal's guidelines. No funders had any role in the design, analysis, interpretation, or decision to publish this review.	N/A
Competing interests	26	Declare any competing interests of review authors.<br><br>Note: The authors declare no competing interests. A formal competing interests statement outlining this has been provided in the PLOS submission system in accordance with the journal's guidelines. No financial, personal, or professional relationships exist that could have influenced the design, analysis, or reporting of this review."	N/A
Availability of data, code and other materials	27	Report which of the following are publicly available and where they can be found: template data collection forms; data extracted from included studies; data used for all analyses; analytic code; any other materials used in the review.<br><br>Note: All data underlying the findings of this review are available within the manuscript and its supplementary information files. No new, individual-level data were collected. Data extracted from included studies were derived entirely from published sources and are presented in the main text and supplementary tables. The full data extraction sheet and analytic code are not publicly deposited, as they contain only summary data already reported. A Data Availability Statement confirming this will be provided in the PLOS submission system.	N/A

From: Page MJ, McKenzie JE, Bossuyt PM, Boutron I, Hoffmann TC, Mulrow CD, et al. The PRISMA 2020 statement: an updated guideline for reporting systematic reviews. BMJ 2021;372:n71. doi: 10.1136/bmj.n71
