# Supplementary material for: Bleeding in haemorrhagic fever with renal syndrome: A systematic review characterising the loss of haemostasis in hantavirus infections
Source: PLoS Negl Trop Dis. 2026 Jul 15;20(7):e0014524. doi: 10.1371/journal.pntd.0014524 (PMC13387616; doi:10.1371/journal.pntd.0014524)
Supplement: Attachment — Submitted filename: Response to Reviewers.docx [file pntd.0014524.s008.docx]

Dr Matthew Riley MBChB, MRes, DTMH, MRCP(UK)

PhD student

Department of Clinical Sciences

Liverpool School of Tropical Medicine

Liverpool L3 5QA

United Kingdom

Date: 02/04/2026

**Response to Reviewers**

Manuscript title: Bleeding in haemorrhagic fever with renal syndrome: A systematic review characterising the loss of haemostasis in hantavirus infections

Manuscript ID: PNTD-D-25-02095

Dear Editors and Reviewers,

We would like to thank the Academic Editor and the reviewers for their careful evaluation of our manuscript and for the constructive comments and suggestions. We appreciate the time and effort invested in reviewing our work.

In response to the reviewers’ feedback, we have revised the manuscript accordingly. Several sections of the manuscript have been expanded or clarified to improve transparency, interpretation, and overall readability. In particular, we have:

- Expanded the Data Synthesis section of the Methods to provide a more detailed explanation of the clinical and methodological heterogeneity that precluded meta-analysis.
- Performed an additional analysis examining the contribution of studies of different methodological quality to each outcome dataset and integrated these findings into the Results and Discussion.
- Expanded the Discussion to more explicitly link the haemostatic abnormalities identified in the review with potential pathophysiological mechanisms in HFRS and to provide contextual comparison with haemostatic disturbances reported in other viral haemorrhagic fevers.
- Performed an additional analysis examining whether patients included in the reviewed cohorts were reported as hospital inpatients or outpatients and added corresponding clarifications in both the Results and Discussion regarding potential differences in disease severity between cohorts.
- Revised the presentation of figures by removing redundant figures and combining related bubble plots into multi-panel figures to improve clarity and readability.
- Added clarifications to the Methods section regarding the definition of haemorrhagic manifestations and the distinction between microscopic and macroscopic haematuria.
- Made several additional minor textual revisions and clarifications in response to reviewer suggestions.

Line numbers refer to the clean version of the revised manuscript. Please note that line references relating to figures correspond to the figure legends included in the manuscript, and the full figures have been uploaded separately as individual files in accordance with journal guidelines.

Below we provide a detailed, point-by-point response to each reviewer comment. Reviewer comments are reproduced in black text, and our responses are provided below each comment in red text. Where relevant, excerpts from the revised manuscript are included following line number references, presented in italics and quotation marks for ease of reference.

**Reviewers' Comments:**

Reviewer's Responses to Questions

**Key Review Criteria Required for Acceptance?**

As you describe the new analyses required for acceptance, please consider the following:

**Methods**

-Are the objectives of the study clearly articulated with a clear testable hypothesis stated?

-Is the study design appropriate to address the stated objectives?

-Is the population clearly described and appropriate for the hypothesis being tested?

-Is the sample size sufficient to ensure adequate power to address the hypothesis being tested?

-Were correct statistical analysis used to support conclusions?

-Are there concerns about ethical or regulatory requirements being met?

Reviewer #1: Can be improved.

Response:

We thank the reviewer for this comment. The methodological aspects highlighted in this section have been addressed in detail in our responses under Summary and General Comments, where we provide additional clarification regarding the justification for narrative synthesis, sources of heterogeneity, and the application and interpretation of the risk-of-bias assessment.

Reviewer #2: -Are the objectives of the study clearly articulated with a clear testable hypothesis stated? – Yes

-Is the study design appropriate to address the stated objectives? – Yes

-Is the population clearly described and appropriate for the hypothesis being tested? – Partly, it is difficult to draw conclusions regarding potential differences in responses due to different hantaviruses as some studies might have included only patients with severe enough disease to be hospitalized (“in-patients” staying at least one night in the hospital) whereas other might have included also patients with milder syndrome, not hospitalized but sampled at time of diagnosis. For instance, the overall case-fatality rate reported for the HTNV-caused HFRS was 6.0% (line 587). This is much higher than epidemiological data account for, see for example Zhang et al. (2021) The changing epidemiology of hemorrhagic fever with renal syndrome in Southeastern China during 1963–2020: A retrospective analysis of surveillance data. PLoS Negl Trop Dis 15(8): e0009673. They reported 114071 HFRS cases with 1269 deaths (1.1%). This suggest that data obtained from HTNV-infected HFRS-patients have been collected from individuals with a more severe disease than average. PUUV-caused HFRS has in Sweden and Finland been associated with case-fatality rates of 0.4% and 0.1%, respectively [see Hjertqvist et al. Mortality rate patterns for hemorrhagic fever with renal syndrome caused by Puumala virus. Emerg Infect Dis. 2010; Makary P, et al. Disease burden of Puumala virus infections, 1995-2008. Epidemiol Infect. 2010 Oct;138(10):1484-92.], which is more in line with data presented in line 587.

Response:

We thank the reviewer for this thoughtful observation. To address this concern, we performed an additional analysis of the included studies to determine whether patients were reported as hospital inpatients or outpatients. This analysis showed that the majority of patients across all hantavirus groups were derived from hospital-based cohorts. In particular, almost all HTNV, DOBV, and SEOV cases were reported as hospital inpatients, while PUUV cohorts contained a small proportion of outpatient cases but still consisted predominantly of hospitalised patients. These findings suggest that the datasets included in this review largely represent patients with clinically recognised HFRS who presented to healthcare services. These findings have been added to the Results section (lines 470 – 481 in revised manuscript). The added text is as follows:

*“The majority of patients included in the reviewed studies were hospitalised. Among HTNV cohorts, 3,896 of 4,046 patients (96.3%) were explicitly reported as hospital inpatients, with the remaining cases derived from studies where hospitalisation could reasonably be inferred from the study design and recruitment process. Similarly, all SEOV patients were derived from hospital-based cohorts, and 77 of 93 DOBV patients (82.8%) were explicitly reported as hospital inpatients, with the remaining cases derived from studies where hospitalisation could reasonably be inferred. Among PUUV patients, 2,101 of 2,545 (82.6%) were explicitly reported as hospitalised inpatients, while 158 patients (6.2%) were reported as outpatients in studies that included both inpatient and outpatient populations. The remaining PUUV cases were derived from studies where hospitalisation was likely but not explicitly stated or where admission status could not be determined.”*

We have added a paragraph in the Discussion highlighting that observational studies of HFRS typically recruit hospitalised patients, meaning milder or subclinical infections occurring in the community are likely underrepresented. We also acknowledge that this may partly explain why the case fatality rate observed for HTNV-associated HFRS in this review appears higher than that reported in population-based surveillance studies, including the study suggested by the reviewer (Zhang et al., 2021). The manuscript now clarifies that the findings should therefore be interpreted as reflecting the haemostatic profiles of patients with clinically recognised disease, particularly those severe enough to present to hospital (lines 811 – 828 in revised manuscript). The added text is as follows:

*“An additional consideration is that most cohorts included in this review were hospital-based. Across all hantavirus types, the majority of patients were reported as hospital inpatients, particularly for HTNV, DOBV, and SEOV infections. PUUV cohorts contained a small proportion of outpatient cases, although most patients were also hospitalised. This reflects the design of many observational studies of HFRS, which typically recruit patients presenting to hospital with clinically apparent disease. Consequently, milder or subclinical infections occurring in the community are likely underrepresented. This may partly explain why the case fatality rate reported for HTNV-associated HFRS in this review is higher than that reported in population-based surveillance studies [15]. The findings of this review should therefore be interpreted as reflecting the haemostatic profiles of patients with clinically recognised HFRS, particularly those severe enough to present to healthcare services. In addition, whilst most cases were severe enough to require hospitalisation, the observational nature of the included studies also means that differences in disease severity between individual cohorts likely exist and should be considered when interpreting the findings. Nevertheless, characterising haemostatic abnormalities in hospitalised patients remains clinically important, as these individuals represent those most likely to develop clinically significant bleeding complications.”*

Finally, we note that studies exclusively reporting severe cases were excluded during screening, which helped minimise potential bias toward unusually severe cohorts.

-Is the sample size sufficient to ensure adequate power to address the hypothesis being tested? – NA

-Were correct statistical analysis used to support conclusions? – NA, statistical analysis not performed

-Are there concerns about ethical or regulatory requirements being met? - No

Reviewer #3: (No Response)

**Results**

-Does the analysis presented match the analysis plan?

-Are the results clearly and completely presented?

-Are the figures (Tables, Images) of sufficient quality for clarity?

Reviewer #1: Can be improved.

Response:

We thank the reviewer for this comment. Issues relating to the presentation and interpretation of the results have been addressed in detail in our responses under Summary and General Comments, including clarification of the narrative synthesis approach and improved integration of the study quality assessment within the Results and Discussion sections.

Reviewer #2: -Does the analysis presented match the analysis plan? – Yes

-Are the results clearly and completely presented? – Yes

-Are the figures (Tables, Images) of sufficient quality for clarity? – Partly. Some figures can be removed, and others can be combined into panels

Response:

We thank the reviewer for this helpful suggestion. In response, we have revised the presentation of figures to improve clarity and reduce redundancy.

As noted in the general comments below in response to specific reviewer comments, Figures 20 and 21 (relative frequency charts of haemorrhagic manifestations) have been removed, as the underlying data are already presented in Tables 6 and 7.

In addition, several figures have been combined into multi-panel figures to streamline the presentation of laboratory data. The original bubble plots previously presented as individual figures (Figures 2–19 in the original manuscript) have now been reorganised into three multi-panel figures: Figures 2–7 are now presented as panels A–F in Figure 2 (lines 504 – 516 in revised manuscript); Figures 8–13 are presented as panels A–F in Figure 3 (lines 521 – 532 in revised manuscript); and Figures 14–19 are presented as panels A–F in Figure 4 (lines 541 – 554 in revised manuscript). This restructuring improves readability while preserving all graphical information.

Reviewer #3: (No Response)

**Conclusions**

-Are the conclusions supported by the data presented?

-Are the limitations of analysis clearly described?

-Do the authors discuss how these data can be helpful to advance our understanding of the topic under study?

-Is public health relevance addressed?

Reviewer #1: Can be improved.

Response:

We thank the reviewer for this comment. These points have been addressed in detail in our responses under Summary and General Comments, where we clarify the limitations of the available evidence, strengthen the discussion of study quality and heterogeneity, and expand the discussion linking the findings to broader understanding of haemostatic dysfunction in HFRS.

Reviewer #2: -Are the conclusions supported by the data presented? – Yes, with the exception that the differences observed between studies, and for different hantaviruses, might be due to differences in how ill the individuals included in the respective cohorts presented in the different articles where.

Response:

We thank the reviewer for this helpful observation. As noted above (with full excerpts provided in the response under the Methods section), we have addressed this point by performing an additional analysis examining whether patients included in the reviewed cohorts were reported as hospital inpatients or outpatients. The results of this analysis have now been summarised in the Results (lines 470 – 481 in revised manuscript), and an additional paragraph has been added to the Discussion acknowledging that most cohorts were hospital-based and that milder or subclinical infections occurring in the community may therefore be underrepresented (lines 811 – 828 in revised manuscript). The manuscript now clarifies that the findings should be interpreted as reflecting the haemostatic profiles of patients with clinically recognised HFRS presenting to healthcare services, but that differences in severity between cohorts likely exists and should be considered when interpreting the results.

-Are the limitations of analysis clearly described? – Partly, a discussion regarding potential differences in severity among the cohorts is lacking

Response:

We thank the reviewer for this helpful observation. As noted above (with full excerpts provided in the response under the Methods section), we have addressed this point by adding a paragraph to the Discussion acknowledging that most cohorts included in the review were hospital-based and that differences in disease severity between cohorts may influence the observed findings (lines 811 – 828).

-Do the authors discuss how these data can be helpful to advance our understanding of the topic under study? – Yes

-Is public health relevance addressed? - Yes

Reviewer #3: (No Response)

**Editorial and Data Presentation Modifications?**

Use this section for editorial suggestions as well as relatively minor modifications of existing data that would enhance clarity. If the only modifications needed are minor and/or editorial, you may wish to recommend “Minor Revision” or “Accept”.

Reviewer #1: (No Response)

Reviewer #2: (No Response)

Reviewer #3: (No Response)

**Summary and General Comments**

Use this section to provide overall comments, discuss strengths/weaknesses of the study, novelty, significance, general execution and scholarship. You may also include additional comments for the author, including concerns about dual publication, research ethics, or publication ethics. If requesting major revision, please articulate the new experiments that are needed.

Reviewer #1: This manuscript presents the first systematic review of hemostatic dysfunction in Hemorrhagic Fever with Renal Syndrome (HFRS). By analyzing 55 studies (comprising 7,950 laboratory-confirmed patients), it systematically characterizes coagulation parameter abnormalities and hemorrhagic manifestations associated with different hantaviruses (PUUV, DOBV, HTNV, SEOV). Key findings include: thrombocytopenia is the most consistent abnormality, more severe in HTNV, DOBV, and SEOV infections compared to PUUV; mild APTT prolongation and elevated D-dimer are commonly reported; the frequency and type of hemorrhagic manifestations (e.g., hematuria, mucocutaneous bleeding, gastrointestinal bleeding) differ by viral type; and limited pediatric data suggest a milder disease course. The review emphasizes the heterogeneity and limitations of existing evidence and calls for standardized prospective studies. I have some concerns listed below:

1. Justification for Narrative Synthesis over Meta-analysis

The manuscript notes that meta-analysis was not performed due to heterogeneity. To strengthen this methodological decision, a more detailed justification should be provided in the main text (e.g., within the Methods or Results). Please elaborate on the specific sources of clinical and methodological heterogeneity—such as differences in patient populations, hantavirus strains, timing of laboratory measurements relative to disease phase, variable definitions of bleeding, and inconsistent reporting standards across studies—that made even subgroup meta-analyses for common parameters (e.g., platelet count, PT/aPTT, fibrinogen) unfeasible.

Response:

We thank the reviewer for this helpful suggestion. We have expanded the Data Synthesis section of the Methods to provide a more detailed explanation of the clinical and methodological heterogeneity across the included studies. Specifically, we now describe differences in patient populations, infecting hantavirus species, timing of laboratory measurements relative to disease phase, and inconsistent definitions and reporting of haemorrhagic manifestations. We also clarify that the lack of variance measures and inconsistent reporting of summary statistics precluded reliable quantitative pooling of data, including subgroup meta-analyses for commonly reported parameters. The revised text can be found in the Methods section under Data synthesis (lines 321 – 335 in revised manuscript). The added text is as follows:

*“A formal meta-analysis of laboratory clotting parameters was not performed due to substantial clinical and methodological heterogeneity across the included studies. In addition to inconsistent reporting of central tendency measures and the absence of raw datasets, there were important differences in study populations, infecting hantavirus species, timing of laboratory measurements relative to disease phase, and definitions of haemorrhagic manifestations. Studies included patients infected with different hantavirus strains which are associated with varying clinical severity and haemostatic abnormalities. Furthermore, laboratory parameters were measured at different stages of illness across studies, limiting comparability between reported values. Definitions and reporting of haemorrhagic manifestations were also inconsistent, with some studies reporting minor mucocutaneous bleeding while others reported only clinically significant haemorrhage. These sources of heterogeneity, together with inconsistent reporting of laboratory parameters and lack of variance measures, precluded reliable quantitative pooling of data, including subgroup meta-analyses for commonly reported coagulation parameters.”*

2. Integration of the Risk of Bias Assessment

The abstract mentions that risk of bias was assessed using JBI and NIH tools, with studies graded 1–3. It is important to present this assessment clearly in the results—for instance, in a summary table or figure—and to integrate it into the discussion. Please clarify how the risk of bias or quality grading influenced the interpretation of the findings. For example, were certain conclusions primarily driven by lower-quality studies? A discussion of how the overall strength of the synthesized evidence is affected by study quality would greatly enhance the review’s rigor.

Response:

We thank the reviewer for this helpful suggestion. The results of the risk of bias assessment were already summarised in the Results section in the original manuscript and is retained in the revised manuscript (lines 387 – 419 in revised manuscript):

*“Of the 55 studies included in this systematic review, study designs were classified as follows: 35 cohort studies, 14 case series, 4 case-control studies, 1 diagnostic test accuracy study, and 1 interventional cohort study without a control group. Quality assessment revealed that 31 studies (56.4%) were classified as grade 3 (highest quality), 23 studies (41.8%) as grade 2, and 1 study as grade 1 (lowest quality). Detailed study characteristics, including design and quality grading, are summarised in S1 Table.*

*The overall methodological quality of the included studies varied across designs. Cohort studies generally demonstrated the highest quality, with most using clearly defined inclusion criteria, standardised diagnostic methods, and reliable measurement of exposures and outcomes. These studies frequently reported demographic and clinical data comprehensively and used appropriate statistical analyses. However, some were limited by incomplete follow-up and potential selection bias where recruitment was not clearly consecutive.*

*Case series, which formed a substantial proportion of the evidence base, typically provided detailed descriptions of clinical features and laboratory findings, often supported by reliable diagnostic confirmation. Nevertheless, their methodological rigour was reduced by small sample sizes, retrospective data collection, and limited information on recruitment processes, raising concerns about representativeness.*

*Case-control studies performed well in terms of diagnostic confirmation and clear reporting of clinical data, but most lacked sample size justification and did not always provide sufficient detail on matching procedures or strategies to minimise selection bias.*

*The single interventional study used validated measurement methods and reported outcomes consistently but was constrained by a small sample size and lacked a control group, limiting interpretation. Similarly, the diagnostic test accuracy study applied appropriate reference standards and reported results transparently, but potential bias arose from limited information on participant selection and blinding.*

*Across all study designs, common strengths included the widespread use of standardised diagnostic methods and detailed clinical reporting. However, frequent limitations involved small sample sizes, retrospective data collection, incomplete reporting of recruitment strategies, and restricted representativeness, all of which should be considered when interpreting findings.”*

In addition, the quality grading of each included study was presented in S1 Table in the original manuscript and is retained in the revised version (lines 859 – 862 in revised manuscript). To improve clarity and to better integrate study quality into the interpretation of findings, we have expanded the Results and Discussion sections.

First, we added an additional analysis examining the contribution of studies of different methodological quality to each outcome dataset. Specifically, we calculated the proportion of patients contributing to each analysed parameter that originated from studies graded as quality grade 1, 2, or 3. These results have now been reported in the Results section (lines 420 – 440 in revised manuscript). The added text is as follows:

*“To better understand how study quality contributed to the evidence base for each outcome, the proportion of patients contributing data from studies of different quality grades was examined. Across the overall cohort of 7,950 laboratory-confirmed HFRS cases, 66.2% of patients were derived from grade 3 studies, 33.3% from grade 2 studies, and 0.5% from a grade 1 study.*

*For most laboratory parameters, the majority of available data originated from higher-quality studies. Platelet count data were predominantly derived from grade 3 studies (75.4%), as were haemoglobin concentration measurements (78.6%). Similarly, prothrombin time, activated partial thromboplastin time, and fibrinogen measurements were largely contributed by grade 3 studies (80.4%, 76.0%, and 81.8%, respectively). D-dimer measurements showed the highest proportion of data originating from grade 3 studies (93.2%). Liver enzyme measurements were also largely derived from higher-quality studies, with 73.6% of aspartate aminotransferase (AST) data and 72.5% of alanine aminotransferase (ALT) data originating from grade 3 studies.*

*For clinical outcomes, survival data were predominantly derived from grade 3 studies (69.9%), with a smaller proportion from grade 2 studies (29.6%) and minimal contribution from grade 1 studies (0.5%). Reporting of haemorrhagic manifestations was more evenly distributed between grade 3 studies (47.8%) and grade 2 studies (51.3%), with a small contribution from grade 1 studies (0.9%). Data for paediatric patients were derived almost entirely from higher-quality studies, with 94.1% originating from grade 3 studies and the remainder from grade 2 studies.”*

This analysis demonstrated that the majority of the synthesised data were derived from higher-quality studies. For example, across the overall cohort 66.2% of patients originated from grade 3 studies, and for most laboratory parameters between approximately 72% and 93% of contributing patient data were derived from grade 3 studies.

Second, we expanded the Discussion to explicitly address how study quality influences interpretation of the findings (lines 773 – 786 in revised manuscript). The added text is as follows:

*“An additional analysis examining the contribution of studies of different methodological quality to each outcome demonstrated that the majority of the synthesised data were derived from higher-quality studies. Across the overall cohort, two-thirds of patients originated from studies graded as the highest quality. For most laboratory parameters, including platelet count, coagulation markers, fibrinogen, and liver enzymes, between approximately 72% and 93% of contributing patient data were derived from grade 3 studies. Similarly, survival data were largely supported by higher-quality studies. Reporting of haemorrhagic manifestations relied more evenly on grade 2 and grade 3 studies, reflecting the broader variability in how bleeding outcomes were defined and reported across the literature. Taken together, these findings suggest that the principal laboratory abnormalities identified in this review are supported predominantly by moderate- to high-quality observational studies, although the inherent limitations of retrospective and non-analytical study designs should still be considered when interpreting the findings.”*

We now highlight that the principal laboratory findings synthesised in this review are largely supported by higher-quality observational studies, while also acknowledging that some outcomes, particularly haemorrhagic manifestations, are more evenly derived from moderate-quality studies and that the observational nature of much of the literature introduces inherent limitations.

We believe these additions strengthen the transparency of the risk of bias assessment and provide clearer context regarding the strength of the evidence underlying the conclusions of the review.

3. Depth of Discussion on Pathophysiology

One of the stated aims of the review is to inform the understanding of disease mechanisms. The discussion would be strengthened by moving beyond a summary of findings to explicitly link the observed haemostatic abnormalities—such as the severity and pattern of thrombocytopenia, coagulation factor changes, and markers of consumption versus synthesis—to potential underlying mechanisms in HFRS (e.g., endothelial dysfunction, immune activation, direct viral effects, cytokine storm). Additionally, a brief comparison with haemostatic dysfunction in other viral haemorrhagic fevers would help contextualize the findings and highlight what may be distinct or shared in HFRS.

Response:

We thank the reviewer for this helpful suggestion. We have expanded the Discussion to more explicitly link the haemostatic abnormalities identified in this review with potential underlying pathophysiological mechanisms in HFRS.

Specifically, we have added additional text discussing potential mechanisms contributing to thrombocytopenia, including platelet adhesion to activated endothelium, platelet consumption, and immune-mediated platelet activation (lines 661 – 669 in revised manuscript). The added text is as follows:

*“The mechanisms underlying thrombocytopenia in HFRS are likely multifactorial. Experimental and clinical studies suggest that hantavirus infection is associated with activation of the vascular endothelium, which promotes platelet adhesion and aggregation within the microvasculature [18, 25]. In addition, immune-mediated platelet destruction and increased platelet consumption have been proposed as contributing factors [18]. Platelet activation in response to endothelial dysfunction may therefore reduce circulating platelet counts while simultaneously altering platelet function, potentially contributing to bleeding manifestations even when thrombocytopenia is only moderate in severity [23].”*

We have also expanded the interpretation of the coagulation findings to discuss how the observed pattern of mild prolongation of clotting times, elevated D-dimer levels, and generally preserved fibrinogen concentrations may reflect limited activation of coagulation pathways rather than widespread coagulation factor depletion (lines 682 – 690 in revised manuscript). The added text is as follows:

*“Taken together, this pattern of mild prolongation of coagulation times, elevated D-dimer levels, and generally preserved fibrinogen concentrations is more consistent with a limited or localised consumptive coagulopathy rather than severe systemic factor depletion [24]. Elevated D-dimer levels may reflect both activation of fibrinolysis and inflammatory processes accompanying endothelial injury. The preservation of fibrinogen levels in most cohorts suggests that widespread depletion of coagulation factors is uncommon, supporting the hypothesis that haemostatic disturbances in HFRS may arise from a combination of modest coagulation activation and endothelial-driven alterations in haemostasis rather than severe systemic consumption [24, 48].”*

In addition, we have included a new paragraph discussing the central role of endothelial dysfunction in hantavirus pathogenesis, highlighting how endothelial activation and vascular permeability may link the observed laboratory abnormalities with the clinical manifestations of haemorrhage and capillary leakage in HFRS (lines 696 – 708 in revised manuscript). The added text is as follows:

*“A central feature of hantavirus pathogenesis is infection of vascular endothelial cells, which can lead to endothelial activation and increased vascular permeability [50]. Although hantaviruses do not typically cause extensive cytopathic damage to endothelial cells, infection is associated with dysregulation of endothelial barrier function and increased permeability responses mediated through β3-integrin and VEGF signalling pathways [51]. Clinical studies in HFRS patients have also demonstrated evidence of endothelial activation, including degradation of the endothelial glycocalyx and increased circulating adhesion molecules such as soluble ICAM-1, VCAM-1, and E-selectin during the acute phase of infection [52]. These processes may contribute to both thrombocytopenia and microvascular leakage, key hallmarks of HFRS. Endothelial dysfunction may therefore represent a plausible mechanism linking the observed laboratory abnormalities with the clinical manifestations of haemorrhage and capillary leakage observed in affected patients.”*

Finally, we have added a brief comparison with other viral haemorrhagic fevers to contextualise the findings, noting that more pronounced coagulation pathway derangements and clotting factor depletion are reported in severe cases of infections such as Ebola virus disease and Crimean–Congo haemorrhagic fever (lines 709 – 718 in revised manuscript). The added text is a follows:

*“When considered alongside other viral haemorrhagic fevers, the haemostatic disturbances observed in HFRS appear to follow a somewhat distinct pattern. In infections such as Ebola virus disease and Crimean–Congo haemorrhagic fever, haemorrhagic cases are often associated with more pronounced derangements of coagulation pathways, including marked prolongation of clotting times and evidence of significant clotting factor depletion in the most severe cases, sometimes accompanied by hypofibrinogenaemia [53-56]. In contrast, the findings synthesised in this review suggest that HFRS is more commonly characterised by moderate thrombocytopenia and mild coagulation pathway disturbances rather than severe systemic depletion of coagulation factors.”*

Reviewer #2: Riley and coworkers have performed a large systemic review. The study is well performed, and the article is well written, clear and easy to read. The subject is important and although the overall conclusion is a bit disappointing (“variable study quality and incomplete reporting limit firm conclusions, underscoring the need for standardized, prospective studies of coagulation and bleeding outcomes”) it raises an important point hopefully guiding future research on this subject. Further, the data collated in this review is still off importance and interest as it shows the current knowledge and will be of help as a starting point for future studies.

Major comment:

A discussion regarding the potential impact of differences in inclusion criteria for the different cohorts included in the systemic review is warranted. For instance, if the data extracted from articles reporting on HTNV-caused HFRS is mainly from in-patients, while data from PUUV-infected are from out-patients, it can distort the conclusions.

Response:

We thank the reviewer for this important observation. To address this concern, we performed an additional analysis examining whether patients included in the reviewed cohorts were reported as hospital inpatients or outpatients. The results of this analysis, now summarised in the Results, show that the majority of patients across all hantavirus groups were derived from hospital-based cohorts (lines 470 – 481 in revised manuscript). The added text is as follows:

*“The majority of patients included in the reviewed studies were hospitalised. Among HTNV cohorts, 3,896 of 4,046 patients (96.3%) were explicitly reported as hospital inpatients, with the remaining cases derived from studies where hospitalisation could reasonably be inferred from the study design and recruitment process. Similarly, all SEOV patients were derived from hospital-based cohorts, and 77 of 93 DOBV patients (82.8%) were explicitly reported as hospital inpatients, with the remaining cases derived from studies where hospitalisation could reasonably be inferred. Among PUUV patients, 2,101 of 2,545 (82.6%) were explicitly reported as hospitalised inpatients, while 158 patients (6.2%) were reported as outpatients in studies that included both inpatient and outpatient populations. The remaining PUUV cases were derived from studies where hospitalisation was likely but not explicitly stated or where admission status could not be determined.”*

We have also added a paragraph to the Discussion acknowledging that most observational studies of HFRS recruit hospitalised patients with clinically apparent disease and that, although most cases were severe enough to require hospitalisation, differences in disease severity between individual cohorts may still exist and should be considered when interpreting the findings (lines 811 – 828 in revised manuscript). The added text is as follows:

*“An additional consideration is that most cohorts included in this review were hospital-based. Across all hantavirus types, the majority of patients were reported as hospital inpatients, particularly for HTNV, DOBV, and SEOV infections. PUUV cohorts contained a small proportion of outpatient cases, although most patients were also hospitalised. This reflects the design of many observational studies of HFRS, which typically recruit patients presenting to hospital with clinically apparent disease. Consequently, milder or subclinical infections occurring in the community are likely underrepresented. This may partly explain why the case fatality rate reported for HTNV-associated HFRS in this review is higher than that reported in population-based surveillance studies [15]. The findings of this review should therefore be interpreted as reflecting the haemostatic profiles of patients with clinically recognised HFRS, particularly those severe enough to present to healthcare services. In addition, whilst most cases were severe enough to require hospitalisation, the observational nature of the included studies also means that differences in disease severity between individual cohorts likely exist and should be considered when interpreting the findings. Nevertheless, characterising haemostatic abnormalities in hospitalised patients remains clinically important, as these individuals represent those most likely to develop clinically significant bleeding complications.”*

Minor comments:

Line 125-126. While the kidney is a target, it can be questioned if it is predominantly infected. Most likely a general systemic infection occurs, with wide-spread infection of endothelial cells in different organs such as lungs, GI, kidneys.

Response:

We thank the reviewer for this helpful clarification. The sentence has been revised to better reflect that Eurasian hantaviruses cause a systemic infection of endothelial cells across multiple organs. The revised text now states that these viruses infect microvascular endothelial cells and can disseminate to infect endothelial cells in most major organs, while noting that the renal microvasculature is an important site of infection contributing to the characteristic renal manifestations of disease (lines 125 – 128 in revised manuscript). The added text is as follows:

*“Eurasian hantaviruses infect microvascular endothelial cells and can disseminate to infect endothelial cells in most major organs. There is, however, prominent involvement of the renal microvasculature, particularly glomerular and tubular endothelial cells, contributing to the characteristic renal manifestations of disease [18].”*

Line 138-140. Please include references to this sentence.

Response:

We thank the reviewer for this suggestion. References have now been added to support this statement, citing studies demonstrating platelet activation and dysfunction in hantavirus infection (Laine et al., 2016; Connolly-Andersen et al., 2015). These references have been included in the revised manuscript (reference numbers 22 and 23 on line 138 in revised manuscript).

Line 150. Case-fatality rate for PUUV-caused HFRS in Sweden is 0.4% [Hjertqvist et al, EID, 2010].

Response:

We thank the reviewer for highlighting this reference. The sentence has been revised to better reflect the range of reported case fatality rates for PUUV infection. The text now states that the case fatality rate is typically <0.1%, with estimates from Sweden suggesting rates up to 0.4%, and the reference to Hjertqvist et al. (2010) has been added as reference number 27 (lines 150 – 153 in revised manuscript). The amended sentence is as follows:

*“PUUV infection, known as nephropathia epidemica (NE) in Europe, is generally mild with a low case fatality rate (CFR), typically <0.1%, although estimates from Sweden suggest rates up to 0.4% [12, 27].”*

Line 178-183. Maybe split this sentence into two?

Response:

We thank the reviewer for this helpful suggestion. The sentence has now been split into two to improve clarity and readability (lines 181 – 187 in revised manuscript). The amended text is as follows:

*“Widespread activation of the clotting cascade in hantavirus infection could cause a consumptive coagulopathy, indeed a disseminated intravascular coagulation (DIC), resulting in depletion of coagulation factors and diminished thrombin generation capacity. However, severe prolongation of PT and APTT reflecting significant loss of coagulation factors is rarely observed in HFRS, suggesting that this mechanism is unlikely to represent the primary pathway of haemostatic dysfunction [24].”*

Results. It would have been interesting with an analysis of possible sex-differences. However, I understand this might not be possible due to how data have been presented in the articles included in this review.

Response:

We thank the reviewer for this insightful suggestion. Unfortunately, a sub-analysis examining potential sex differences was not feasible in this review because the majority of included studies did not report laboratory parameters or haemorrhagic manifestations stratified by sex. As a result, the aggregated data available for synthesis did not permit reliable comparison between male and female patients across the combined cohort.

To address this point, we have added additional text to the Discussion highlighting that most source publications did not report data stratified by sex and that this limited our ability to explore potential sex-related differences in haemostatic abnormalities (lines 737 – 743 in revised manuscript). We now also emphasise that future studies investigating haemostatic dysfunction in HFRS should consider reporting laboratory parameters and clinical outcomes according to patient characteristics such as sex, as well as according to bleeding status, to enable more detailed analyses of factors influencing haemorrhagic risk. The added text is as follows:

*“In addition, most studies did not present laboratory or clinical data stratified by sex, preventing assessment of potential sex-related differences in haemostatic abnormalities. These limitations highlight the need for future studies specifically designed to investigate haemostatic dysfunction in HFRS, with prospective collection of standardised laboratory parameters and systematic reporting of outcomes according to bleeding status and patient characteristics such as sex.”*

Figures. The figures showing “relative frequencies” are slightly confusing. I suggest removing these figures showing the same data as presented in tables, for example Fig 21 is not needed as the data is found in Table 7.

Response:

We thank the reviewer for this helpful suggestion. We agree that the figures presenting relative frequencies of haemorrhagic manifestations duplicated data already presented in Tables 6 and 7 and may have added unnecessary complexity to the presentation of results. Accordingly, Figures 20 and 21 have been removed from the manuscript. The relevant data remain clearly presented in Table 6 (lines 588 – 592 in revised manuscript) and Table 7 (lines 622 – 628 in revised manuscript).

Reviewer #3: This manuscript addresses a clinically important and understudied aspect of hantavirus disease: the nature and patterns of haemostatic dysfunction in haemorrhagic fever with renal syndrome (HFRS). Given that bleeding manifestations are common yet poorly characterized across different hantavirus genotypes, a systematic synthesis of existing data has the potential to improve clinical recognition and inform future research into pathogenesis. The topic is timely, relevant to global health, and aligns well with the scope of PLOS Neglected Tropical Diseases.

However, despite its promising premise, the current version suffers from significant limitations in organization, logical coherence, and clarity of presentation, which must be thoroughly addressed before the manuscript can be considered for publication.

Major Concerns

1. Unclear Structure and Disrupted Logical Flow

The manuscript lacks a clear narrative progression. Sections are often disjointed, with abrupt shifts between topics—for example, elements of the Discussion contain embedded references and table captions without contextual explanation. This makes it difficult for the reader to follow the authors’ reasoning or evaluate the validity of their conclusions. A systematic review must present a transparent, reproducible logic from search strategy to synthesis; this version fails to do so.

Response:

We thank the reviewer for this comment. The Discussion section was structured to follow a logical progression typical of systematic reviews, beginning with interpretation of the principal haemostatic abnormalities identified in the included studies, followed by discussion of haemorrhagic manifestations, paediatric findings, study quality, limitations of the available literature, and priorities for future research.

References are included within the Discussion to support interpretation of the findings in the context of existing literature. We note that table captions do not appear within the Discussion section; all tables and their captions are presented in the Results section.

In response to comments from Reviewer 1, we have further expanded the Discussion to more explicitly link the observed haemostatic abnormalities to potential underlying mechanisms in HFRS, including endothelial dysfunction and platelet activation. We have also added brief contextual comparison with haemostatic disturbances reported in other viral haemorrhagic fevers. These additions strengthen the interpretative framework of the Discussion and further clarify the relationship between the findings of this review and current understanding of HFRS pathophysiology. The added text is as follows:

Lines 661 – 669 in revised manuscript:

*“The mechanisms underlying thrombocytopenia in HFRS are likely multifactorial. Experimental and clinical studies suggest that hantavirus infection is associated with activation of the vascular endothelium, which promotes platelet adhesion and aggregation within the microvasculature [18, 25]. In addition, immune-mediated platelet destruction and increased platelet consumption have been proposed as contributing factors [18]. Platelet activation in response to endothelial dysfunction may therefore reduce circulating platelet counts while simultaneously altering platelet function, potentially contributing to bleeding manifestations even when thrombocytopenia is only moderate in severity [23].”*

We have also expanded the interpretation of the coagulation findings to discuss how the observed pattern of mild prolongation of clotting times, elevated D-dimer levels, and generally preserved fibrinogen concentrations may reflect limited activation of coagulation pathways rather than widespread coagulation factor depletion (lines 682 – 690 in revised manuscript). The added text is as follows:

*“Taken together, this pattern of mild prolongation of coagulation times, elevated D-dimer levels, and generally preserved fibrinogen concentrations is more consistent with a limited or localised consumptive coagulopathy rather than severe systemic factor depletion [24]. Elevated D-dimer levels may reflect both activation of fibrinolysis and inflammatory processes accompanying endothelial injury. The preservation of fibrinogen levels in most cohorts suggests that widespread depletion of coagulation factors is uncommon, supporting the hypothesis that haemostatic disturbances in HFRS may arise from a combination of modest coagulation activation and endothelial-driven alterations in haemostasis rather than severe systemic consumption [24, 48].”*

Lines 682 – 690 in revised manuscript:

*“Taken together, this pattern of mild prolongation of coagulation times, elevated D-dimer levels, and generally preserved fibrinogen concentrations is more consistent with a limited or localised consumptive coagulopathy rather than severe systemic factor depletion [24]. Elevated D-dimer levels may reflect both activation of fibrinolysis and inflammatory processes accompanying endothelial injury. The preservation of fibrinogen levels in most cohorts suggests that widespread depletion of coagulation factors is uncommon, supporting the hypothesis that haemostatic disturbances in HFRS may arise from a combination of modest coagulation activation and endothelial-driven alterations in haemostasis rather than severe systemic consumption [24, 48].”*

Lines 696 – 708 in revised manuscript:

*“A central feature of hantavirus pathogenesis is infection of vascular endothelial cells, which can lead to endothelial activation and increased vascular permeability [50]. Although hantaviruses do not typically cause extensive cytopathic damage to endothelial cells, infection is associated with dysregulation of endothelial barrier function and increased permeability responses mediated through β3-integrin and VEGF signalling pathways [51]. Clinical studies in HFRS patients have also demonstrated evidence of endothelial activation, including degradation of the endothelial glycocalyx and increased circulating adhesion molecules such as soluble ICAM-1, VCAM-1, and E-selectin during the acute phase of infection [52]. These processes may contribute to both thrombocytopenia and microvascular leakage, key hallmarks of HFRS. Endothelial dysfunction may therefore represent a plausible mechanism linking the observed laboratory abnormalities with the clinical manifestations of haemorrhage and capillary leakage observed in affected patients.”*

Lines 709 – 718 in revised manuscript:

*“When considered alongside other viral haemorrhagic fevers, the haemostatic disturbances observed in HFRS appear to follow a somewhat distinct pattern. In infections such as Ebola virus disease and Crimean–Congo haemorrhagic fever, haemorrhagic cases are often associated with more pronounced derangements of coagulation pathways, including marked prolongation of clotting times and evidence of significant clotting factor depletion in the most severe cases, sometimes accompanied by hypofibrinogenaemia [53-56]. In contrast, the findings synthesised in this review suggest that HFRS is more commonly characterised by moderate thrombocytopenia and mild coagulation pathway disturbances rather than severe systemic depletion of coagulation factors.”*

2. Redundancy and Repetitive Content

Several paragraphs repeat similar information—particularly regarding liver enzyme elevations and bleeding symptoms—without adding new insights. For instance, descriptions of AST/ALT levels and urinary findings appear multiple times across different sections. These redundancies should be eliminated to enhance conciseness and readability.

Response:

We thank the reviewer for this comment. The manuscript reports laboratory and clinical findings following the conventional structure of systematic reviews, whereby observed data are presented in the Results section and subsequently interpreted in the Discussion.

AST and ALT values are reported once each for adult and paediatric cohorts in the Results section in the following locations:

Lines 534 – 540 in the revised manuscript for adult patients:

*“Liver function showed moderate derangements, with a weighted average median AST of 85.7 U/L and ALT of 56.1 U/L across all patients. Weighted average median AST values were higher in HTNV patients (97.0 U/L) compared to PUUV (41.3 U/L) (Figs 16 and 17), while differences in weighted average median ALT were less pronounced (58.7 U/L for HTNV vs 45.4 U/L for PUUV) (Figure 4). Both AST and ALT were higher in cohorts of severe HFRS caused by HTNV (Figure 4).”*

Lines 571 – 576 in the revised manuscript for paediatric patients:

*“The weighted average median AST was 76.2 U/L for 269 HTNV cases, derived from two publications (Fig 18). One cohort of 22 paediatric PUUV cases reported a median AST of 40.0 U/L (Fig 18). With regards to ALT, a weighted average median value of 42.8 U/L was derived from 269 cases of HTNV, compared to a weighted average median ALT of 37.1 U/L from 54 PUUV cases (Fig 20).”*

These findings are then briefly interpreted in the Discussion in the context of possible hepatic involvement in HFRS in the following locations:

Lines 691 – 695 in the revised manuscript for adult patients:

*“Significant hepatic impairment, by limiting coagulation factor and thrombopoietin production, may contribute to clotting disturbance in severe cases but does not appear to play a major role in general.”*

Lines 751 – 752 in the revised manuscript for paediatric patients:

*“ALT and AST levels were generally mildly elevated, particularly in HTNV cases.”*

Similarly, microscopic and macroscopic haematuria are reported in the Results section once each for adult and paediatric cohorts when describing the frequency of haemorrhagic manifestations in the following locations:

Lines 583 – 587 in the revised manuscript for adult patients:

*“Microscopic haematuria was a relatively common observation across all hantaviruses, present in 28.4% of SEOV cases, 26.7% of DOBV cases, 24.3% of PUUV cases, and 15.8% of HTNV cases. Macroscopic haematuria was reported more frequently in SEOV cases (19.8%), compared to HTNV (2.3%), PUUV (1.5%), and DOBV (0.0%).”*

Lines 614 – 617 in the revised manuscript for paediatric patients:

*“Microscopic haematuria was a very common observation, present in 71.4% of PUUV cases 66.7% of HTNV cases. Macroscopic haematuria was also observed in 60.3% of HTNV cases, but none of the PUUV cases.”*

These findings are then discussed once each for adult and paediatric patients in the Discussion in relation to potential renal microvascular injury in the following locations:

Lines 721 – 724 in the revised manuscript for adult patients:

*“Still, microscopic haematuria was frequently observed across all four hantavirus groups, and very common in paediatric patients with PUUV and HTNV infection. Urine dipstick may therefore be a useful screening tool when considering a diagnosis of HFRS.”*

Lines 753 – 754 in the revised manuscript for paediatric patients:

*“Microscopic haematuria was common in both PUUV and HTNV cohorts…”*

These elements were present in the original manuscript and have been retained in the revised version. Any additional appearances of AST, ALT, or haematuria occur within figure legends or table captions accompanying the presentation of the primary data. We consider this approach appropriate, as it clearly separates the reporting of results from their interpretation while ensuring that figures and tables remain interpretable when viewed independently.

For these reasons, we believe the current structure provides clarity without unnecessary redundancy, and no changes have been made to the manuscript in response to this comment.

3. Insufficient Methodological Detail

While the authors state adherence to PRISMA guidelines, key methodological components are either missing or inadequately described:

(1) No full search strategy (keywords, databases, time limits) is provided.

(2) The risk of bias assessment tool used for included studies is not specified.

(3) There is no PRISMA flow diagram showing study selection. Without these, the rigor and transparency of the review process cannot be evaluated.

Response:

We thank the reviewer for this comment. The methodological components highlighted are already reported in the original manuscript and their relative locations in the revised manuscript are included below. We have, however, made one additional clarification regarding time limits of the search strategy to further improve transparency.

The databases searched are specified in the subsection ‘Database searches’ (lines 208 – 210 in the revised manuscript):

*“Database searches were conducted on 12 December 2024 using MEDLINE, PubMed, CINAHL, Web of Science and Scopus.”*

The search terms used in the database searches are presented in Table 2 (lines 214 – 218 in the revised manuscript). To further clarify the search strategy, we have now added that no time limits were applied during database searching (lines 210 – 211 in the revised manuscript). The added text is as follows:

*“No date restrictions were applied to the database searches.”*

The tools used for quality assessment and risk of bias appraisal are specified in the subsection ‘Quality assessment’ (lines 298 – 305 in the revised manuscript):

*“Quality assessment was conducted using the Joanna Briggs Institute (JBI) suite of critical appraisal tools, selected based on the identified study design [36-40]. For one interventional cohort study with a before-after design and no control group, the National Institutes of Health (NIH) Quality Assessment Tool for Before-After (Pre-Post) Studies With No Control Group was used [41]. Each tool consists of a checklist with questions addressing key bias domains, including selection, confounding, measurement, and statistical validity. Responses to each question were recorded as "Yes," "No," "Unclear," or "Not Applicable" (NA).”*

Further details of how these tools were applied and how studies were graded are provided in lines 306 – 319 in the revised manuscript:

*“To quantify methodological quality, a scoring system was used, tallying the number of "Yes" responses relative to the total applicable items in each checklist. Studies were assigned a quality grade based on this score: Grade 3 (highest quality, >70% "Yes"), Grade 2 (50–70% "Yes"), and Grade 1 (lowest quality, <50% "Yes") [42, 43]. It is important to note that the quality grade reflects the study's adherence to the expected standards for its specific design. For example, a case series with a Grade 3 quality rating meets high standards for case series reporting but remains inherently more prone to bias than a well-conducted cohort study due to differences in study design hierarchy. Thus, a cohort study with a Grade 2 quality rating may still provide more robust evidence than a case series with a Grade 3 rating.*

*Two independent reviewers (MR and AH) conducted the quality assessments at the outcome level to minimise bias and ensure consistency. Discrepancies were resolved through discussion, with a third reviewer (TF) consulted when consensus could not be reached.”*

A PRISMA flow diagram depicting study selection is already included as Figure 1 (lines 248 – 250 in the revised manuscript):

*“****Fig 1. PRISMA flow diagram****. Flowchart outlines the process from database searches through to final study inclusion. “n=” denotes the number of publications retrieved, excluded or included at each stage.”*

These elements were present in the original manuscript and have been retained in the revised version. We hope these clarifications reassure the reviewer regarding the transparency and methodological rigour of the review process.

4. Incomplete Data Synthesis and Overinterpretation

The results focus on descriptive trends (e.g., HTNV associated with higher AST than PUUV), but due to high heterogeneity and lack of standardized reporting, no meta-analysis was performed. This is acceptable, but the discussion should more explicitly acknowledge how this limits the strength of the conclusions.

Additionally, claims about "molecular mechanisms" and "individualized management" go beyond what the available evidence supports and require softening unless clearly framed as hypotheses for future research.

Response:

We thank the reviewer for this comment. We agree that the heterogeneity of the included studies and the lack of standardised reporting limit the strength of quantitative inference that can be drawn from the available evidence. This limitation was already discussed in the original manuscript and remains in the revised manuscript in the following locations:

Lines 800 – 802 in revised manuscript:

*“Data reporting across studies was highly variable, with inconsistent measurement and incomplete presentation of laboratory clotting parameters.”*

Lines 805 – 807 in revised manuscript:

*“As a result, it was not possible to perform a robust meta-analysis linking specific laboratory parameters to bleeding risk.”*

Lines 842 – 845 in revised manuscript:

*“Until such data are available, conclusions regarding the mechanisms and predictors of haemostatic dysfunction in HFRS must remain tentative, and the findings of this review should be interpreted in that context.”*

In the revised manuscript, the Discussion has been further expanded in response to comments from Reviewer 1, including a new analysis examining the contribution of studies of different methodological quality to each outcome dataset. This additional analysis demonstrated that most of the synthesised laboratory and outcome data were derived from moderate- to high-quality studies, while also reinforcing that the overall evidence base remains limited by retrospective observational designs and heterogeneous reporting (lines 773 – 786 in revised manuscript). The added text is as follows:

*“An additional analysis examining the contribution of studies of different methodological quality to each outcome demonstrated that the majority of the synthesised data were derived from higher-quality studies. Across the overall cohort, two-thirds of patients originated from studies graded as the highest quality. For most laboratory parameters, including platelet count, coagulation markers, fibrinogen, and liver enzymes, between approximately 72% and 93% of contributing patient data were derived from grade 3 studies. Similarly, survival data were largely supported by higher-quality studies. Reporting of haemorrhagic manifestations relied more evenly on grade 2 and grade 3 studies, reflecting the broader variability in how bleeding outcomes were defined and reported across the literature. Taken together, these findings suggest that the principal laboratory abnormalities identified in this review are supported predominantly by moderate- to high-quality observational studies, although the inherent limitations of retrospective and non-analytical study designs should still be considered when interpreting the findings.”*

With regard to the second point, we respectfully note that we do not use the phrases “molecular mechanisms” or “individualized management” in the manuscript. Throughout the Discussion, mechanistic interpretations are presented cautiously and are framed as possible explanations or hypotheses to inform future research rather than definitive conclusions. Similarly, recommendations for clinical relevance are limited to broad implications for improved understanding and future study design, rather than claims of individualised management based on the current evidence base. No further changes were made in response to this comment.

Minor Suggestions

1. Consider including a summary table comparing haemostatic parameters (platelet count, PT/aPTT, fibrinogen, D-dimer) across major hantaviruses.

Response:

We thank the reviewer for this suggestion. Summary tables comparing laboratory haemostatic parameters across the major hantaviruses were already included in the original manuscript and have been retained in the revised manuscript as outlined below.

Specifically, S2 Table (lines 863 – 872 in the revised manuscript) summarises the weighted average median and mean values for laboratory parameters among adult patients, including platelet count, PT, APTT, fibrinogen, D-dimer, AST, ALT, haemoglobin, and haematocrit, stratified by causative hantavirus (PUUV, DOBV, HTNV, and SEOV). Similarly, S3 Table (lines 873 – 881 in the revised manuscript) provides the corresponding weighted average laboratory values for paediatric patients stratified by hantavirus.

These tables were included to provide a concise overview of the haemostatic laboratory findings across the different hantaviruses. No changes were made to the manuscript in response to this comment.

2. Clarify early on whether “bleeding” refers only to clinically evident hemorrhage or includes laboratory-defined coagulopathy.

Response:

We thank the reviewer for this helpful suggestion. In this review, haemorrhagic manifestations refer to clinically reported bleeding events, whereas laboratory parameters such as platelet count, PT, APTT, fibrinogen, and D-dimer were extracted separately as markers of haemostatic disturbance. Patients with laboratory abnormalities alone were not classified as having haemorrhagic manifestations unless clinically evident or radiologically confirmed bleeding was documented.

To clarify this distinction, we have added additional wording in the Data extraction subsection of the Methods describing how clinical bleeding and laboratory abnormalities were recorded in the review (lines 264 – 271 in revised manuscript). The added text is as follows:

*“Haemorrhagic manifestations refer to clinically reported bleeding events described in the included studies (e.g., petechiae, mucosal bleeding, haematuria, gastrointestinal bleeding, pulmonary haemorrhage, intracranial haemorrhage). Microscopic haematuria refers to blood in the urine detectable only on laboratory testing (e.g., urine microscopy or dipstick), whereas macroscopic haematuria refers to visibly blood-stained urine. Patients with laboratory abnormalities alone were not recorded as having haemorrhagic manifestations unless clinically evident or radiologically confirmed bleeding was documented.”*

3. Define terms such as “microscopic hematuria” vs “macroscopic hematuria” for non-specialist readers.

Response:

We thank the reviewer for this helpful suggestion. A brief clarification defining microscopic haematuria and macroscopic haematuria has now been added to the Data extraction subsection of the Methods to aid clarity for non-specialist readers (lines 266 – 269 in revised manuscript). The added text is as follows:

*“Microscopic haematuria refers to blood in the urine detectable only on laboratory testing (e.g., urine microscopy or dipstick), whereas macroscopic haematuria refers to visibly blood-stained urine.”*

Yours sincerely,

Dr Matthew Riley

On behalf of all authors
