# Supplementary material for: Bleeding in haemorrhagic fever with renal syndrome: A systematic review characterising the loss of haemostasis in hantavirus infections
Source: PLoS Negl Trop Dis. 2026 Jul 15;20(7):e0014524. doi: 10.1371/journal.pntd.0014524 (PMC13387616; doi:10.1371/journal.pntd.0014524)
Supplement: Attachment — Submitted filename: Response_to_Reviewers_auresp_2.docx [file pntd.0014524.s009.docx]

Dr Matthew Riley MBChB, MRes, DTMH, MRCP(UK)

PhD student

Department of Clinical Sciences

Liverpool School of Tropical Medicine

Liverpool L3 5QA

United Kingdom

Date: 16/06/2026

Response to Reviewers

Manuscript title: Bleeding in haemorrhagic fever with renal syndrome: A systematic review characterising the loss of haemostasis in hantavirus infections

Manuscript ID: PNTD-D-25-02095R1

Dear Editors and Reviewers,

We would like to thank the Academic Editor and the reviewers for their further evaluation of our manuscript and for the constructive comments and suggestions. We appreciate the time and effort invested in reviewing our revised work.

We are grateful that Reviewers 1 and 2 considered the manuscript substantially improved, with Reviewer 1 recommending acceptance and Reviewer 2 raising only one minor point for clarification. We have addressed the remaining comments below.

Where additional clarification could improve the manuscript, we have revised the text. Where requested changes would require unsupported mechanistic or prognostic inference beyond the available data, we have explained our rationale for retaining a cautious interpretation.

Line numbers refer to the track changes version of the revised manuscript unless otherwise specified. Please note that line references relating to figures correspond to the figure legends included in the manuscript, and the full figures have been uploaded separately as individual files in accordance with journal guidelines.

Below we provide a detailed, point-by-point response to each reviewer comment. Reviewer comments are reproduced in black text, and our responses are provided below each comment in red text. Where relevant, excerpts from the revised manuscript are included following line number references, presented in italics and quotation marks for ease of reference.

Reviewers' Comments

Reviewer's Responses to Questions

Key Review Criteria Required for Acceptance?

As you describe the new analyses required for acceptance, please consider the following:

Methods

-Are the objectives of the study clearly articulated with a clear testable hypothesis stated?

-Is the study design appropriate to address the stated objectives?

-Is the population clearly described and appropriate for the hypothesis being tested?

-Is the sample size sufficient to ensure adequate power to address the hypothesis being tested?

-Were correct statistical analysis used to support conclusions?

-Are there concerns about ethical or regulatory requirements being met?

Reviewer #1: (No Response)

Reviewer #2: (No Response)

Reviewer #3: (No Response)

Results

-Does the analysis presented match the analysis plan?

-Are the results clearly and completely presented?

-Are the figures (Tables, Images) of sufficient quality for clarity?

Reviewer #1: (No Response)

Reviewer #2: Yes

Reviewer #3: (No Response)

Conclusions

-Are the conclusions supported by the data presented?

-Are the limitations of analysis clearly described?

-Do the authors discuss how these data can be helpful to advance our understanding of the topic under study?

-Is public health relevance addressed?

Reviewer #1: (No Response)

Reviewer #2: Yes

Reviewer #3: (No Response)

Editorial and Data Presentation Modifications?

Use this section for editorial suggestions as well as relatively minor modifications of existing data that would enhance clarity. If the only modifications needed are minor and/or editorial, you may wish to recommend “Minor Revision” or “Accept”.

Reviewer #1: (No Response)

Reviewer #2: In the discussion, the new text "A central feature of hantavirus pathogenesis is infection of vascular endothelial cells, which can lead to endothelial activation and increased vascular permeability [50]. Although hantaviruses do not typically cause extensive cytopathic damage to endothelial cells, infection is associated with dysregulation of endothelial barrier function and increased permeability responses mediated through β3-integrin and VEGF signalling pathways [51]" is not completely correct.

Ref 50 is to an article published 2008, and Ref 51 is to a review published 2014. Since then other studies have shown other mechanisms than "β3-integrin and VEGF signalling pathways" can contribute to, and are likely important for, the increased vascular permeability. I suggest either removing this part or expand on it with a more updated discussion.

Response:

We thank the reviewer for this helpful clarification. We agree that the previous wording may have placed undue emphasis on β3-integrin and VEGF signalling pathways as the principal mechanisms of increased vascular permeability in HFRS. To avoid over-specifying mechanisms that are not central to the findings of this review, we have removed the sentence referring specifically to dysregulation of endothelial barrier function mediated through β3-integrin and VEGF signalling pathways.

The revised paragraph now retains the broader, better-supported point that endothelial infection is associated with dysregulation of endothelial barrier function and increased vascular permeability, without implying that β3-integrin and VEGF signalling are the only or dominant mechanisms. The revised text states (lines 700 - 702 in revised manuscript):

“A central feature of hantavirus pathogenesis is infection of vascular endothelial cells, which is associated with dysregulation of endothelial barrier function and increased vascular permeability [50, 51].”

Reviewer #3: (No Response)

Summary and General Comments

Use this section to provide overall comments, discuss strengths/weaknesses of the study, novelty, significance, general execution and scholarship. You may also include additional comments for the author, including concerns about dual publication, research ethics, or publication ethics. If requesting major revision, please articulate the new experiments that are needed.

Reviewer #1: The authors have done an excellent job revising the manuscript. They have strengthened the justification for narrative synthesis by detailing specific sources of heterogeneity, integrated the risk of bias assessment by quantifying each quality grade's contribution to the evidence, and expanded the discussion to link haemostatic abnormalities with endothelial dysfunction, platelet activation, and other viral haemorrhagic fevers. Additional analyses of inpatient versus outpatient cohorts and clearer definitions further improve transparency. The manuscript is now methodologically rigorous and clearly presented. I recommend acceptance.

Response:

We thank Reviewer 1 for their supportive comments and recommendation for acceptance. We are grateful for their constructive feedback during the first round of review, which helped strengthen the manuscript.

Reviewer #2: The authors have answered all my questions, and the manuscript has greatly improved. I have no further questions, only a minor comment about the discussion.

Response:

We thank Reviewer 2 for their supportive comments and for noting the improvements made to the manuscript. We have addressed the minor discussion-related comment above under Editorial and Data Presentation Modifications.

Reviewer #3: The authors have made a commendable effort in addressing the technical comments from the first round of reviews, particularly in refining the data quality assessment and visualization. This systematic review addresses a significant gap in the literature regarding haemostatic dysfunction in HFRS. However, in its current form, the manuscript remains a descriptive summary of isolated laboratory findings rather than a high-quality scientific synthesis. The "logic gap" between data presentation and mechanistic insight remains substantial. To meet the publication standards of PLOS Neglected Tropical Diseases, a more rigorous organizational and analytical framework is required.

Response:

We thank the reviewer for recognising the efforts made in revising the manuscript and for acknowledging that the review addresses an important gap in the literature. We respectfully note that the revised manuscript already goes beyond descriptive reporting by linking the observed laboratory abnormalities to plausible mechanisms of haemostatic dysfunction in HFRS. In particular, the Discussion links thrombocytopenia to endothelial activation, platelet adhesion, platelet consumption, and immune-mediated platelet dysfunction; discusses the pattern of elevated D-dimer with relatively preserved fibrinogen and coagulation times in relation to limited or localised consumptive coagulopathy rather than severe systemic factor depletion; and considers the role of endothelial dysfunction and microvascular permeability in linking laboratory abnormalities with clinical haemorrhagic manifestations.

We agree, however, that mechanistic interpretation must remain clearly grounded in the data presented. We have therefore reviewed the Discussion again to ensure that interpretation remains directly linked to the findings of this review and does not overstate conclusions that cannot be supported by the available observational data.

1. From Descriptive Reporting to Mechanistic Synthesis

Reviewer comment: The Discussion section currently lists potential mechanisms (e.g., endothelial dysfunction, immune activation) in a generic manner that is disconnected from the specific findings in the Results. The logic needs to flow from "Specific Observation" to "Specific Mechanism." The authors must move beyond general pathogenesis and use the data to drive the mechanistic hypothesis. For example:

(1) The results show that SEOV is associated with a high rate of macroscopic haematuria (19.8%). The authors should specifically discuss why SEOV might cause more severe renal microvascular damage or localized coagulopathy compared to PUUV (e.g., differences in receptor tropism, vascular bed specificity, or inflammatory response intensity).

Response:

We thank the reviewer for this comment. We agree that the higher proportion of macroscopic haematuria reported among SEOV cases is an interesting observation. However, we do not consider the available data sufficient to support virus-specific mechanistic conclusions regarding SEOV-related renal microvascular damage, receptor tropism, vascular bed specificity, or inflammatory response intensity.

The analysis of haemorrhagic manifestations included only 81 SEOV patients, compared with 1,597 PUUV patients and 1,265 HTNV patients. The manuscript already highlights this limitation in the Discussion and cautions against overinterpretation of the apparently higher bleeding rates in SEOV patients. The relevant text states (lines 743 – 746 in revised manuscript):

“The data reported here suggest highest rates of bleeding in SEOV patients. Caution should be used when interpreting this, however, given the significantly smaller sample size of SEOV patients included in this review.”

We believe this cautious interpretation is more appropriate than proposing SEOV-specific mechanisms that cannot be established from the available observational data. No further mechanistic inference regarding SEOV has therefore been added.

(2) The finding of significantly elevated D-dimer with relatively preserved fibrinogen and PT is a distinct phenotype. The authors should discuss whether this indicates a "localized" fibrinolysis/coagulation within the renal microcirculation or a unique form of endotheliopathy-driven coagulopathy, rather than systemic consumptive DIC.

Response:

We thank the reviewer for highlighting this important point. This phenotype is already discussed in detail in the revised manuscript. The Discussion specifically addresses the pattern of mild prolongation of coagulation times, elevated D-dimer levels, and generally preserved fibrinogen concentrations, and interprets this as being more consistent with limited or localised consumptive coagulopathy and endothelial-driven haemostatic disturbance than with severe systemic factor depletion. The relevant text states (lines 686 – 694 in revised manuscript):

“Taken together, this pattern of mild prolongation of coagulation times, elevated D-dimer levels, and generally preserved fibrinogen concentrations is more consistent with a limited or localised consumptive coagulopathy rather than severe systemic factor depletion [24]. Elevated D-dimer levels may reflect both activation of fibrinolysis and inflammatory processes accompanying endothelial injury. The preservation of fibrinogen levels in most cohorts suggests that widespread depletion of coagulation factors is uncommon, supporting the hypothesis that haemostatic disturbances in HFRS may arise from a combination of modest coagulation activation and endothelial-driven alterations in haemostasis rather than severe systemic consumption [24, 48].”

We therefore consider that the manuscript already addresses this point directly. We have retained this cautious interpretation because the included studies did not provide paired individual-level haemostatic, renal, and bleeding outcome data that would allow more specific conclusions regarding renal microcirculatory fibrinolysis or localised coagulation.

2. Enhancing Data Synthesis and Addressing Heterogeneity

Reviewer comment: While I acknowledge that a formal meta-analysis was not feasible, the heavy reliance on "weighted averages" is scientifically limiting given the acknowledged high heterogeneity. A single average value can be misleading when it collapses data from vastly different cohorts (e.g., mild outpatient PUUV vs. severe ICU HTNV cases).

(1) I recommend providing Box plots or Forest plots (without a pooled estimate) for key parameters such as Platelet counts and APTT in the Supplementary Materials. This is essential for readers to understand the range and spread of the data across studies, rather than just the central tendency.

Response:

We thank the reviewer for this suggestion. We agree that weighted averages alone would be insufficient to convey the heterogeneity of the included data. For this reason, the revised manuscript already includes bubble plots for every laboratory parameter, with individual median and mean values shown at the cohort level.

These figures were specifically included to show the range and spread of values across studies rather than relying solely on central tendency measures. Each point represents an individual study cohort, while bubble size denotes sample size, colour denotes causative hantavirus, and symbols denote severe and paediatric cohorts. The manuscript states (lines 495 – 496 in revised manuscript):

“Bubble plots in Figs 2–4 depict the distribution of observed median or mean values from various patient cohorts, including paediatric cohorts.”

The Fig 2 legend further explains (lines 510 – 515 in revised manuscript):

“Each point represents a median or mean value from an individual study cohort. The size of each point represents the sample size on a natural logarithmic scale… Bubble colour denotes causative hantavirus. Points denoted with a ‘←’ symbol represent severe cohorts, and points denoted with a ‘⋇’ symbol represent paediatric cohorts.”

We do not consider forest plots appropriate for these data because the review does not estimate effect sizes and most included studies did not report variance measures. Similarly, box plots based on study-level summary statistics would duplicate the information already presented in the bubble plots while removing information on cohort sample size, causative hantavirus, and severe cohort status. For these reasons, no additional box plots or forest plots have been added.

(2) Instead of global averages, the authors should provide a clearer comparative analysis between "Severe" and "Non-severe" cohorts (where data allows) to highlight the dynamic and severity-dependent nature of these haemostatic disturbances.

Response:

We thank the reviewer for this comment. Severe and non-severe cohorts are already reported descriptively in the Results where the source data allowed this comparison. The Results report that severe cases showed more marked thrombocytopenia compared with non-severe cases, greater PT prolongation, more marked APTT prolongation, mild hypofibrinogenaemia in severe HTNV cases, and higher AST and ALT values in severe HTNV cohorts. Severe cohorts are also visually denoted in Figs 2–4 using a specific symbol, allowing comparison with non-severe cohorts across laboratory parameters.

The relevant Results text includes the following:

“Severe cases showed more marked thrombocytopenia compared to non-severe cases (16.3 x 10⁹/L vs 58.7 x 10⁹/L) (Fig 2).” (Lines 500 – 501 in revised manuscript).

“PT prolongation was noted in severe cases (14.9 s) compared to non-severe (11.8 s) (Fig 2).” (Lines 502 – 503 in revised manuscript).

“APTT was overall slightly prolonged (weighted average median 38.8 s), with more marked prolongation in severe cases compared to non-severe (weighted average median 45.9 s vs 38.3 s) (Fig 2).” (Lines 503 – 505 in revised manuscript).

“Fibrinogen levels were generally normal (weighted average median 2.57 g/L), though mild hypofibrinogenemia was noted in severe HTNV cases (weighted average median 1.75 g/L) (Fig 3).” (Lines 519 – 521 in revised manuscript).

“Both AST and ALT were higher in cohorts of severe HFRS caused by HTNV (Fig 4).” (Lines 540 – 541 in revised manuscript).

The Discussion also interprets these severity-related patterns, particularly for PT, APTT, fibrinogen, D-dimer, and hepatic involvement. However, formal inferential comparison between severe and non-severe cohorts was not possible because severity definitions differed between studies, data were reported at cohort rather than individual-patient level, and variance measures were inconsistently reported. We therefore consider the current descriptive and visual comparison to be the most appropriate synthesis within the limitations of the available evidence.

3. Clinical Utility and Actionable Insights

Reviewer comment: As a comprehensive review of 7,950 cases, the manuscript should provide actionable insights for the clinical and research communities. Currently, the conclusion is too generic.

(1) Please include a "Summary of Findings" table utilizing the GRADE framework (or a similar structured approach). This should provide a snapshot of the quality of evidence and the certainty of the findings for each major clinical outcome (e.g., bleeding risk, severity of thrombocytopenia).

Response:

We thank the reviewer for this suggestion. We considered whether a formal GRADE Summary of Findings table would be appropriate for this review. However, the review did not generate pooled effect estimates, assess intervention effects, evaluate diagnostic test performance, or estimate prognostic effect sizes. Instead, it synthesised heterogeneous observational data reported across studies with variable designs, reporting standards, sampling time-points, and definitions of haemorrhagic outcomes.

For these reasons, we do not consider a formal GRADE assessment appropriate, as assigning categorical certainty ratings could imply a level of methodological precision not supported by the available data. The manuscript already includes a detailed quality assessment of the included studies, including study-level quality grading in S1 Table and an additional analysis quantifying the proportion of data for each outcome derived from studies of different methodological quality. This analysis is integrated into both the Results and Discussion.

However, we agree that a structured summary of the principal findings would improve readability and help readers interpret the main findings in the context of data availability and study quality. We have therefore added a new supplementary table, S5 Table, which summarises the principal findings, available sample sizes, contribution from higher-quality studies, and key interpretative limitations for each major outcome domain (lines 1242 – 1253 in revised manuscript). This table is intended to provide a structured overview of the evidence base without implying formal GRADE certainty ratings. We have also signposted this table in the opening paragraph of the Discussion (lines 651 – 653 in revised manuscript).

(2) Based on this large-scale synthesis, which laboratory marker(s) should be prioritized by clinicians as the earliest or most reliable predictor of progression to severe bleeding? The conclusion needs to offer more than just a call for "more research"; it should provide a data-driven roadmap for clinical monitoring.

Response:

We thank the reviewer for this comment. We agree that the review should provide useful direction for clinicians and researchers. However, the available data do not permit identification of any laboratory marker as the earliest or most reliable predictor of progression to severe bleeding. The included studies generally reported aggregate cohort-level data rather than individual patient-level longitudinal data, and most did not stratify laboratory parameters according to haemorrhagic versus non-haemorrhagic outcomes. As a result, identifying predictive markers of severe bleeding would require inference beyond the available evidence.

We have therefore retained a cautious interpretation to avoid overstating the predictive value of any individual laboratory marker. To address the reviewer’s comment, we have revised the Discussion to explicitly state that platelet count was the most consistently abnormal haemostatic parameter identified in this review, but that the current evidence does not support prioritising any single laboratory marker as an early or reliable predictor of severe bleeding. We have also added a more specific roadmap for future studies, highlighting the need for serial measurement of platelet count, PT, APTT, fibrinogen, D-dimer, and liver enzymes alongside standardised bleeding outcomes. The revised text states (lines 846 – 852 in revised manuscript):

*“Although platelet count was the most consistently abnormal haemostatic parameter in this review, the available aggregate observational data do not support prioritising any single laboratory marker as an early or reliable predictor of progression to severe bleeding. Future studies should therefore collect serial platelet count, PT, APTT, fibrinogen, D-dimer, and liver enzyme measurements alongside standardised bleeding outcomes, enabling development of robust models to predict bleeding risk and identify high-risk patients.”*

Yours sincerely,

Dr Matthew Riley

On behalf of all authors
