# Supplementary material for: Bleeding in haemorrhagic fever with renal syndrome: A systematic review characterising the loss of haemostasis in hantavirus infections
Source: PLoS Negl Trop Dis. 2026 Jul 15;20(7):e0014524. doi: 10.1371/journal.pntd.0014524 (PMC13387616; doi:10.1371/journal.pntd.0014524)
Supplement: Peer review history

# Peer review: Decision Letter 0

**DOI:** 10.1371/journal.pntd.0014524.r001

**Authors:** Shu Shen, Mabel Carabali

6 Feb 2026

PNTD-D-25-02095

Bleeding in haemorrhagic fever with renal syndrome: A systematic review characterising the loss of haemostasis in hantavirus infections

PLOS Neglected Tropical Diseases

Dear Dr. Riley,

Thank you for submitting your manuscript to PLOS Neglected Tropical Diseases. After careful consideration, we feel that it has merit but does not fully meet PLOS Neglected Tropical Diseases's publication criteria as it currently stands. Therefore, we invite you to submit a revised version of the manuscript that addresses the points raised during the review process.

Please submit your revised manuscript within by Apr 07 2026 11:59PM. If you will need more time than this to complete your revisions, please reply to this message or contact the journal office at plosntds@plos.org. When you're ready to submit your revision, log on to https://www.editorialmanager.com/pntd/ and select the 'Submissions Needing Revision' folder to locate your manuscript file.

Please include the following items when submitting your revised manuscript:

* A letter that responds to each point raised by the editor and reviewer(s). You should upload this letter as a separate file labeled 'Response to Reviewers'. This file does not need to include responses to any formatting updates and technical items listed in the 'Journal Requirements' section below.

* A marked-up copy of your manuscript that highlights changes made to the original version. You should upload this as a separate file labeled 'Revised Manuscript with Track Changes'.

* An unmarked version of your revised paper without tracked changes. You should upload this as a separate file labeled 'Manuscript'.

If you would like to make changes to your financial disclosure, competing interests statement, or data availability statement, please make these updates within the submission form at the time of resubmission. Guidelines for resubmitting your figure files are available below the reviewer comments at the end of this letter.

We look forward to receiving your revised manuscript.

Kind regards,

Shu Shen

Academic Editor

PLOS Neglected Tropical Diseases

Mabel Carabali

Section Editor

PLOS Neglected Tropical Diseases

Shaden Kamhawi

co-Editor-in-Chief

PLOS Neglected Tropical Diseases

orcid.org/0000-0003-4304-636XX

Paul Brindley

co-Editor-in-Chief

PLOS Neglected Tropical Diseases

orcid.org/0000-0003-1765-0002

**Journal Requirements:**

We ask that a manuscript source file is provided at Revision. Please upload your manuscript file as a .doc, .docx, .rtf or .tex. If you are providing a .tex file, please upload it under the item type u2018LaTeX Source Fileu2019 and leave your .pdf version as the item type u2018Manuscriptu2019.

**Reviewers' Comments:**

Reviewer's Responses to Questions

**Key Review Criteria Required for Acceptance?**

As you describe the new analyses required for acceptance, please consider the following:

**Methods**

-Are the objectives of the study clearly articulated with a clear testable hypothesis stated?

-Is the study design appropriate to address the stated objectives?

-Is the population clearly described and appropriate for the hypothesis being tested?

-Is the sample size sufficient to ensure adequate power to address the hypothesis being tested?

-Were correct statistical analysis used to support conclusions?

-Are there concerns about ethical or regulatory requirements being met?

Reviewer #1: Can be improved.

Reviewer #2: -Are the objectives of the study clearly articulated with a clear testable hypothesis stated? - Yes

-Is the study design appropriate to address the stated objectives? - Yes

-Is the population clearly described and appropriate for the hypothesis being tested? – Partly, it is difficult to draw conclusions regarding potential differences in responses due to different hantaviruses as some studies might have included only patients with severe enough disease to be hospitalized (“in-patients” staying at least one night in the hospital) whereas other might have included also patients with milder syndrome, not hospitalized but sampled at time of diagnosis. For instance, the overall case-fatality rate reported for the HTNV-caused HFRS was 6.0% (line 587). This is much higher than epidemiological data account for, see for example Zhang et al. (2021) The changing epidemiology of hemorrhagic fever with renal syndrome in Southeastern China during 1963–2020: A retrospective analysis of surveillance data. PLoS Negl Trop Dis 15(8): e0009673. They reported 114071 HFRS cases with 1269 deaths (1.1%). This suggest that data obtained from HTNV-infected HFRS-patients have been collected from individuals with a more severe disease than average. PUUV-caused HFRS has in Sweden and Finland been associated with case-fatality rates of 0.4% and 0.1%, respectively [see Hjertqvist et al. Mortality rate patterns for hemorrhagic fever with renal syndrome caused by Puumala virus. Emerg Infect Dis. 2010; Makary P, et al. Disease burden of Puumala virus infections, 1995-2008. Epidemiol Infect. 2010 Oct;138(10):1484-92.], which is more in line with data presented in line 587.

-Is the sample size sufficient to ensure adequate power to address the hypothesis being tested? - NA

-Were correct statistical analysis used to support conclusions? – NA, statistical analysis not performed

-Are there concerns about ethical or regulatory requirements being met? - No

Reviewer #3: (No Response)

**********

**Results**

-Does the analysis presented match the analysis plan?

-Are the results clearly and completely presented?

-Are the figures (Tables, Images) of sufficient quality for clarity?

Reviewer #1: Can be improved.

Reviewer #2: -Does the analysis presented match the analysis plan? - Yes

-Are the results clearly and completely presented? - Yes

-Are the figures (Tables, Images) of sufficient quality for clarity? – Partly. Some figures can be removed, and others can be combined into panels

Reviewer #3: (No Response)

**********

**Conclusions**

-Are the conclusions supported by the data presented?

-Are the limitations of analysis clearly described?

-Do the authors discuss how these data can be helpful to advance our understanding of the topic under study?

-Is public health relevance addressed?

Reviewer #1: Can be improved.

Reviewer #2: -Are the conclusions supported by the data presented? – Yes, with the exception that the differences observed between studies, and for different hantaviruses, might be due to differences in how ill the individuals included in the respective cohorts presented in the different articles where.

-Are the limitations of analysis clearly described? – Partly, a discussion regarding potential differences in severity among the cohorts is lacking

-Do the authors discuss how these data can be helpful to advance our understanding of the topic under study? - Yes

-Is public health relevance addressed? - Yes

Reviewer #3: (No Response)

**********

**Editorial and Data Presentation Modifications?**

Use this section for editorial suggestions as well as relatively minor modifications of existing data that would enhance clarity. If the only modifications needed are minor and/or editorial, you may wish to recommend “Minor Revision” or “Accept”.

Reviewer #1: (No Response)

Reviewer #2: (No Response)

Reviewer #3: (No Response)

**********

**Summary and General Comments**

Use this section to provide overall comments, discuss strengths/weaknesses of the study, novelty, significance, general execution and scholarship. You may also include additional comments for the author, including concerns about dual publication, research ethics, or publication ethics. If requesting major revision, please articulate the new experiments that are needed.

Reviewer #1: This manuscript presents the first systematic review of hemostatic dysfunction in Hemorrhagic Fever with Renal Syndrome (HFRS). By analyzing 55 studies (comprising 7,950 laboratory-confirmed patients), it systematically characterizes coagulation parameter abnormalities and hemorrhagic manifestations associated with different hantaviruses (PUUV, DOBV, HTNV, SEOV). Key findings include: thrombocytopenia is the most consistent abnormality, more severe in HTNV, DOBV, and SEOV infections compared to PUUV; mild APTT prolongation and elevated D-dimer are commonly reported; the frequency and type of hemorrhagic manifestations (e.g., hematuria, mucocutaneous bleeding, gastrointestinal bleeding) differ by viral type; and limited pediatric data suggest a milder disease course. The review emphasizes the heterogeneity and limitations of existing evidence and calls for standardized prospective studies. I have some concerns listed below:

1. Justification for Narrative Synthesis over Meta-analysis

The manuscript notes that meta-analysis was not performed due to heterogeneity. To strengthen this methodological decision, a more detailed justification should be provided in the main text (e.g., within the Methods or Results). Please elaborate on the specific sources of clinical and methodological heterogeneity—such as differences in patient populations, hantavirus strains, timing of laboratory measurements relative to disease phase, variable definitions of bleeding, and inconsistent reporting standards across studies—that made even subgroup meta-analyses for common parameters (e.g., platelet count, PT/aPTT, fibrinogen) unfeasible.

2. Integration of the Risk of Bias Assessment

The abstract mentions that risk of bias was assessed using JBI and NIH tools, with studies graded 1–3. It is important to present this assessment clearly in the results—for instance, in a summary table or figure—and to integrate it into the discussion. Please clarify how the risk of bias or quality grading influenced the interpretation of the findings. For example, were certain conclusions primarily driven by lower-quality studies? A discussion of how the overall strength of the synthesized evidence is affected by study quality would greatly enhance the review’s rigor.

3. Depth of Discussion on Pathophysiology

One of the stated aims of the review is to inform the understanding of disease mechanisms. The discussion would be strengthened by moving beyond a summary of findings to explicitly link the observed haemostatic abnormalities—such as the severity and pattern of thrombocytopenia, coagulation factor changes, and markers of consumption versus synthesis—to potential underlying mechanisms in HFRS (e.g., endothelial dysfunction, immune activation, direct viral effects, cytokine storm). Additionally, a brief comparison with haemostatic dysfunction in other viral haemorrhagic fevers would help contextualize the findings and highlight what may be distinct or shared in HFRS.

Reviewer #2: Riley and coworkers have performed a large systemic review. The study is well performed, and the article is well written, clear and easy to read. The subject is important and although the overall conclusion is a bit disappointing (“variable study quality and incomplete reporting limit firm conclusions, underscoring the need for standardized, prospective studies of coagulation and bleeding outcomes”) it raises an important point hopefully guiding future research on this subject. Further, the data collated in this review is still off importance and interest as it shows the current knowledge and will be of help as a starting point for future studies.

Major comment:

A discussion regarding the potential impact of differences in inclusion criteria for the different cohorts included in the systemic review is warranted. For instance, if the data extracted from articles reporting on HTNV-caused HFRS is mainly from in-patients, while data from PUUV-infected are from out-patients, it can distort the conclusions.

Minor comments:

Line 125-126. While the kidney is a target, it can be questioned if it is predominantly infected. Most likely a general systemic infection occurs, with wide-spread infection of endothelial cells in different organs such as lungs, GI, kidneys.

Line 138-140. Please include references to this sentence.

Line 150. Case-fatality rate for PUUV-caused HFRS in Sweden is 0.4% [Hjertqvist et al, EID, 2010].

Line 178-183. Maybe split this sentence into two?

Results. It would have been interesting with an analysis of possible sex-differences. However, I understand this might not be possible due to how data have been presented in the articles included in this review.

Figures. The figures showing “relative frequencies” are slightly confusing. I suggest removing these figures showing the same data as presented in tables, for example Fig 21 is not needed as the data is found in Table 7.

Reviewer #3: This manuscript addresses a clinically important and understudied aspect of hantavirus disease: the nature and patterns of haemostatic dysfunction in haemorrhagic fever with renal syndrome (HFRS). Given that bleeding manifestations are common yet poorly characterized across different hantavirus genotypes, a systematic synthesis of existing data has the potential to improve clinical recognition and inform future research into pathogenesis. The topic is timely, relevant to global health, and aligns well with the scope of PLOS Neglected Tropical Diseases.

However, despite its promising premise, the current version suffers from significant limitations in organization, logical coherence, and clarity of presentation, which must be thoroughly addressed before the manuscript can be considered for publication.

Major Concerns

1. Unclear Structure and Disrupted Logical Flow

The manuscript lacks a clear narrative progression. Sections are often disjointed, with abrupt shifts between topics—for example, elements of the Discussion contain embedded references and table captions without contextual explanation. This makes it difficult for the reader to follow the authors’ reasoning or evaluate the validity of their conclusions. A systematic review must present a transparent, reproducible logic from search strategy to synthesis; this version fails to do so.

2. Redundancy and Repetitive Content

Several paragraphs repeat similar information—particularly regarding liver enzyme elevations and bleeding symptoms—without adding new insights. For instance, descriptions of AST/ALT levels and urinary findings appear multiple times across different sections. These redundancies should be eliminated to enhance conciseness and readability.

3. Insufficient Methodological Detail

While the authors state adherence to PRISMA guidelines, key methodological components are either missing or inadequately described:

(1) No full search strategy (keywords, databases, time limits) is provided.

(2) The risk of bias assessment tool used for included studies is not specified.

(3) There is no PRISMA flow diagram showing study selection. Without these, the rigor and transparency of the review process cannot be evaluated.

4. Incomplete Data Synthesis and Overinterpretation

The results focus on descriptive trends (e.g., HTNV associated with higher AST than PUUV), but due to high heterogeneity and lack of standardized reporting, no meta-analysis was performed. This is acceptable, but the discussion should more explicitly acknowledge how this limits the strength of the conclusions. Additionally, claims about "molecular mechanisms" and "individualized management" go beyond what the available evidence supports and require softening unless clearly framed as hypotheses for future research.

Minor Suggestions

1. Consider including a summary table comparing haemostatic parameters (platelet count, PT/aPTT, fibrinogen, D-dimer) across major hantaviruses.

2. Clarify early on whether “bleeding” refers only to clinically evident hemorrhage or includes laboratory-defined coagulopathy.

3. Define terms such as “microscopic hematuria” vs “macroscopic hematuria” for non-specialist readers.

**********

PLOS authors have the option to publish the peer review history of their article (what does this mean?). If published, this will include your full peer review and any attached files.

If you choose “no”, your identity will remain anonymous but your review may still be made public.

**Do you want your identity to be public for this peer review?**  For information about this choice, including consent withdrawal, please see our Privacy Policy.

Reviewer #1: No

Reviewer #2: No

Reviewer #3: No

[NOTE: If reviewer comments were submitted as an attachment file, they will be attached to this email and accessible via the submission site. Please log into your account, locate the manuscript record, and check for the action link "View Attachments". If this link does not appear, there are no attachment files.]

**Figure resubmission:**  While revising your submission, we strongly recommend that you use PLOS’s NAAS tool (https://ngplosjournals.pagemajik.ai/artanalysis) to test your figure files. NAAS can convert your figure files to the TIFF file type and meet basic requirements (such as print size, resolution), or provide you with a report on issues that do not meet our requirements and that NAAS cannot fix.

After uploading your figures to PLOS’s NAAS tool - https://ngplosjournals.pagemajik.ai/artanalysis, NAAS will process the files provided and display the results in the "Uploaded Files" section of the page as the processing is complete. If the uploaded figures meet our requirements (or NAAS is able to fix the files to meet our requirements), the figure will be marked as "fixed" above. If NAAS is unable to fix the files, a red "failed" label will appear above. When NAAS has confirmed that the figure files meet our requirements, please download the file via the download option, and include these NAAS processed figure files when submitting your revised manuscript.

**Reproducibility:**

To enhance the reproducibility of your results, we recommend that authors of applicable studies deposit laboratory protocols in protocols.io, where a protocol can be assigned its own identifier (DOI) such that it can be cited independently in the future. Additionally, PLOS ONE offers an option to publish peer-reviewed clinical study protocols. Read more information on sharing protocols at https://plos.org/protocols?utm_medium=editorial-email&utm_source=authorletters&utm_campaign=protocols

# Author comment: Author response to Decision Letter 1

**DOI:** 10.1371/journal.pntd.0014524.r002

6 Apr 2026

Attachment Submitted filename: Response to Reviewers.docx

# Peer review: Decision Letter 1

**DOI:** 10.1371/journal.pntd.0014524.r003

**Authors:** Shu Shen, Mabel Carabali

14 May 2026

PNTD-D-25-02095R1

Bleeding in haemorrhagic fever with renal syndrome: A systematic review characterising the loss of haemostasis in hantavirus infections

PLOS Neglected Tropical Diseases

Dear Dr. Riley,

Thank you for submitting your manuscript to PLOS Neglected Tropical Diseases. After careful consideration, we feel that it has merit but does not fully meet PLOS Neglected Tropical Diseases's publication criteria as it currently stands. Therefore, we invite you to submit a revised version of the manuscript that addresses the points raised during the review process.

Please submit your revised manuscript within by Jun 13 2026 11:59PM. If you will need more time than this to complete your revisions, please reply to this message or contact the journal office at plosntds@plos.org. When you're ready to submit your revision, log on to https://www.editorialmanager.com/pntd/ and select the 'Submissions Needing Revision' folder to locate your manuscript file.

Please include the following items when submitting your revised manuscript:

* A letter that responds to each point raised by the editor and reviewer(s). You should upload this letter as a separate file labeled 'Response to Reviewers'. This file does not need to include responses to any formatting updates and technical items listed in the 'Journal Requirements' section below.

* A marked-up copy of your manuscript that highlights changes made to the original version. You should upload this as a separate file labeled 'Revised Manuscript with Track Changes'.

* An unmarked version of your revised paper without tracked changes. You should upload this as a separate file labeled 'Manuscript'.

If you would like to make changes to your financial disclosure, competing interests statement, or data availability statement, please make these updates within the submission form at the time of resubmission. Guidelines for resubmitting your figure files are available below the reviewer comments at the end of this letter.

As the corresponding author, your ORCID iD is verified in the submission system and will appear in the published article. PLOS supports the use of ORCID, and we encourage all coauthors to register for an ORCID iD and use it as well. Please encourage your coauthors to verify their ORCID iD within the submission system before final acceptance, as unverified ORCID iDs will not appear in the published article. Only the individual author can complete the verification step; PLOS staff cannot verify ORCID iDs on behalf of authors.

We look forward to receiving your revised manuscript.

Kind regards,

Shu Shen

Academic Editor

PLOS Neglected Tropical Diseases

Mabel Carabali

Section Editor

PLOS Neglected Tropical Diseases

Shaden Kamhawi

co-Editor-in-Chief

PLOS Neglected Tropical Diseases

orcid.org/0000-0003-4304-636XX

Paul Brindley

co-Editor-in-Chief

PLOS Neglected Tropical Diseases

orcid.org/0000-0003-1765-0002

**Reviewers' Comments:**

Reviewer's Responses to Questions

**Key Review Criteria Required for Acceptance?**

As you describe the new analyses required for acceptance, please consider the following:

**Methods**

-Are the objectives of the study clearly articulated with a clear testable hypothesis stated?

-Is the study design appropriate to address the stated objectives?

-Is the population clearly described and appropriate for the hypothesis being tested?

-Is the sample size sufficient to ensure adequate power to address the hypothesis being tested?

-Were correct statistical analysis used to support conclusions?

-Are there concerns about ethical or regulatory requirements being met?

Reviewer #1: (No Response)

Reviewer #2: (No Response)

Reviewer #3: (No Response)

**********

**Results**

-Does the analysis presented match the analysis plan?

-Are the results clearly and completely presented?

-Are the figures (Tables, Images) of sufficient quality for clarity?

Reviewer #1: (No Response)

Reviewer #2: Yes

Reviewer #3: (No Response)

**********

**Conclusions**

-Are the conclusions supported by the data presented?

-Are the limitations of analysis clearly described?

-Do the authors discuss how these data can be helpful to advance our understanding of the topic under study?

-Is public health relevance addressed?

Reviewer #1: (No Response)

Reviewer #2: Yes

Reviewer #3: (No Response)

**********

**Editorial and Data Presentation Modifications?**

Use this section for editorial suggestions as well as relatively minor modifications of existing data that would enhance clarity. If the only modifications needed are minor and/or editorial, you may wish to recommend “Minor Revision” or “Accept”.

Reviewer #1: (No Response)

Reviewer #2: In the discussion, the new text "A central feature of hantavirus pathogenesis is infection of vascular endothelial cells, which can lead to endothelial activation and increased vascular permeability [50]. Although hantaviruses do not typically cause extensive cytopathic damage to endothelial cells, infection is associated with dysregulation of endothelial barrier function and increased permeability responses mediated through β3-integrin and VEGF signalling pathways [51]" is not completely correct.

Ref 50 is to an article published 2008, and Ref 51 is to a review published 2014. Since then other studies have shown other mechanisms than "β3-integrin and VEGF signalling pathways" can contribute to, and are likely important for, the increased vascular permeability. I suggest either removing this part or expand on it with a more updated discussion.

Reviewer #3: (No Response)

**********

**Summary and General Comments**

Use this section to provide overall comments, discuss strengths/weaknesses of the study, novelty, significance, general execution and scholarship. You may also include additional comments for the author, including concerns about dual publication, research ethics, or publication ethics. If requesting major revision, please articulate the new experiments that are needed.

Reviewer #1: The authors have done an excellent job revising the manuscript. They have strengthened the justification for narrative synthesis by detailing specific sources of heterogeneity, integrated the risk of bias assessment by quantifying each quality grade's contribution to the evidence, and expanded the discussion to link haemostatic abnormalities with endothelial dysfunction, platelet activation, and other viral haemorrhagic fevers. Additional analyses of inpatient versus outpatient cohorts and clearer definitions further improve transparency. The manuscript is now methodologically rigorous and clearly presented. I recommend acceptance.

Reviewer #2: The authors have answered all my questions, and the manuscript has greatly improved. I have no further questions, only a minor comment about the discussion.

Reviewer #3: The authors have made a commendable effort in addressing the technical comments from the first round of reviews, particularly in refining the data quality assessment and visualization. This systematic review addresses a significant gap in the literature regarding haemostatic dysfunction in HFRS. However, in its current form, the manuscript remains a descriptive summary of isolated laboratory findings rather than a high-quality scientific synthesis. The "logic gap" between data presentation and mechanistic insight remains substantial. To meet the publication standards of PLOS Neglected Tropical Diseases, a more rigorous organizational and analytical framework is required.

1. From Descriptive Reporting to Mechanistic Synthesis

The Discussion section currently lists potential mechanisms (e.g., endothelial dysfunction, immune activation) in a generic manner that is disconnected from the specific findings in the Results. The logic needs to flow from "Specific Observation" to "Specific Mechanism." The authors must move beyond general pathogenesis and use the data to drive the mechanistic hypothesis. For example:

(1) The results show that SEOV is associated with a high rate of macroscopic haematuria (19.8%). The authors should specifically discuss why SEOV might cause more severe renal microvascular damage or localized coagulopathy compared to PUUV (e.g., differences in receptor tropism, vascular bed specificity, or inflammatory response intensity).

(2) The finding of significantly elevated D-dimer with relatively preserved fibrinogen and PT is a distinct phenotype. The authors should discuss whether this indicates a "localized" fibrinolysis/coagulation within the renal microcirculation or a unique form of endotheliopathy-driven coagulopathy, rather than systemic consumptive DIC.

2. Enhancing Data Synthesis and Addressing Heterogeneity

While I acknowledge that a formal meta-analysis was not feasible, the heavy reliance on "weighted averages" is scientifically limiting given the acknowledged high heterogeneity. A single average value can be misleading when it collapses data from vastly different cohorts (e.g., mild outpatient PUUV vs. severe ICU HTNV cases).

(1) I recommend providing Box plots or Forest plots (without a pooled estimate) for key parameters such as Platelet counts and APTT in the Supplementary Materials. This is essential for readers to understand the range and spread of the data across studies, rather than just the central tendency.

(2) Instead of global averages, the authors should provide a clearer comparative analysis between "Severe" and "Non-severe" cohorts (where data allows) to highlight the dynamic and severity-dependent nature of these haemostatic disturbances.

3. Clinical Utility and Actionable Insights

As a comprehensive review of 7,950 cases, the manuscript should provide actionable insights for the clinical and research communities. Currently, the conclusion is too generic.

(1) Please include a "Summary of Findings" table utilizing the GRADE framework (or a similar structured approach). This should provide a snapshot of the quality of evidence and the certainty of the findings for each major clinical outcome (e.g., bleeding risk, severity of thrombocytopenia).

(2) Based on this large-scale synthesis, which laboratory marker(s) should be prioritized by clinicians as the earliest or most reliable predictor of progression to severe bleeding? The conclusion needs to offer more than just a call for "more research"; it should provide a data-driven roadmap for clinical monitoring.

**********

PLOS authors have the option to publish the peer review history of their article (what does this mean?). If published, this will include your full peer review and any attached files.

If you choose “no”, your identity will remain anonymous but your review may still be made public.

**Do you want your identity to be public for this peer review?**  For information about this choice, including consent withdrawal, please see our Privacy Policy.

Reviewer #1: No

Reviewer #2: No

Reviewer #3: No

[NOTE: If reviewer comments were submitted as an attachment file, they will be attached to this email and accessible via the submission site. Please log into your account, locate the manuscript record, and check for the action link "View Attachments". If this link does not appear, there are no attachment files.]

**Figure resubmission:**  While revising your submission, we strongly recommend that you use PLOS’s NAAS tool (https://ngplosjournals.pagemajik.ai/artanalysis) to test your figure files. NAAS can convert your figure files to the TIFF file type and meet basic requirements (such as print size, resolution), or provide you with a report on issues that do not meet our requirements and that NAAS cannot fix.

After uploading your figures to PLOS’s NAAS tool - https://ngplosjournals.pagemajik.ai/artanalysis, NAAS will process the files provided and display the results in the "Uploaded Files" section of the page as the processing is complete. If the uploaded figures meet our requirements (or NAAS is able to fix the files to meet our requirements), the figure will be marked as "fixed" above. If NAAS is unable to fix the files, a red "failed" label will appear above. When NAAS has confirmed that the figure files meet our requirements, please download the file via the download option, and include these NAAS processed figure files when submitting your revised manuscript.

**Reproducibility:**

To enhance the reproducibility of your results, we recommend that authors of applicable studies deposit laboratory protocols in protocols.io, where a protocol can be assigned its own identifier (DOI) such that it can be cited independently in the future. Additionally, PLOS ONE offers an option to publish peer-reviewed clinical study protocols. Read more information on sharing protocols at https://plos.org/protocols?utm_medium=editorial-email&utm_source=authorletters&utm_campaign=protocols

# Author comment: Author response to Decision Letter 2

**DOI:** 10.1371/journal.pntd.0014524.r004

16 Jun 2026

Attachment Submitted filename: Response_to_Reviewers_auresp_2.docx

# Peer review: Decision Letter 2

**DOI:** 10.1371/journal.pntd.0014524.r005

**Authors:** Shu Shen, David Safronetz

30 Jun 2026

Dear Dr Riley,

We are pleased to inform you that your manuscript 'Bleeding in haemorrhagic fever with renal syndrome: A systematic review characterising the loss of haemostasis in hantavirus infections' has been provisionally accepted for publication in PLOS Neglected Tropical Diseases.

Before your manuscript can be formally accepted you will need to complete some formatting changes, which you will receive in a follow up email. A member of our team will be in touch with a set of requests.

Please note that your manuscript will not be scheduled for publication until you have made the required changes, so a swift response is appreciated.

IMPORTANT: The editorial review process is now complete. PLOS will only permit corrections to spelling, formatting or significant scientific errors from this point onwards. Requests for major changes, or any which affect the scientific understanding of your work, will cause delays to the publication date of your manuscript.

Should you, your institution's press office or the journal office choose to press release your paper, you will automatically be opted out of early publication. We ask that you notify us now if you or your institution is planning to press release the article. All press must be co-ordinated with PLOS.

Thank you again for supporting Open Access publishing; we are looking forward to publishing your work in PLOS Neglected Tropical Diseases.

Best regards,

Shu Shen

Academic Editor

PLOS Neglected Tropical Diseases

David Safronetz

Section Editor

PLOS Neglected Tropical Diseases

Shaden Kamhawi

co-Editor-in-Chief

PLOS Neglected Tropical Diseases

orcid.org/0000-0003-4304-636XX

Paul Brindley

co-Editor-in-Chief

PLOS Neglected Tropical Diseases

orcid.org/0000-0003-1765-0002

***********************************************************

Reviewer's Responses to Questions

**Key Review Criteria Required for Acceptance?**

As you describe the new analyses required for acceptance, please consider the following:

**Methods**

-Are the objectives of the study clearly articulated with a clear testable hypothesis stated?

-Is the study design appropriate to address the stated objectives?

-Is the population clearly described and appropriate for the hypothesis being tested?

-Is the sample size sufficient to ensure adequate power to address the hypothesis being tested?

-Were correct statistical analysis used to support conclusions?

-Are there concerns about ethical or regulatory requirements being met?

Reviewer #3: (No Response)

**********

**Results**

-Does the analysis presented match the analysis plan?

-Are the results clearly and completely presented?

-Are the figures (Tables, Images) of sufficient quality for clarity?

Reviewer #3: (No Response)

**********

**Conclusions**

-Are the conclusions supported by the data presented?

-Are the limitations of analysis clearly described?

-Do the authors discuss how these data can be helpful to advance our understanding of the topic under study?

-Is public health relevance addressed?

Reviewer #3: (No Response)

**********

**Editorial and Data Presentation Modifications?**

Use this section for editorial suggestions as well as relatively minor modifications of existing data that would enhance clarity. If the only modifications needed are minor and/or editorial, you may wish to recommend “Minor Revision” or “Accept”.

Reviewer #3: (No Response)

**********

**Summary and General Comments**

Use this section to provide overall comments, discuss strengths/weaknesses of the study, novelty, significance, general execution and scholarship. You may also include additional comments for the author, including concerns about dual publication, research ethics, or publication ethics. If requesting major revision, please articulate the new experiments that are needed.

Reviewer #3: (No Response)

**********

PLOS authors have the option to publish the peer review history of their article (what does this mean?). If published, this will include your full peer review and any attached files.

If you choose “no”, your identity will remain anonymous but your review may still be made public.

**Do you want your identity to be public for this peer review?**  For information about this choice, including consent withdrawal, please see our Privacy Policy.

Reviewer #3: No

# Editor report: Acceptance letter

**DOI:** 10.1371/journal.pntd.0014524.r006

**Authors:** Shu Shen, David Safronetz

Dear Dr Riley,

We are delighted to inform you that your manuscript, "Bleeding in haemorrhagic fever with renal syndrome: A systematic review characterising the loss of haemostasis in hantavirus infections," has been formally accepted for publication in PLOS Neglected Tropical Diseases.

We have now passed your article onto the PLOS Production Department who will complete the rest of the publication process. All authors will receive a confirmation email upon publication.

The corresponding author will soon be receiving a typeset proof for review, to ensure errors have not been introduced during production. Please review the PDF proof of your manuscript carefully, as this is the last chance to correct any scientific or type-setting errors. Please note that major changes, or those which affect the scientific understanding of the work, will likely cause delays to the publication date of your manuscript. Note: Proofs for Front Matter articles (Editorial, Viewpoint, Symposium, Review, etc...) are generated on a different schedule and may not be made available as quickly.

Soon after your final files are uploaded, the early version of your manuscript will be published online unless you opted out of this process. The date of the early version will be your article's publication date. The final article will be published to the same URL, and all versions of the paper will be accessible to readers.

For Research Articles, you will receive an invoice from PLOS for your publication fee after your manuscript has reached the completed accept phase. If you receive an email requesting payment before acceptance or for any other service, this may be a phishing scheme. Learn how to identify phishing emails and protect your accounts at https://explore.plos.org/phishing.

Thank you again for supporting open-access publishing; we are looking forward to publishing your work in PLOS Neglected Tropical Diseases.

Best regards,

Shaden Kamhawi

co-Editor-in-Chief

PLOS Neglected Tropical Diseases

Paul Brindley

co-Editor-in-Chief

PLOS Neglected Tropical Diseases
